# Supplementary material for: Tuning of the Anti-Breast Cancer Activity of Betulinic Acid via Its Conversion to Ionic Liquids
Source: Pharmaceutics. 2024 Apr 3;16(4):496. doi: 10.3390/pharmaceutics16040496 (PMC11053683; doi:10.3390/pharmaceutics16040496)
Supplement: Supplementary file 1 [file pharmaceutics-16-00496-s001.zip › pharmaceutics-2919721-supplementary.pdf]

Supplementary materials

Article

Tuning of the anti-breast cancer activity of betulinic acid via its conversion to ionic liquids

Paula Ossowicz-Rupniewska[1], Joanna Klebeko[1], Irina Georgieva[2], Sonia Apostolova[2], Łukasz Struk[3], Svetla Todinova[2], Rumiana Dimitrova Tzoneva[2], Maya Guncheva[4,*]

[1] Department of Chemical Organic Technology and Polymeric Materials, Faculty of Chemical Technology and Engineering, West Pomeranian University of Technology, Piastów Ave. 42, 71-065 Szczecin, Poland

[2] Institute of Biophysics and Biomedical Engineering, Bulgarian Academy of Sciences, Acad. G. Bonchev Str., Bl. 21, 1113 Sofia, Bulgaria

[3] Department of Organic and Physical Chemistry, Faculty of Chemical Technology and Engineering, West Pomeranian University of Technology, Piastów Ave. 42, 71-065 Szczecin, Poland

[4] Institute of Organic Chemistry with Centre of Phytochemistry, Bulgarian Academy of Sciences, Acad. G. Bonchev Str., Bl. 9, 1113 Sofia, Bulgaria

* Correspondence: maya.guncheva@orgchm.bas.bg; Tel.: +359-2-9606160

Betulinic acid - BA

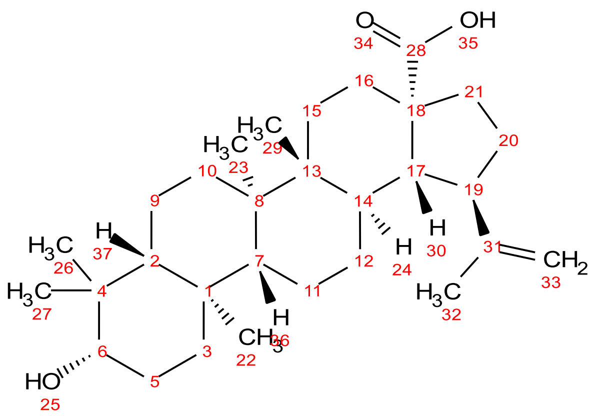

^{1}H NMR (400 MHz, DMSO-d_6) δ 4.67 (d, J = 2.5 Hz, 1H), 4.54 (dd, J = 2.6, 1.5 Hz, 1H), 4.27 (s, 1H), 3.28 (d, J = 44.5 Hz, 2H), 2.93 (td, J = 10.8, 5.7 Hz, 2H), 2.21 (td, J = 12.3, 3.5 Hz, 1H), 2.13 – 2.05 (m, 1H), 1.83 – 1.73 (m, 2H), 1.64 – 1.03 (m, 21H), 0.91 (s, 3H), 0.85 (d, J = 1.5 Hz, 8H), 0.74 (s, 3H), 0.63 (s, 4H). ^{13}C NMR (100 MHz, DMSO-d_6) δ 177.71, 150.80, 110.07, 77.24, 55.89, 55.37, 50.41, 49.02, 47.08, 42.47, 40.72, 38.96, 38.74, 38.05, 37.19, 36.83, 34.40, 32.21, 30.59, 29.68, 28.57, 27.63, 25.56, 20.93, 19.42, 18.44, 16.42, 16.27, 16.21, 14.86. FT–IR: ν (ATR): 3424, 3236, 3074, 1685, 1642, 1484, 1448, 1410, 1388, 1376, 1359, 1319, 1299, 1273, 1235, 1193, 1156, 1136, 1107, 1084, 1075, 1063, 1043, 1033, 1010, 984, 973, 945, 920, 882, 861, 793, 765, 749, 686, 651, 621, 609, 579, 558, 543, 506, 484, 461, 454. Elemental analysis: calc. (%) for $C_{30}H_{48}O_3$ (456.711 g/mol): C (78.90), H (10.59), O (10.51); found: C (78.89), H (10.59), O (10.50).

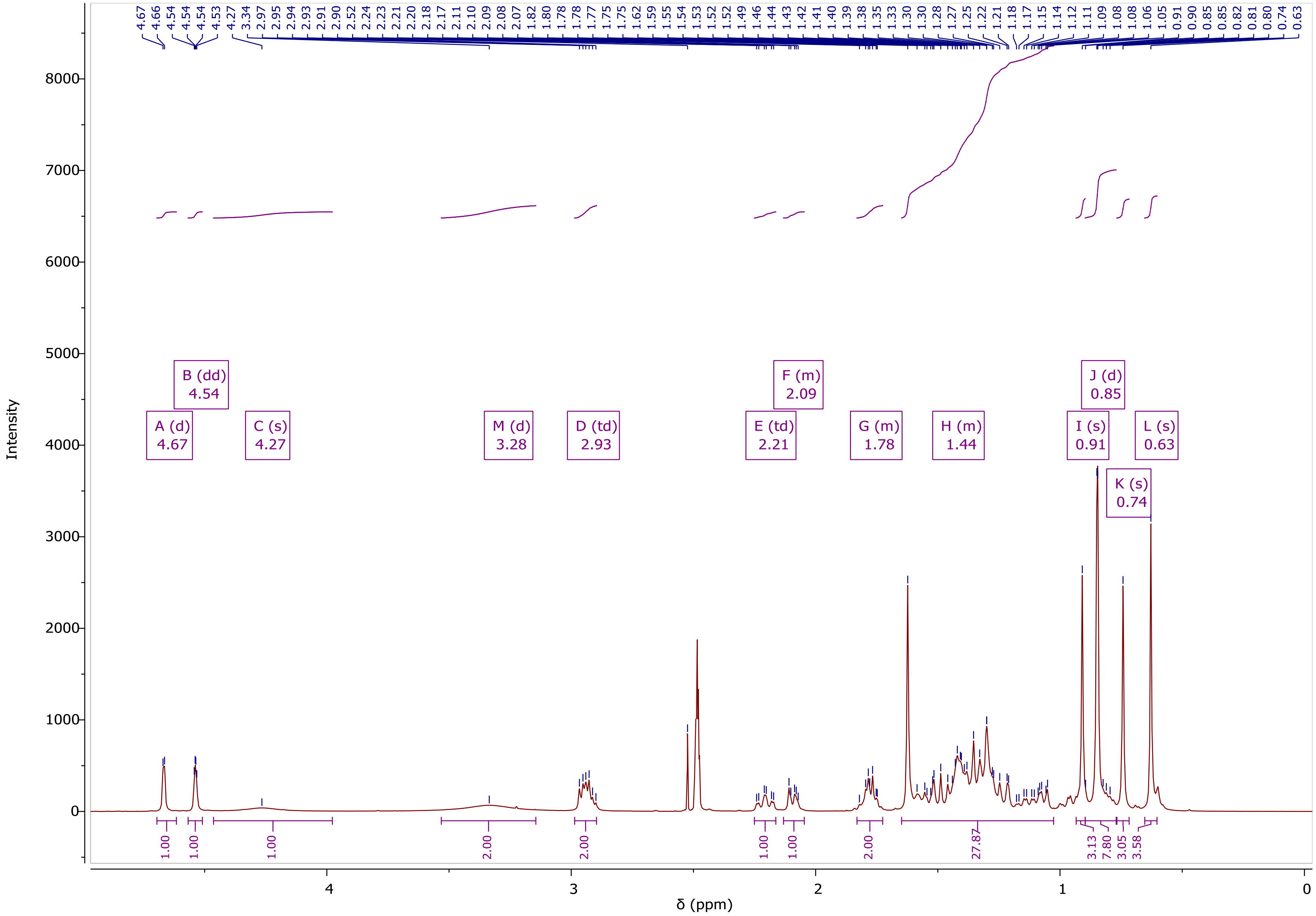

Figure S1. ^{1}H NMR spectrum of BA.

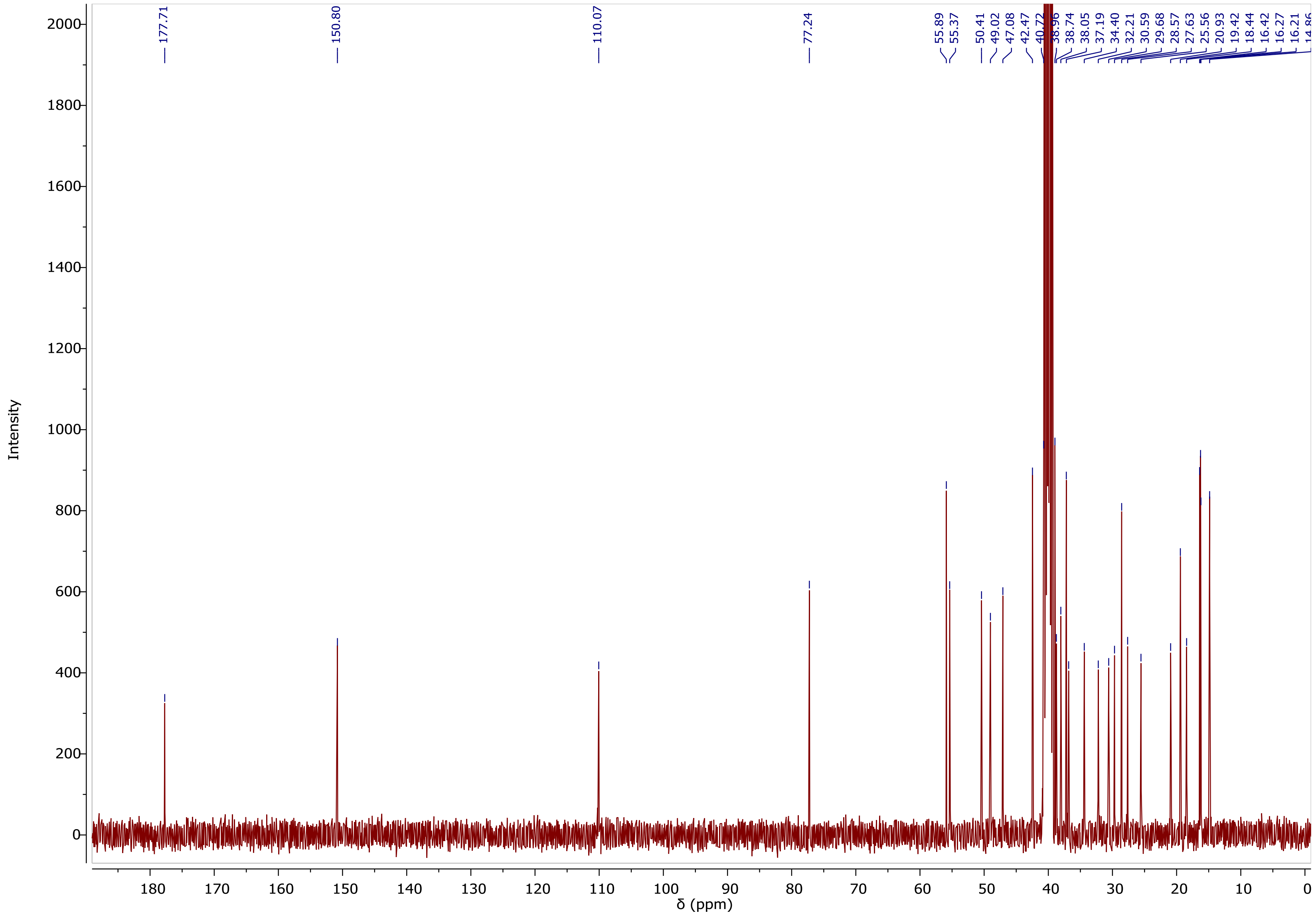

Figure S2. ^{13}C NMR spectrum of BA.

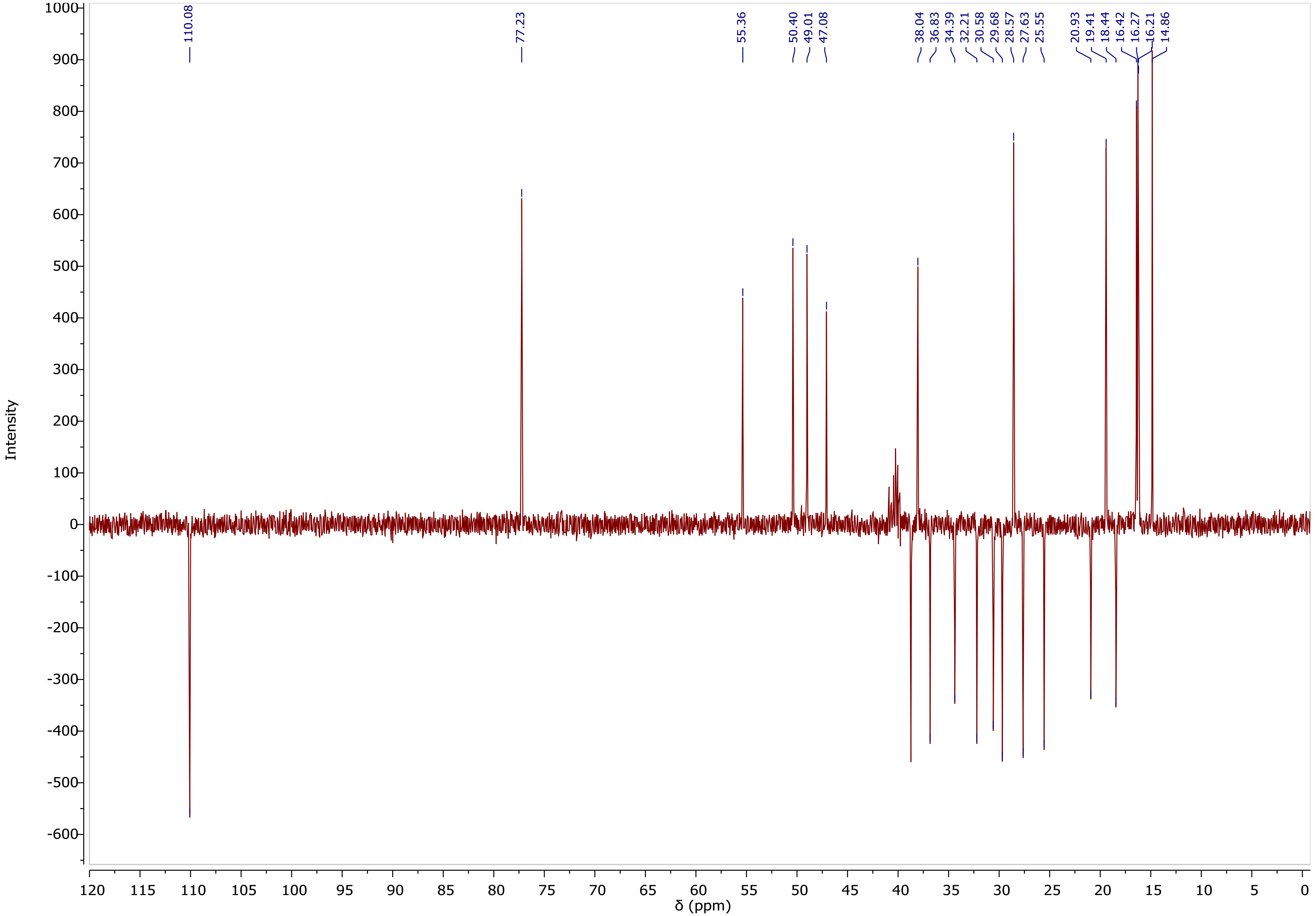

Figure S3. DEPT135 spectrum of BA.

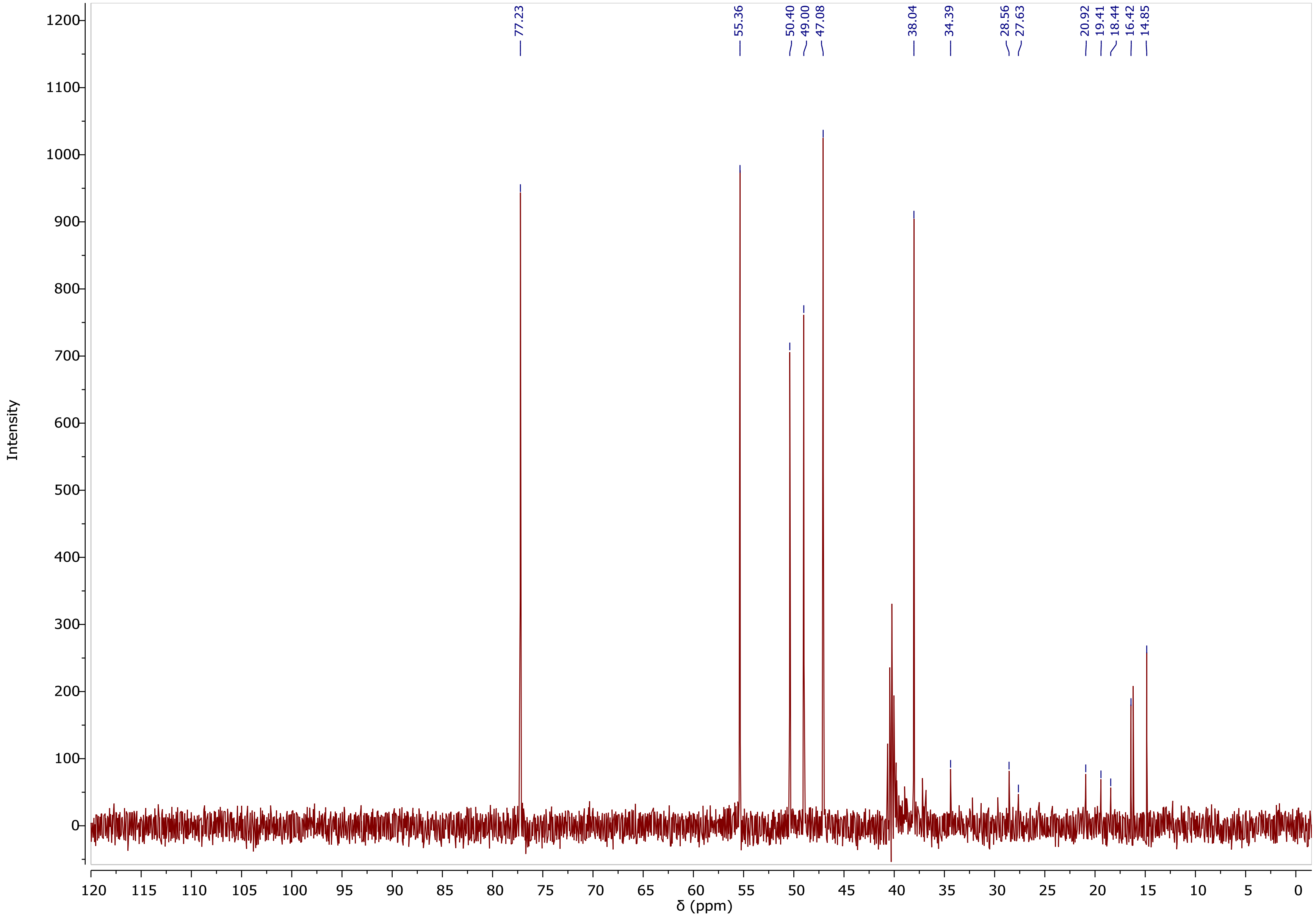

Figure S4. DEPT90 spectrum of BA.

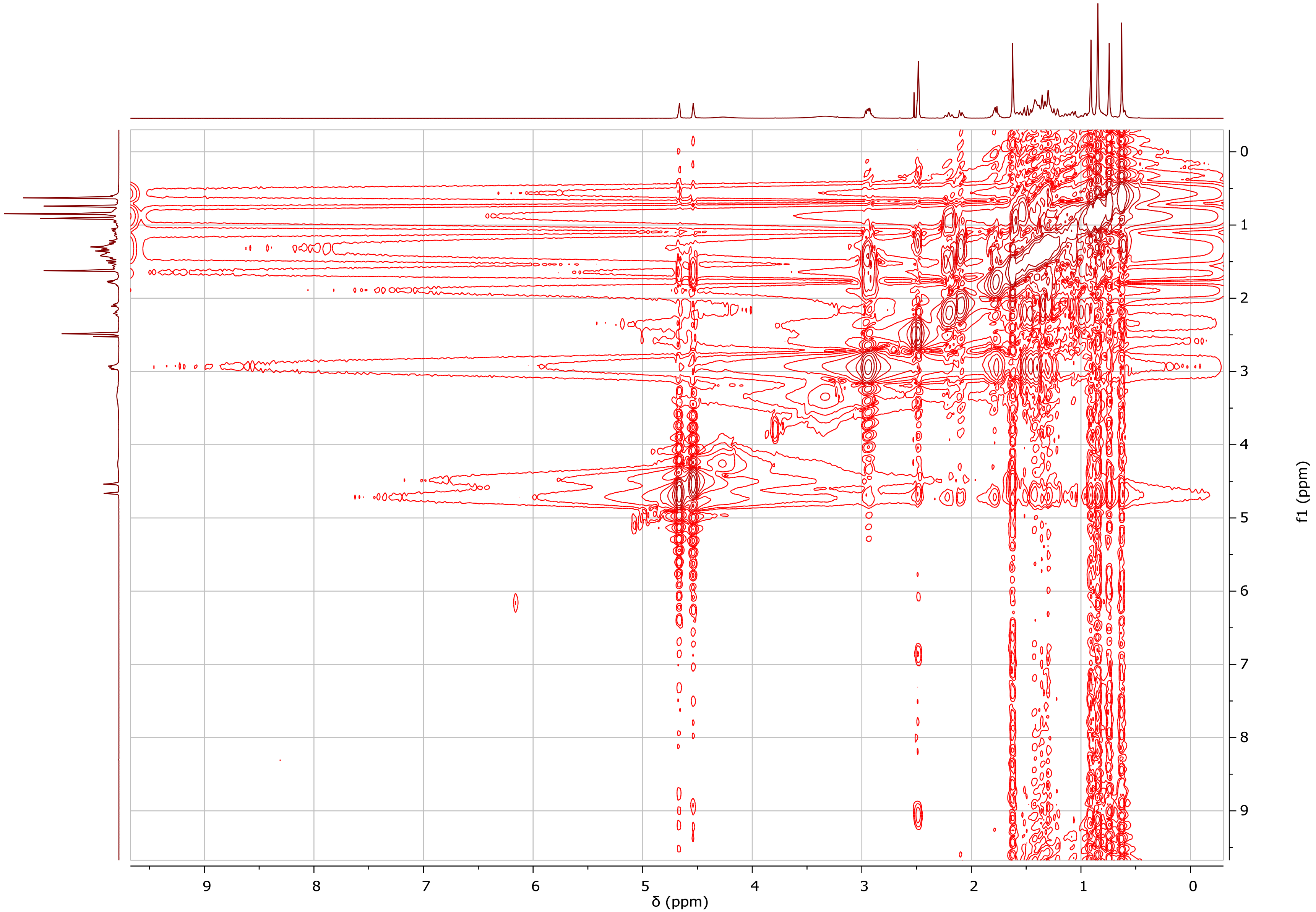

Figure S5. ^{1}H-^{1}H COSY spectrum of BA.

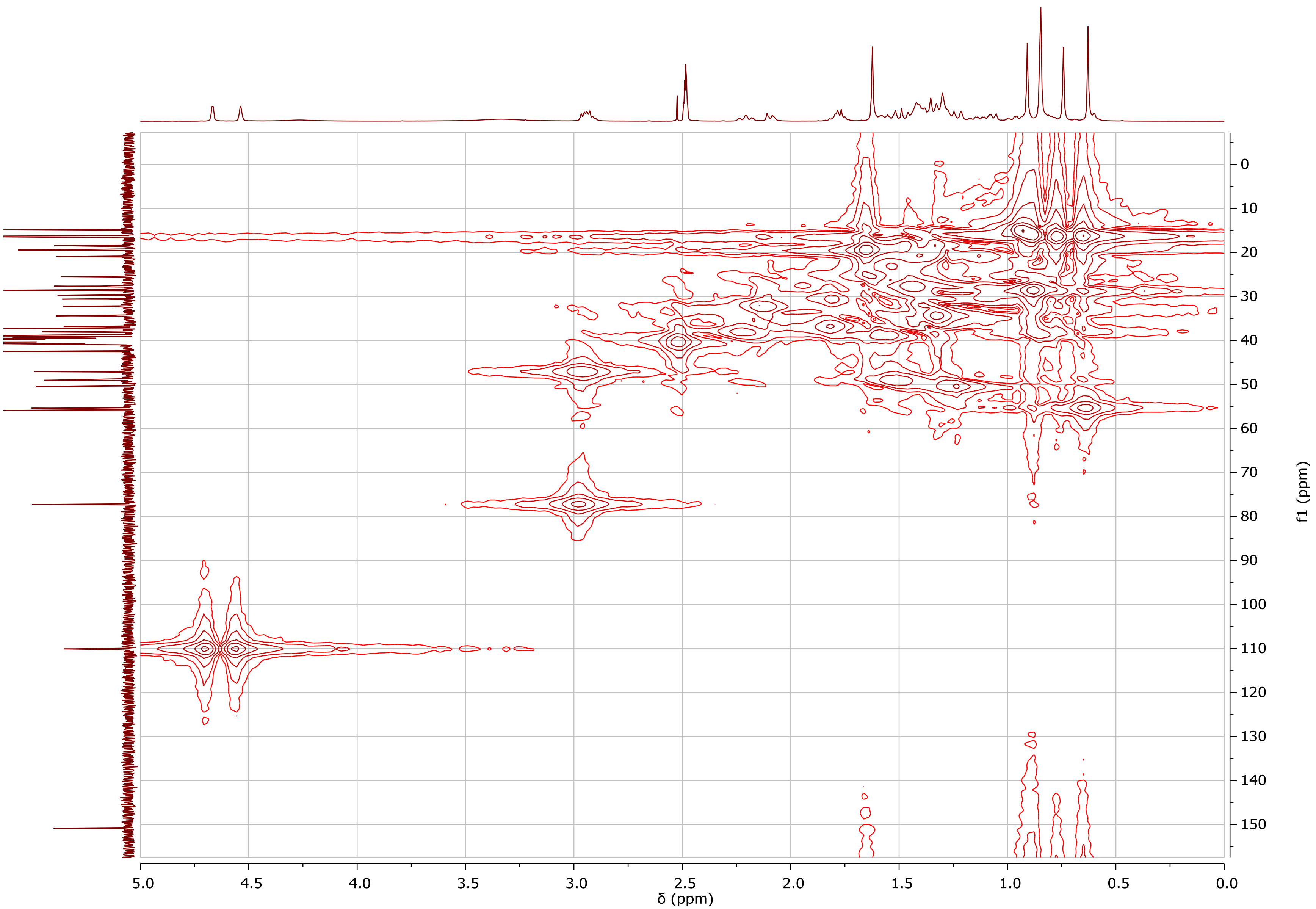

Figure S6. HMQC spectrum of BA.

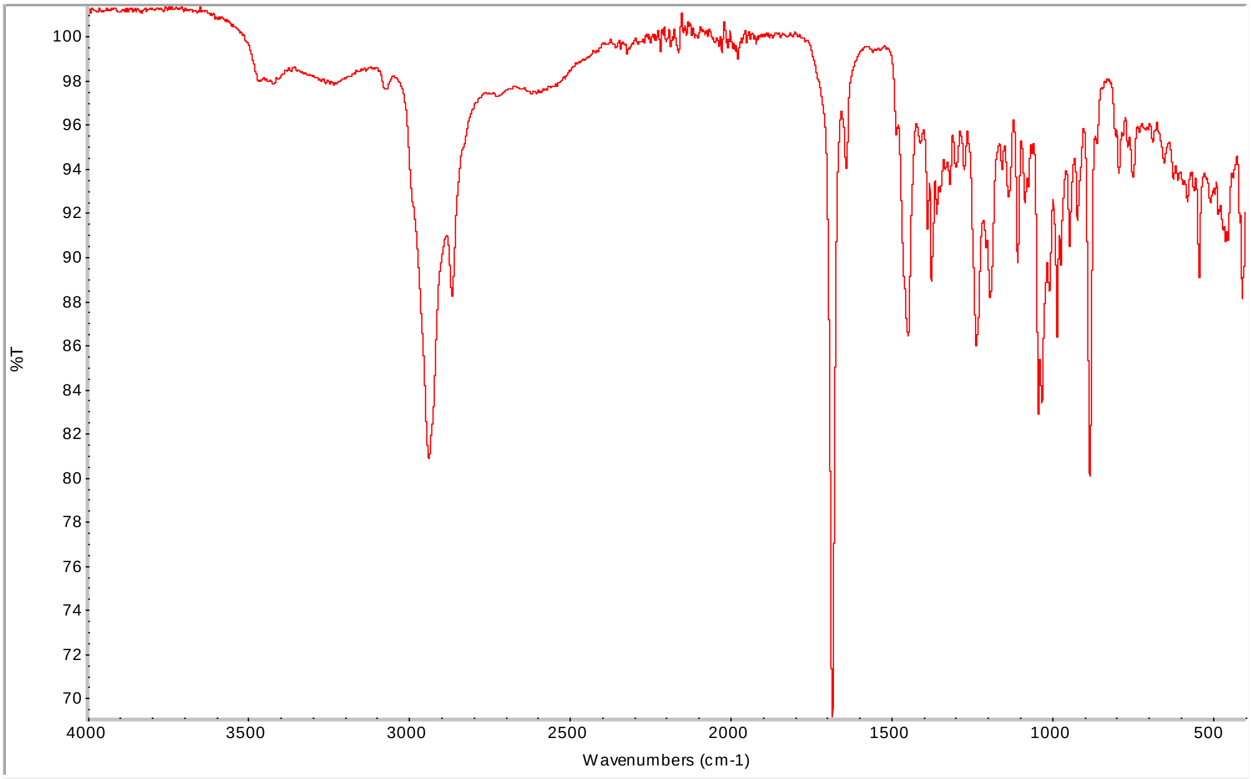

Figure S7. ATR-FTIR spectrum of BA.

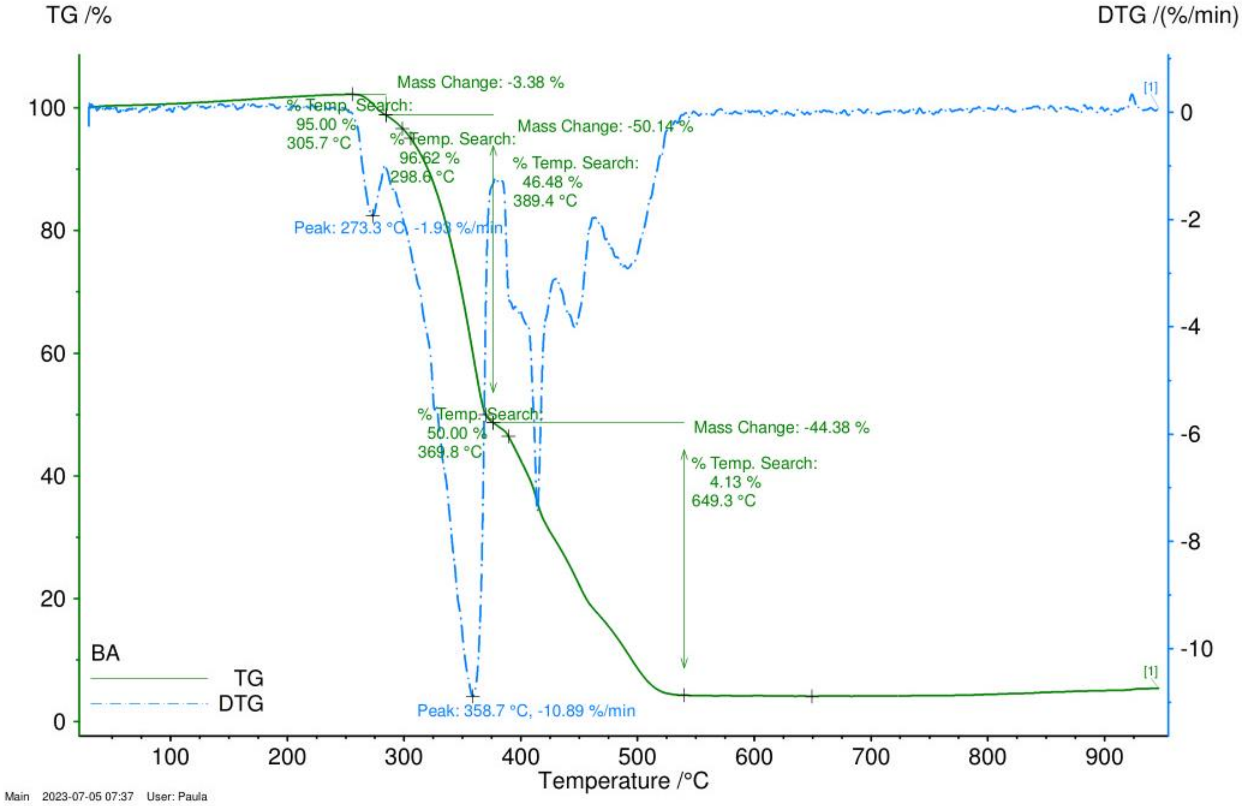

Figure S8. TG, DTG and c-DTA curves of BA.

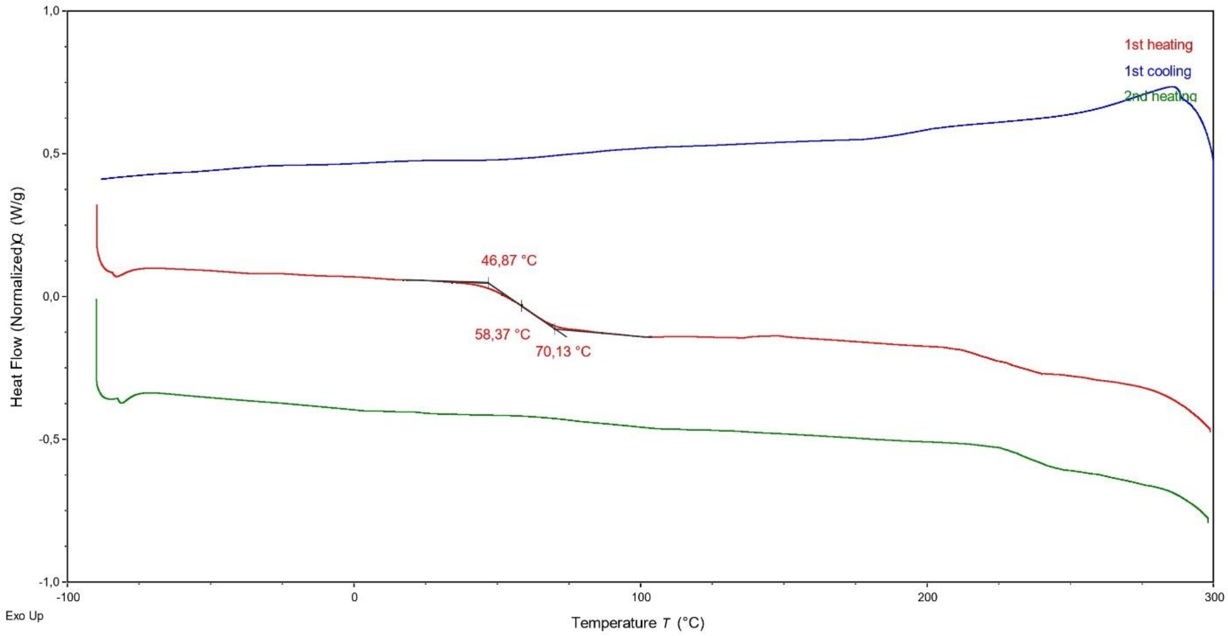

Figure S9. DSC curves of BA.

L-alanine ethyl ester betulinate - [AlaOEt][BA]

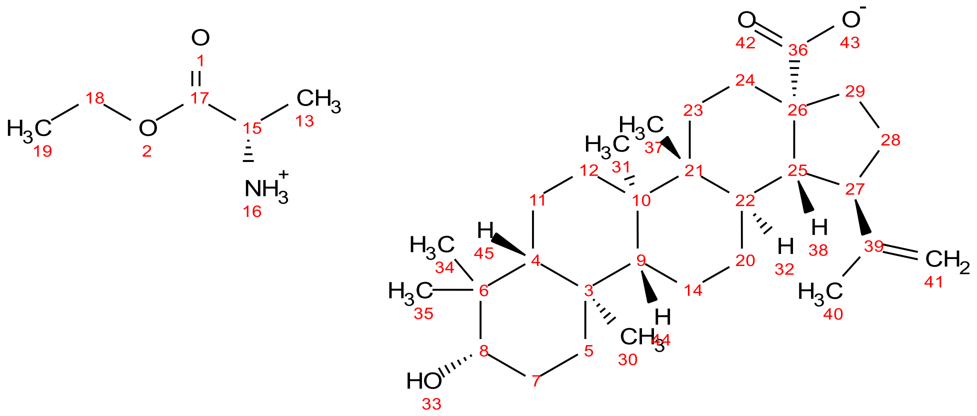

^{1}H NMR (400 MHz, DMSO-d_6) δ 4.68 (d, *J* = 2.5 Hz, 2H), 4.58 – 4.52 (m, 2H), 4.07 (qd, *J* = 7.1, 0.9 Hz, 2H), 3.39 (q, *J* = 7.0 Hz, 1H), 2.97 (dd, *J* = 9.5, 6.6 Hz, 2H), 2.50 (p, *J* = 1.8 Hz, 3H), 2.31 – 2.17 (m, 1H), 2.12 (d, *J* = 11.4 Hz, 1H), 1.79 (q, *J* = 5.2 Hz, 2H), 1.64 (s, 3H), 1.57 – 1.45 (m, 3H), 1.44 (s, 2H), 1.42 – 1.34 (m, 4H), 1.32 (s, 2H), 1.31 – 1.20 (m, 4H), 1.18 (d, *J* = 7.7 Hz, 4H), 1.16 – 1.03 (m, 4H), 0.93 (s, 3H), 0.89 – 0.84 (m, 6H), 0.76 (s, 3H), 0.65 (s, 4H). ^{13}C NMR (100 MHz, DMSO-d_6) δ 177.79, 176.55, 150.83, 110.05, 77.23, 60.40, 55.90, 55.36, 50.41, 49.93, 49.02, 47.08, 42.46, 40.71, 38.96, 38.73, 38.02, 37.19, 36.86, 34.39, 32.25, 30.59, 29.69, 28.56, 27.63, 25.55, 20.98, 19.41, 18.44, 16.42, 16.27, 16.21, 14.85, 14.58. FT–IR: ν (ATR): 2936, 2865, 1747, 1734, 1683, 1643, 1573, 1447, 1386, 1372, 1360, 1334,1298, 1273, 1231, 1212, 1186, 1130, 1106, 1083, 1043, 1031, 1009, 984, 972, 945, 921, 879, 858, 831, 795, 752, 687, 651, 626, 608, 598, 573, 542, 526, 510, 589, 459, 448. Elemental analysis: calc. (%) for $C_{35}H_{59}NO_5$ (573.859 g/mol): C (73.26), H (10.36), N (2.44), O (13.94); found: C (73.27), H (10.36), N (2.45), O (13.94).

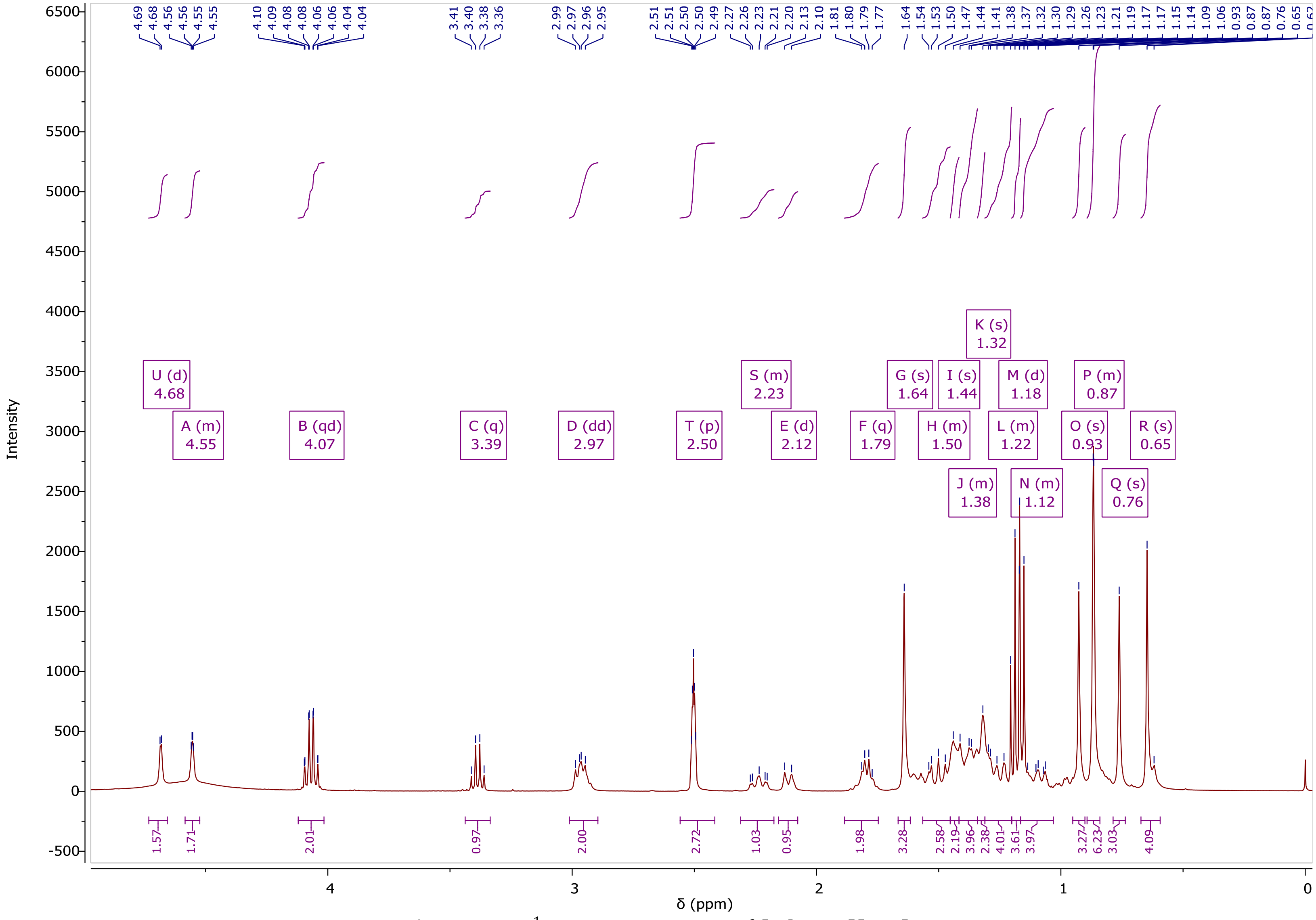

Figure S10. ^{1}H NMR spectrum of [AlaOEt][BA].

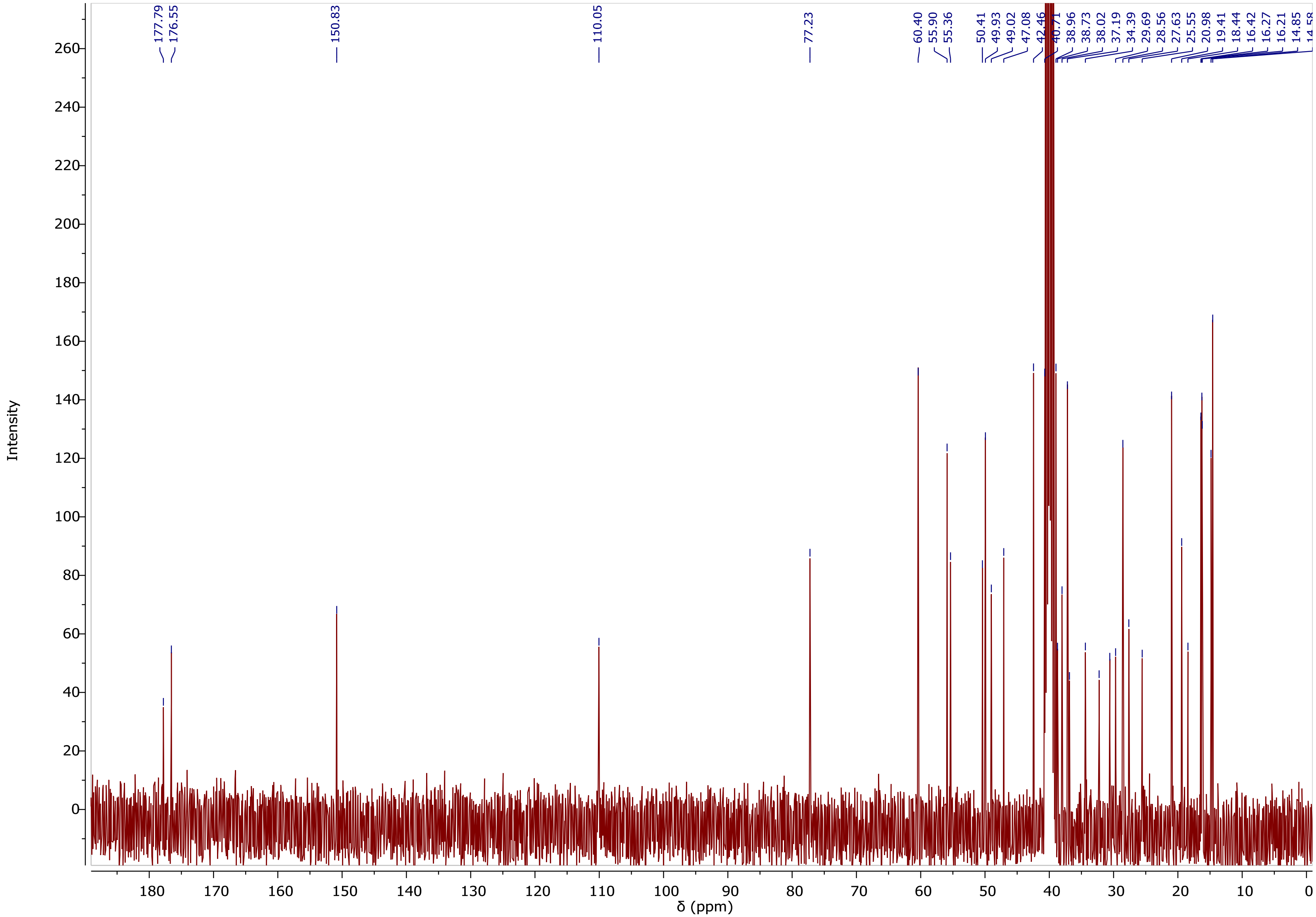

Figure S11. ^{13}C NMR spectrum of [AlaOEt][BA].

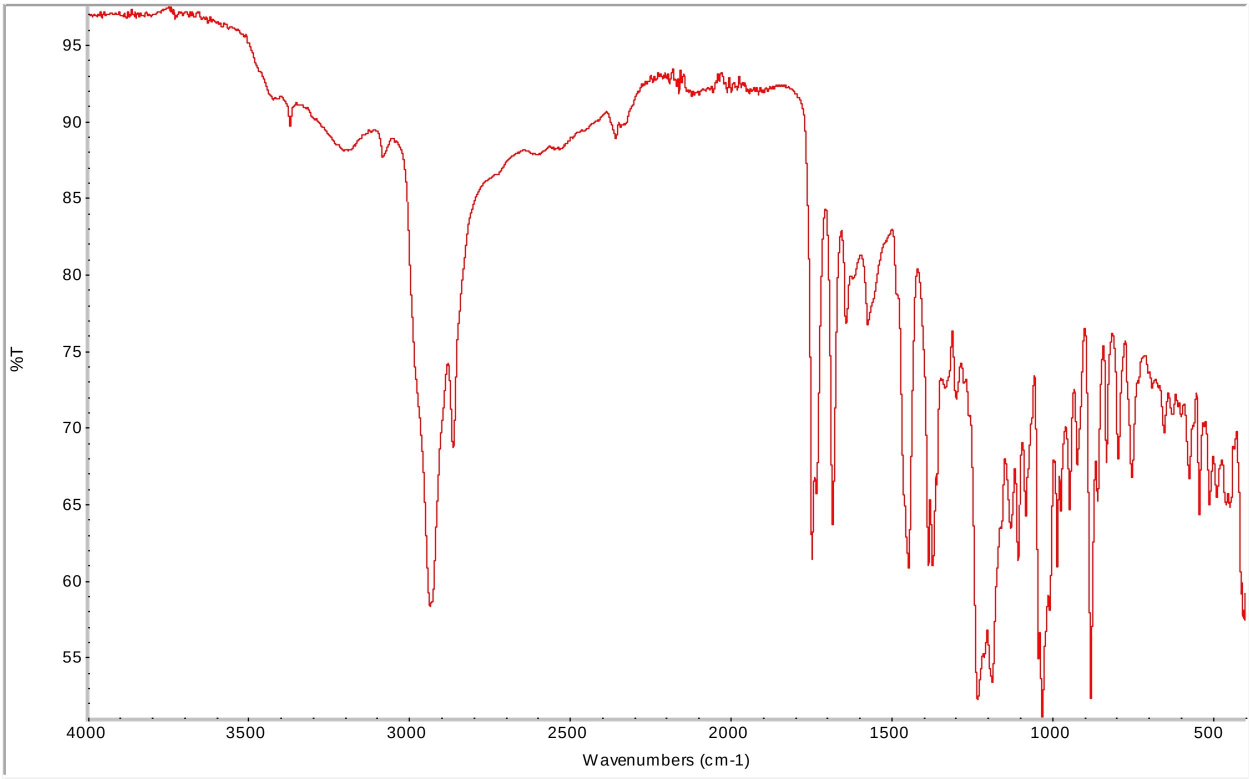

Figure S12. ATR-FTIR spectrum of [AlaOEt][BA].

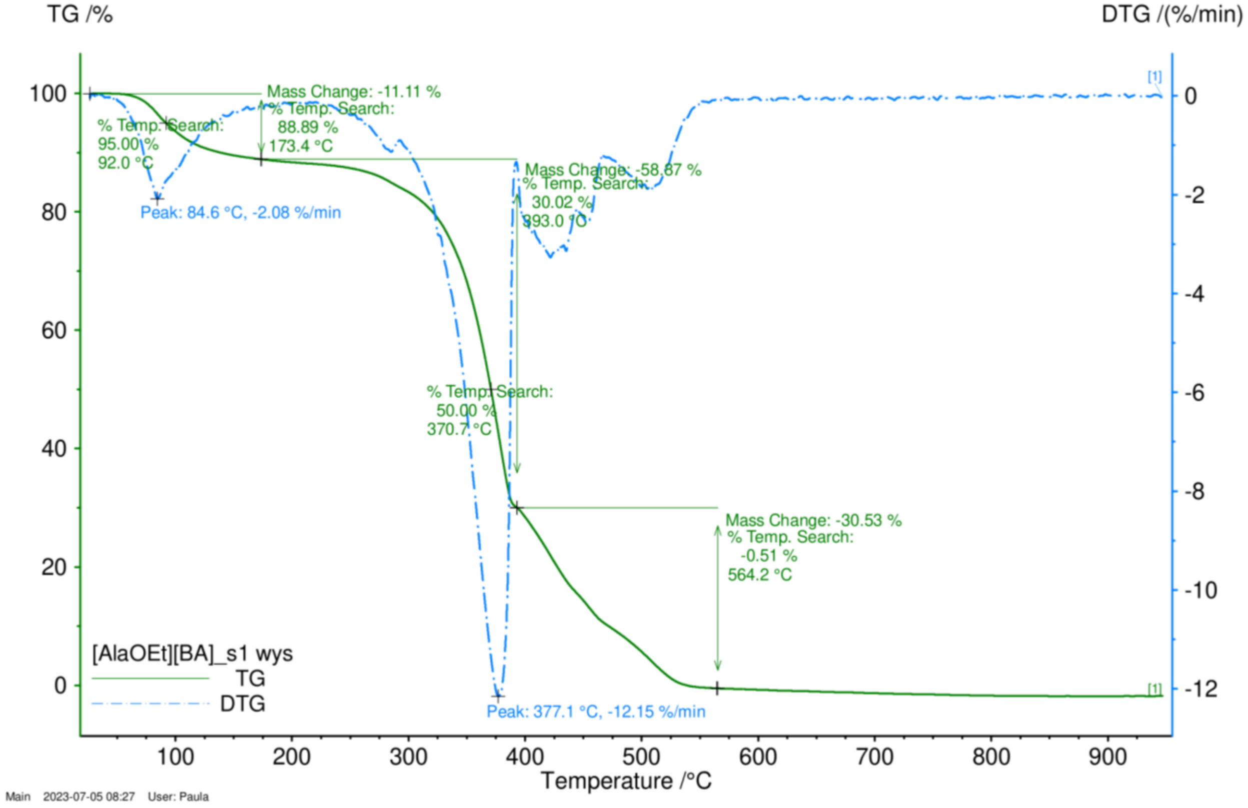

Figure S13. TG, DTG and c-DTA curves of [AlaOEt][BA].

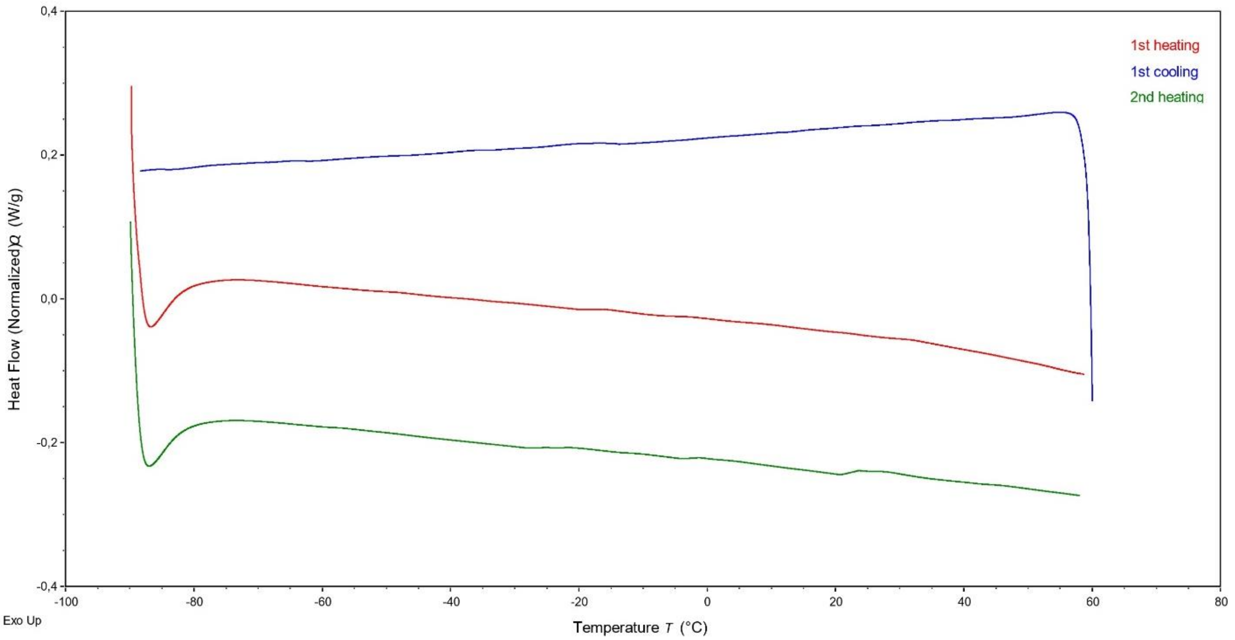

Figure S14. DSC curves of [AlaOEt][BA].

L-valine ethyl ester betulinate - [ValOEt][BA]

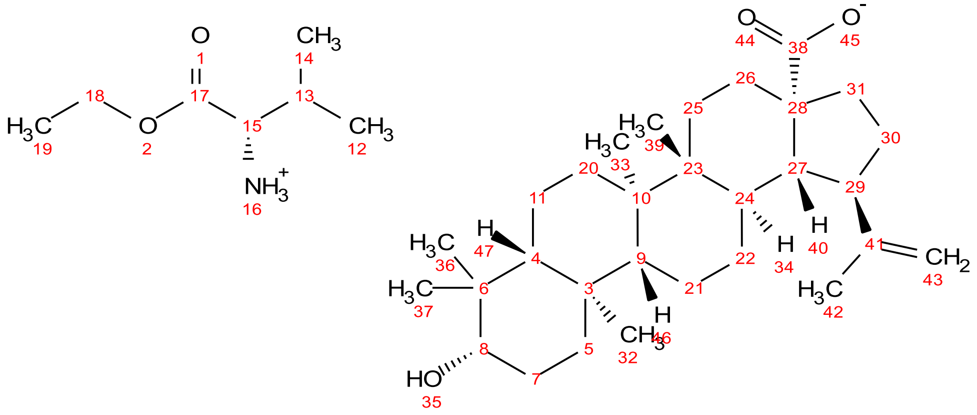

^{1}H NMR (400 MHz, DMSO-d_6) δ 4.73 (s, J = 34.4 Hz, 3H), 4.68 (s, 1H), 4.56 (s, 1H), 4.19 – 3.91 (m, 2H), 3.10 (d, J = 5.4 Hz, 1H), 3.03 – 2.87 (m, 2H), 2.32 – 2.17 (m, 1H), 2.17 – 2.05 (m, 1H), 1.90 – 1.73 (m, 3H), 1.65 (s, 3H), 1.61 – 1.48 (m, 3H), 1.43 (d, J = 11.6 Hz, 3H), 1.35 (d, J = 22.4 Hz, 6H), 1.32 – 1.03 (m, 9H), 0.93 (s, 3H), 0.91 – 0.80 (m, 13H), 0.77 (s, 3H), 0.65 (s, 4H).

^{13}C NMR (100 MHz, DMSO-d_6) δ 177.74, 175.60, 150.78, 110.03, 77.23, 60.25, 60.25, 59.83, 55.89, 55.38, 50.43, 49.02, 47.07, 42.45, 40.72, 40.40, 38.95, 38.75, 38.03, 37.19, 36.85, 34.40, 32.33, 32.24, 30.60, 29.68, 28.55, 27.63, 25.56, 20.94, 19.59, 19.41, 18.44, 17.87, 16.41, 16.25, 16.19, 14.84, 14.65. FT–IR: ν (ATR): 3292, 3076, 2975, 2961, 2937, 2927, 2863, 1743, 1638, 1607, 1483, 1448, 1399, 1386, 1373, 1359, 1341, 1301, 1274, 1229, 1174, 1126, 1105, 1091, 1084, 1043, 1032, 1009, 984, 973, 945, 920, 912, 881, 861, 819, 793, 754, 636, 624, 578, 545, 513, 495, 484, 478, 472, 459, 452. Elemental analysis: calc. (%) for $C_{37}H_{63}NO_5$ (601.913 g/mol): C (73.83), H (10.55), N (2.33), O (13.29); found: C (73.82), H (10.54), N (2.34), O (13.31)

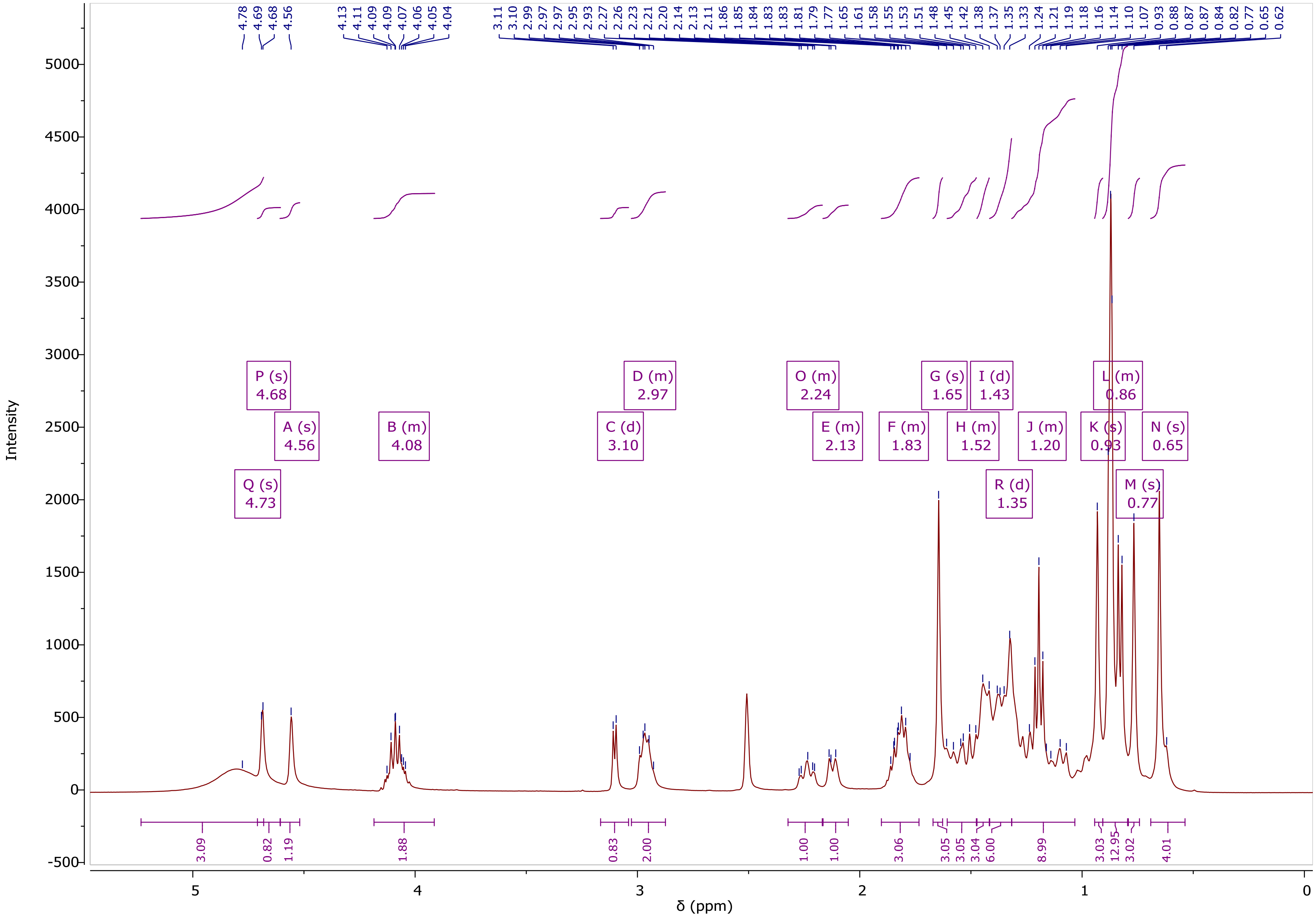

Figure S15. ^{1}H NMR spectrum of [ValOEt][BA].

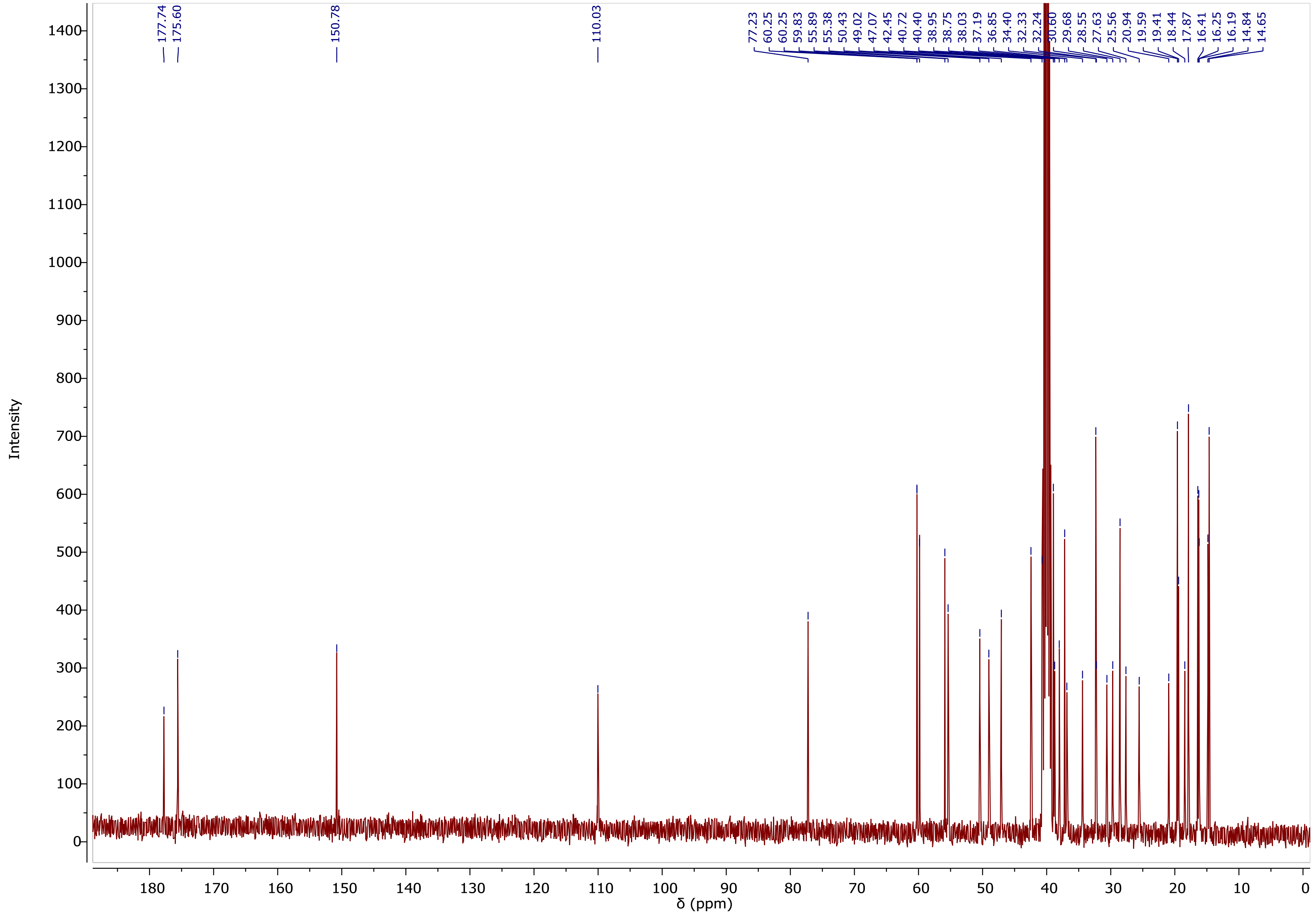

Figure S16. ^{13}C NMR spectrum of [ValOEt][BA].

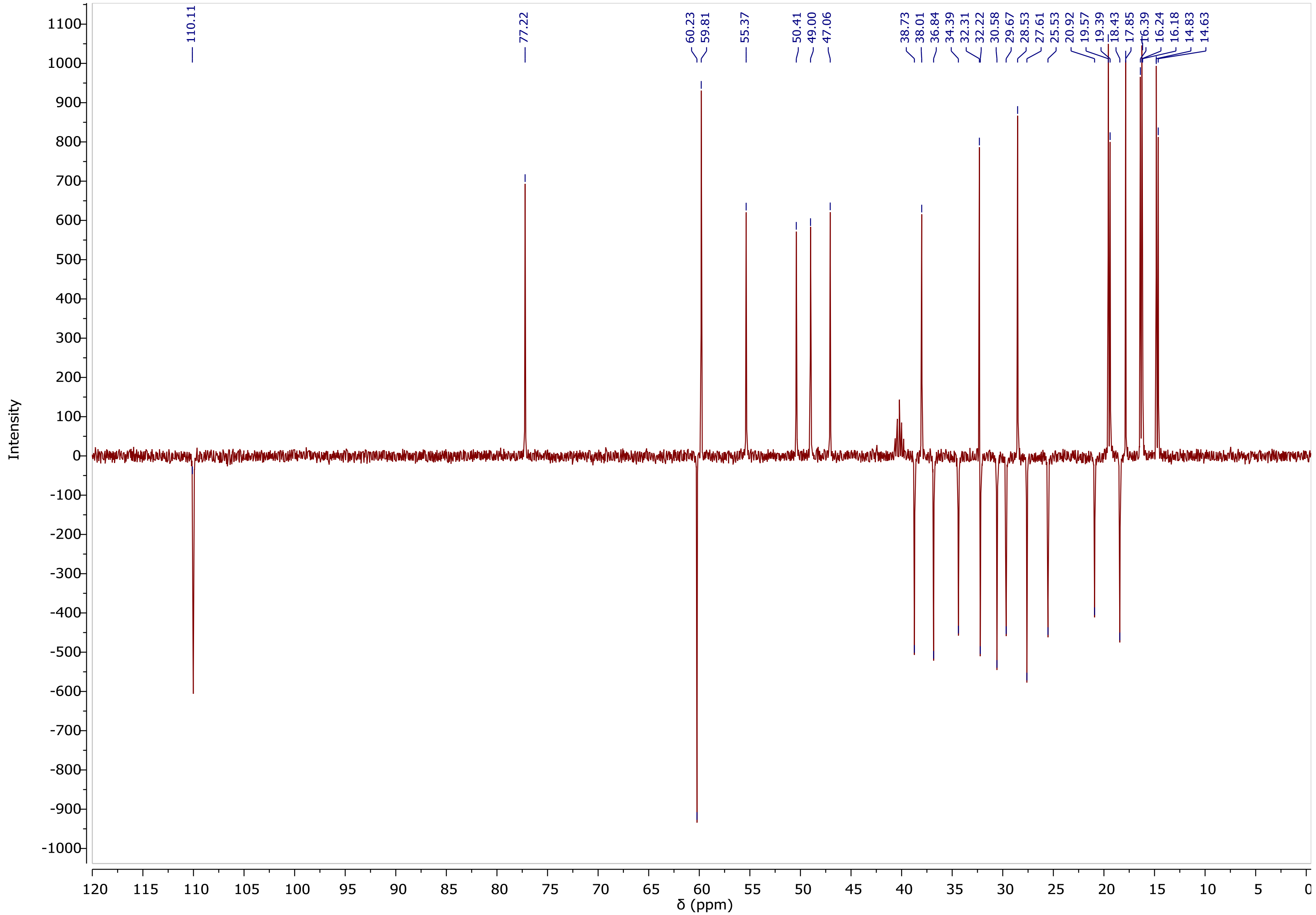

Figure S17. DEPT135 spectrum of [ValOEt][BA].

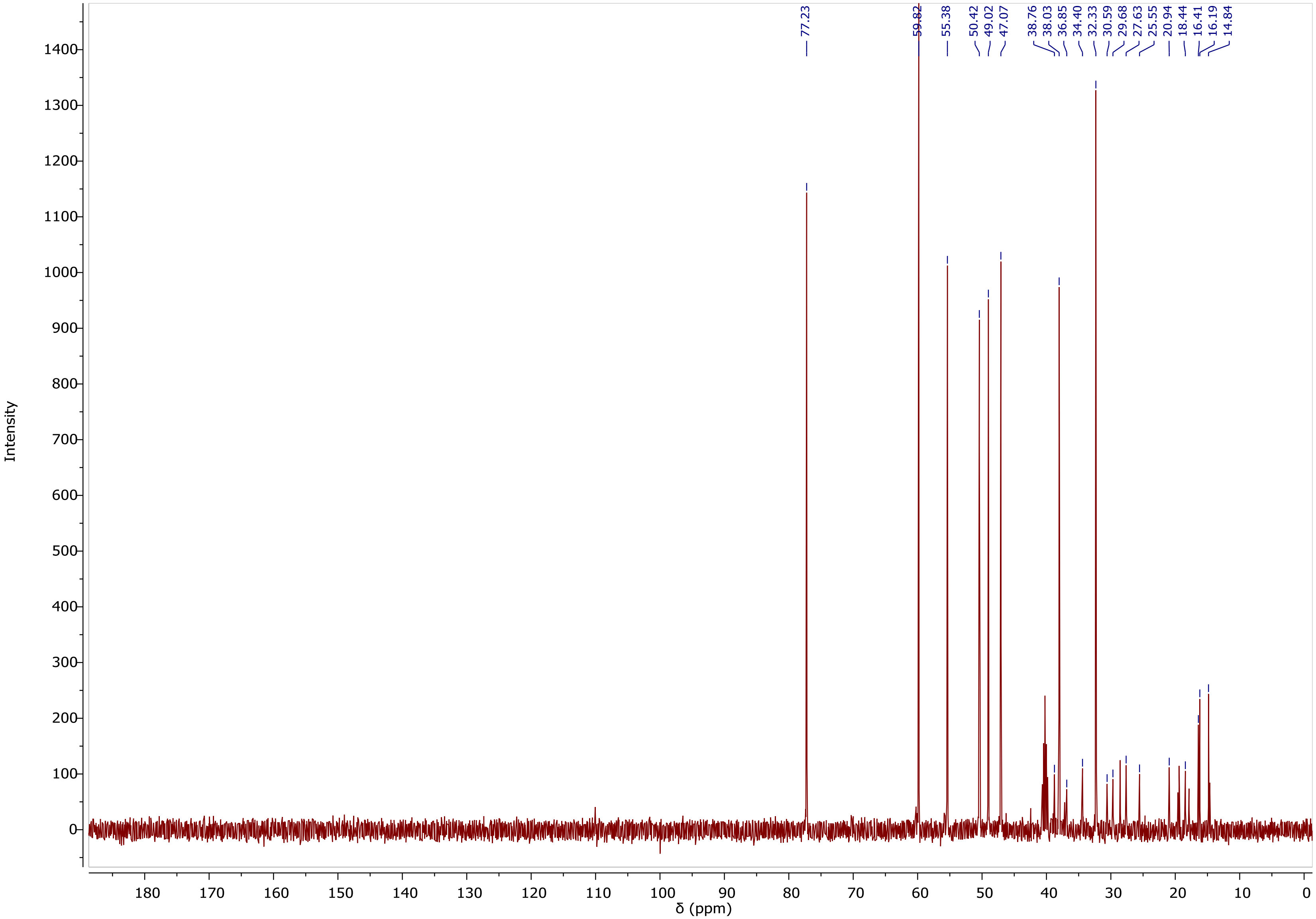

Figure S18. DEPT90 spectrum of [ValOEt][BA].

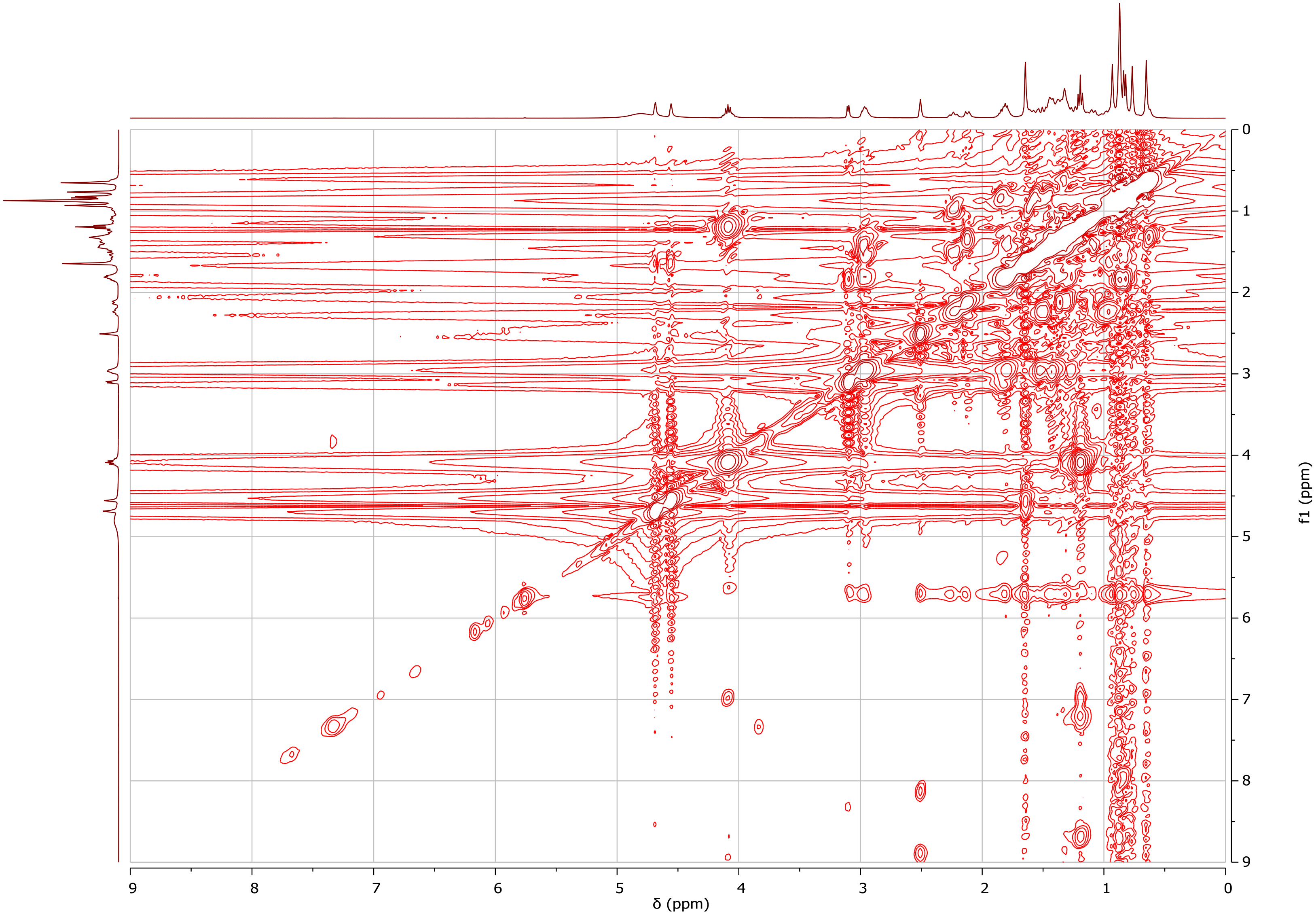

Figure S19. ^{1}H-^{1}H COSY spectrum of [ValOEt][BA].

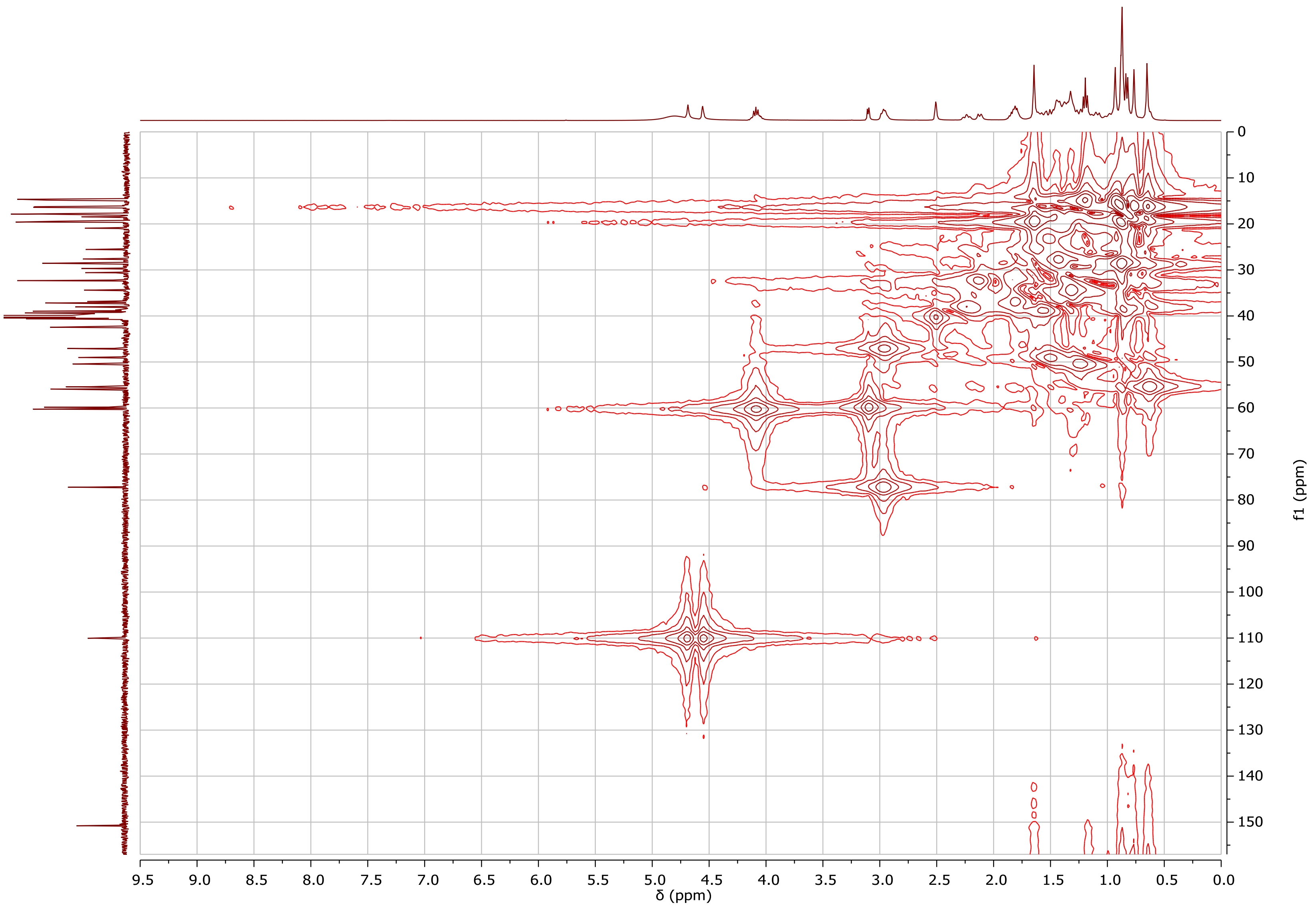

Figure S20. HMQC spectrum of [ValOEt][BA].

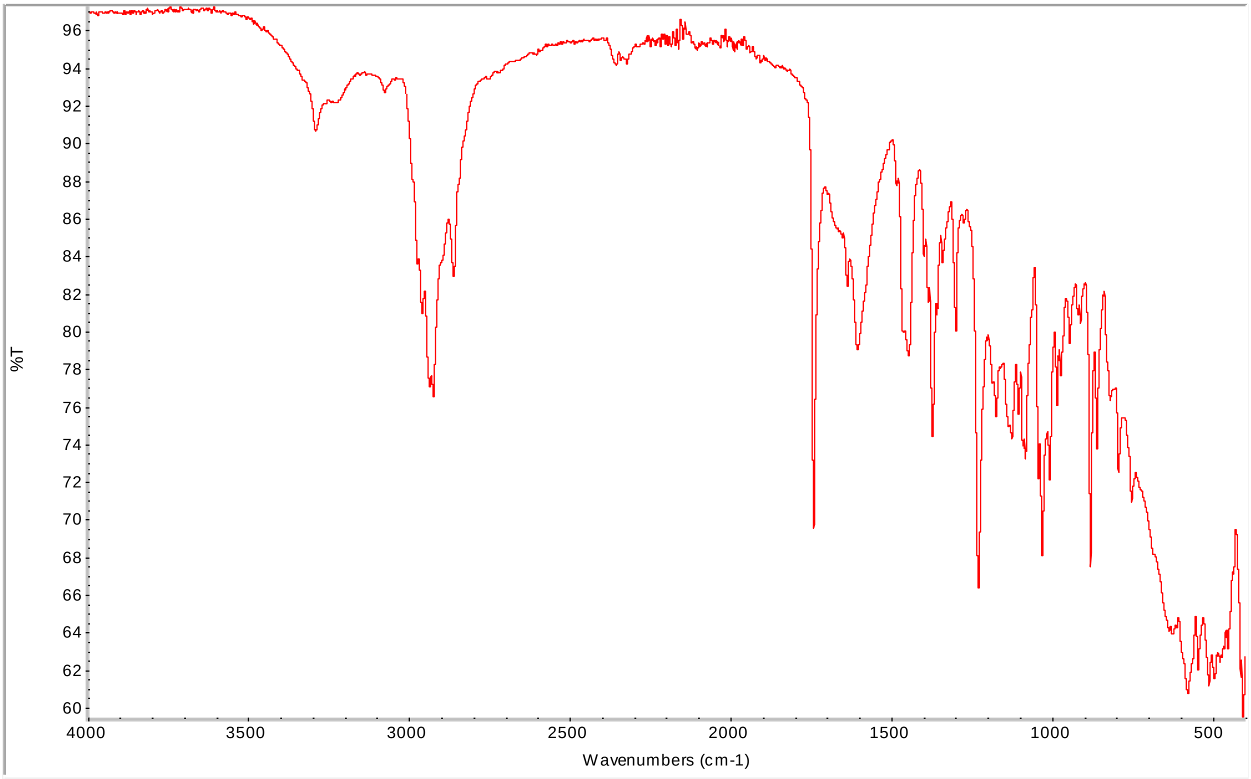

Figure S21. ATR-FTIR spectrum of [ValOEt][BA].

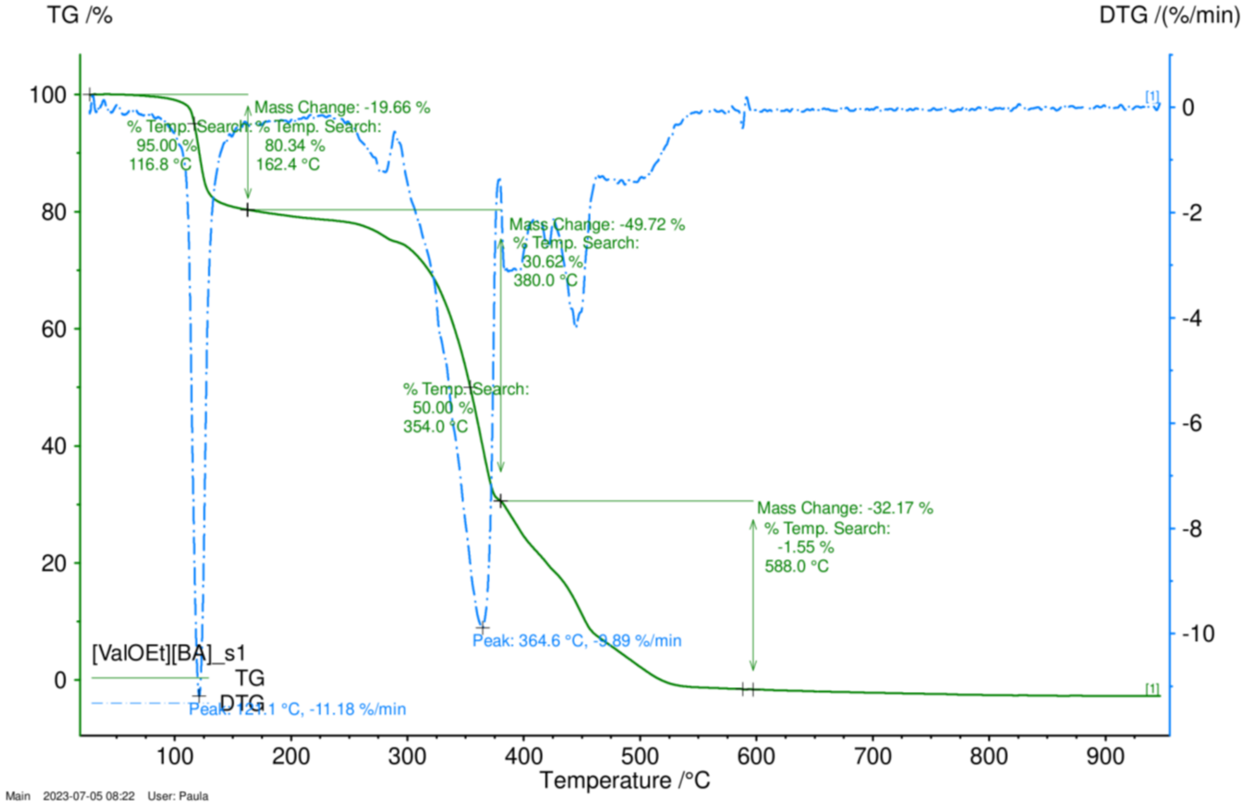

Figure S22. TG, DTG and c-DTA curves of [ValOEt][BA].

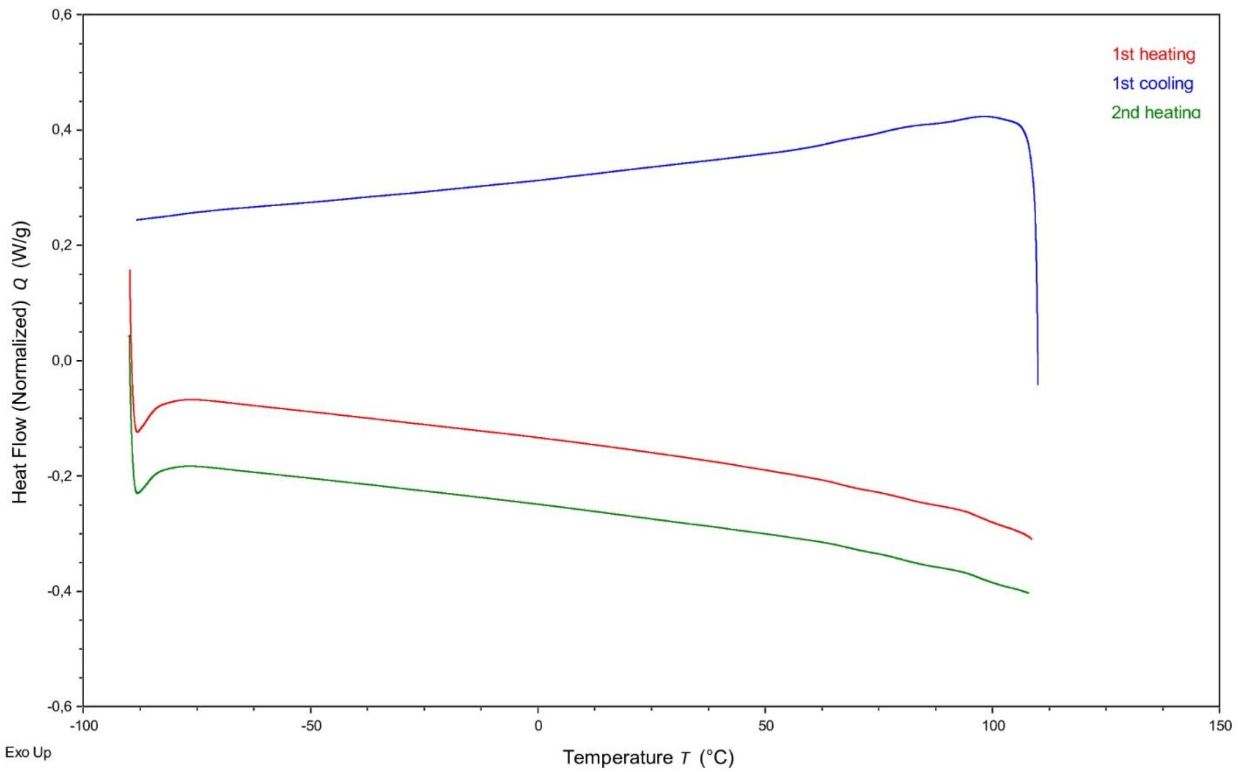

Figure S23. DSC curves of [ValOEt][BA].

L-leucine ethyl ester betulinate - [LeuOEt][BA]

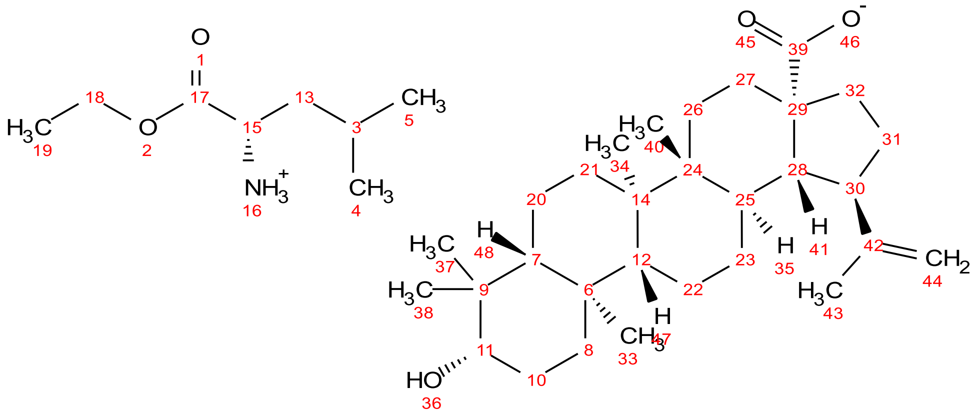

^{1}H NMR (400 MHz, DMSO-d_6) δ 4.44 (d, J = 2.5 Hz, 1H), 4.32 – 4.30 (m, 1H), 3.83 (qd, J = 7.1, 2.1 Hz, 2H), 3.03 (dd, J = 8.6, 5.9 Hz, 1H), 2.71 (td, J = 9.8, 6.0 Hz, 2H), 2.26 (p, J = 1.8 Hz, 3H), 2.04 – 1.93 (m, 1H), 1.87 (d, J = 11.6 Hz, 1H), 1.64 – 1.42 (m, 3H), 1.40 (s, 3H), 1.38 – 1.18 (m, 6H), 1.18 – 0.91 (m, 15H), 0.91 – 0.80 (m, 2H), 0.79 – 0.57 (m, 17H), 0.52 (s, 3H), 0.41 (s, 4H). ^{13}C NMR (100 MHz, DMSO-d_6) δ 177.77, 176.70, 150.82, 110.06, 77.23, 60.31, 55.89, 55.36, 52.83, 50.40, 49.01, 47.08, 44.23, 42.46, 40.71, 38.96, 38.73, 38.02, 37.18, 36.85, 34.39, 32.24, 30.58, 29.68, 28.56, 27.63, 25.55, 24.62, 23.33, 22.31, 20.93, 19.41, 18.43, 16.42, 16.27, 16.21, 14.85, 14.60. FT–IR: ν (ATR): 3425, 3348, 3248, 3077, 2958, 2938, 2867, 1736, 1669, 1642, 1564, 1506, 1482, 1386, 1447, 1412, 1386, 1374, 1365, 1360, 1325, 1303, 1292, 1271, 1240, 1223, 1213, 1178, 1132, 1106, 1084, 1064, 1045, 1029, 1011, 983, 971, 964, 942, 928, 918, 876, 847, 859,

812, 797, 791, 760, 686, 645, 635, 623, 609, 586, 567, 553, 542, 504, 483, 459, 437. Elemental analysis: calc. (%) for $C_{38}H_{65}NO_5$ (615.940 g/mol): C (74.10), H (10.64), N (2.27), O (12.99); found: C (74.10), H (10.63), N (2.27), O (12.99).

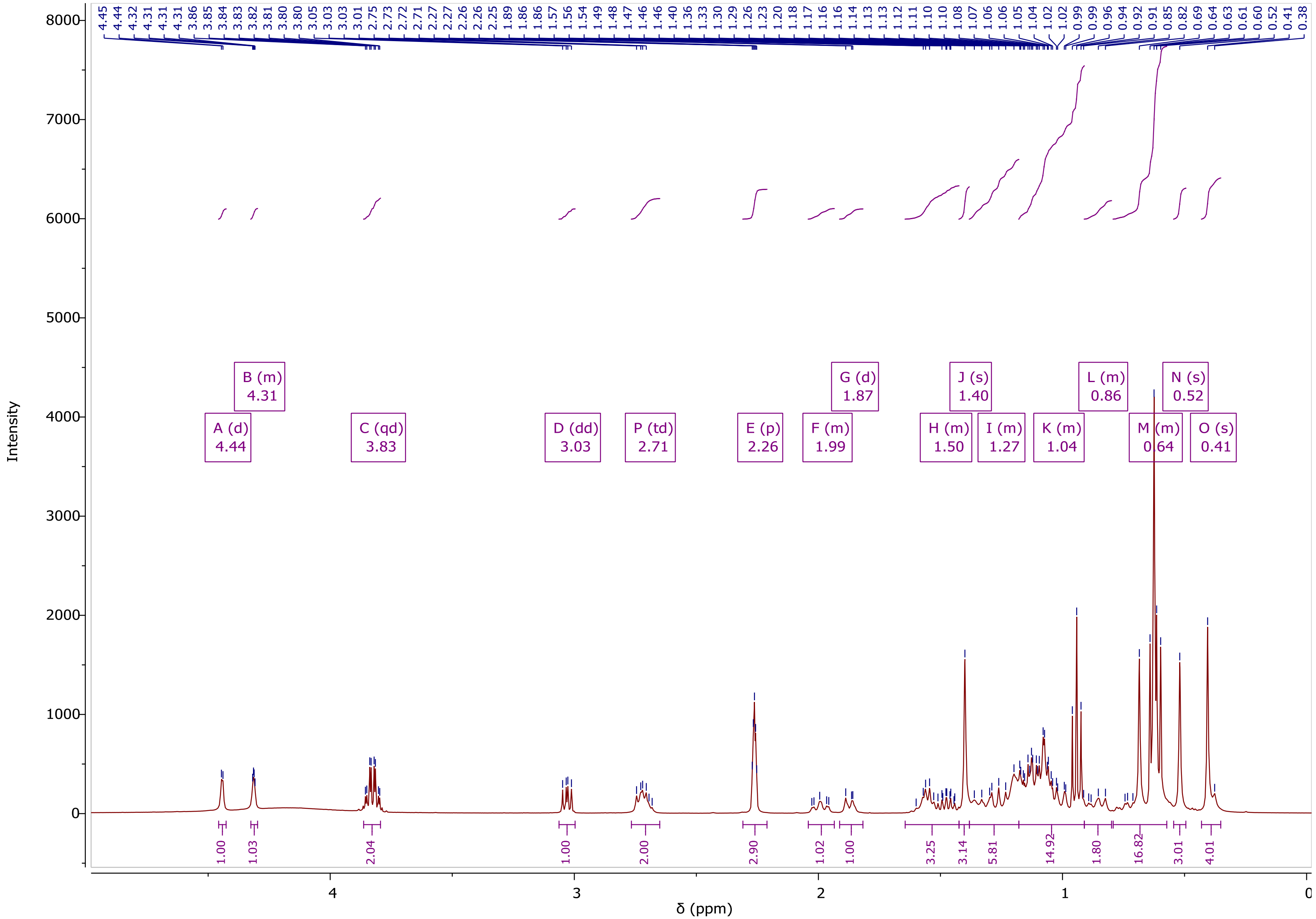

Figure S24. ^{1}H NMR spectrum of [LeuOEt][BA].

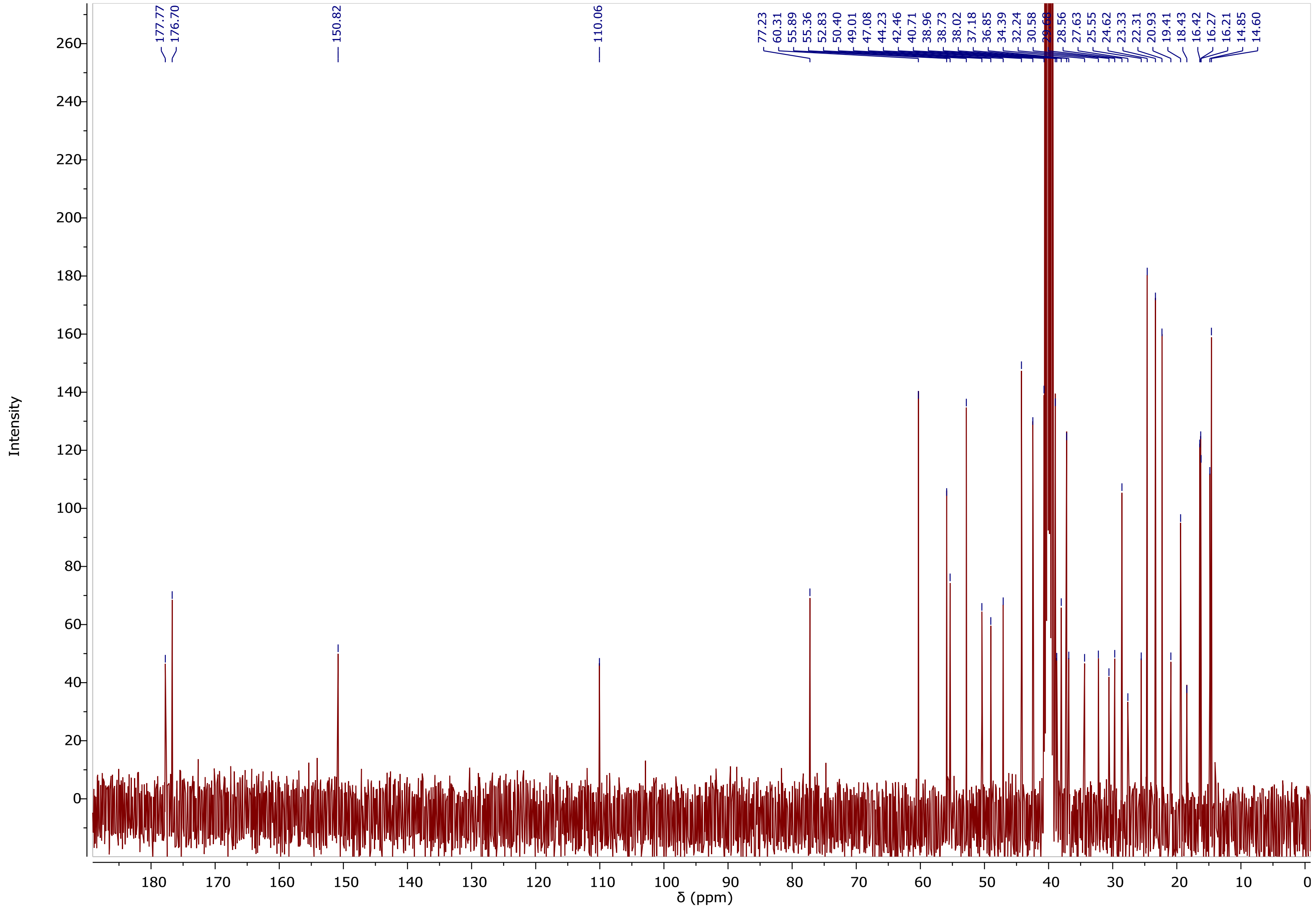

Figure S25. ^{13}C NMR spectrum of [LeuOEt][BA].

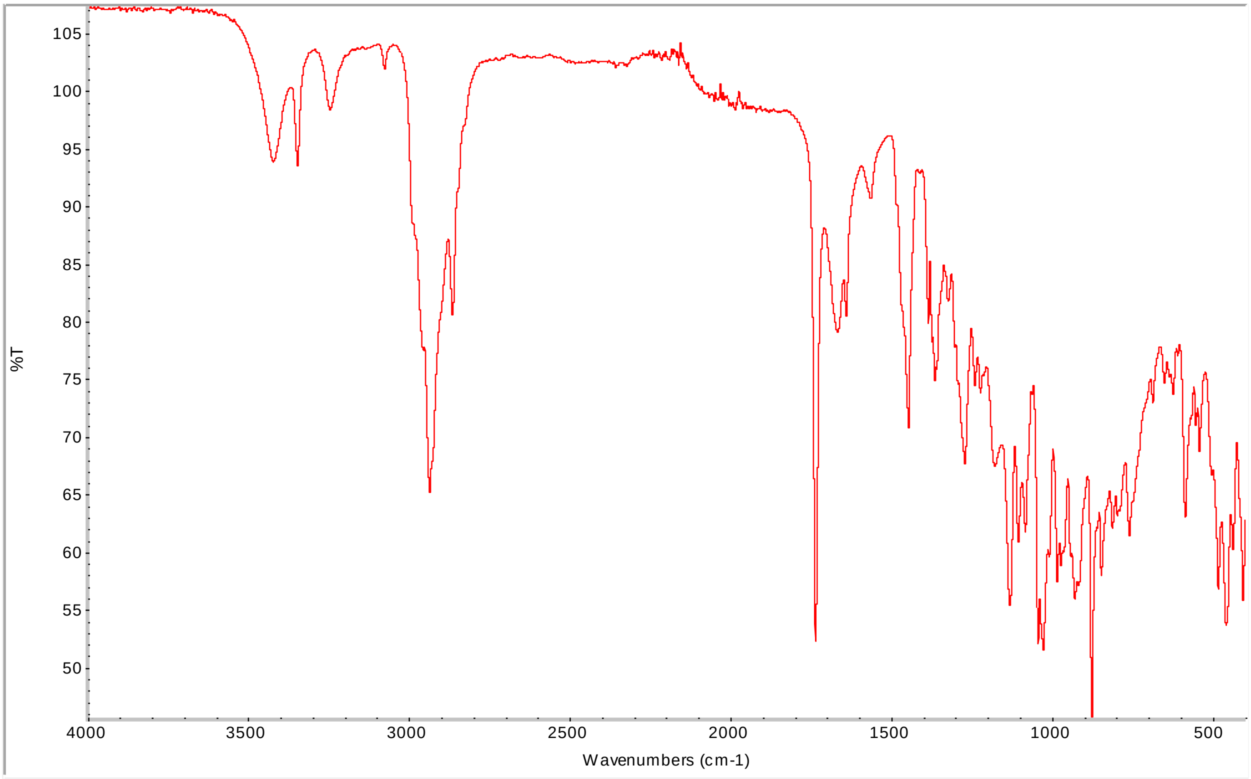

Figure S26. ATR-FTIR spectrum of [LeuOEt][BA].

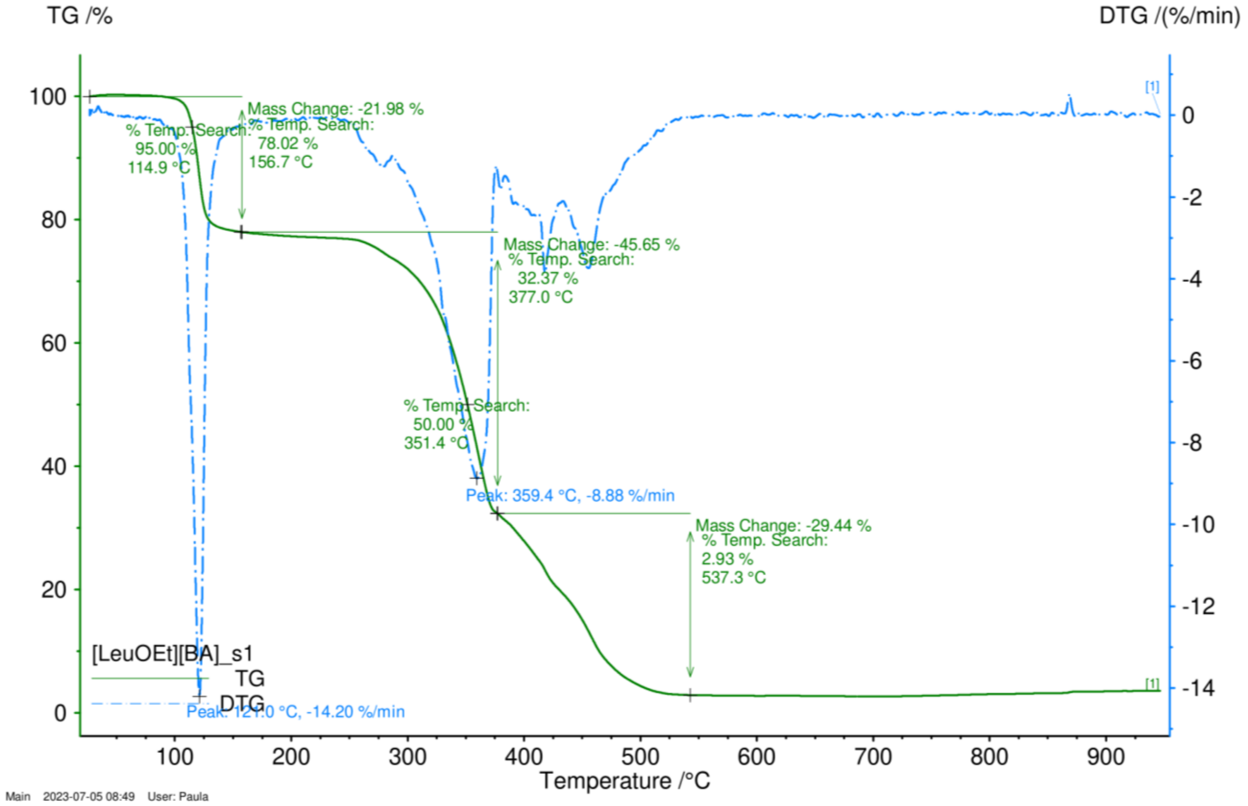

Figure S27. TG, DTG and c-DTA curves of [LeuOEt][BA].

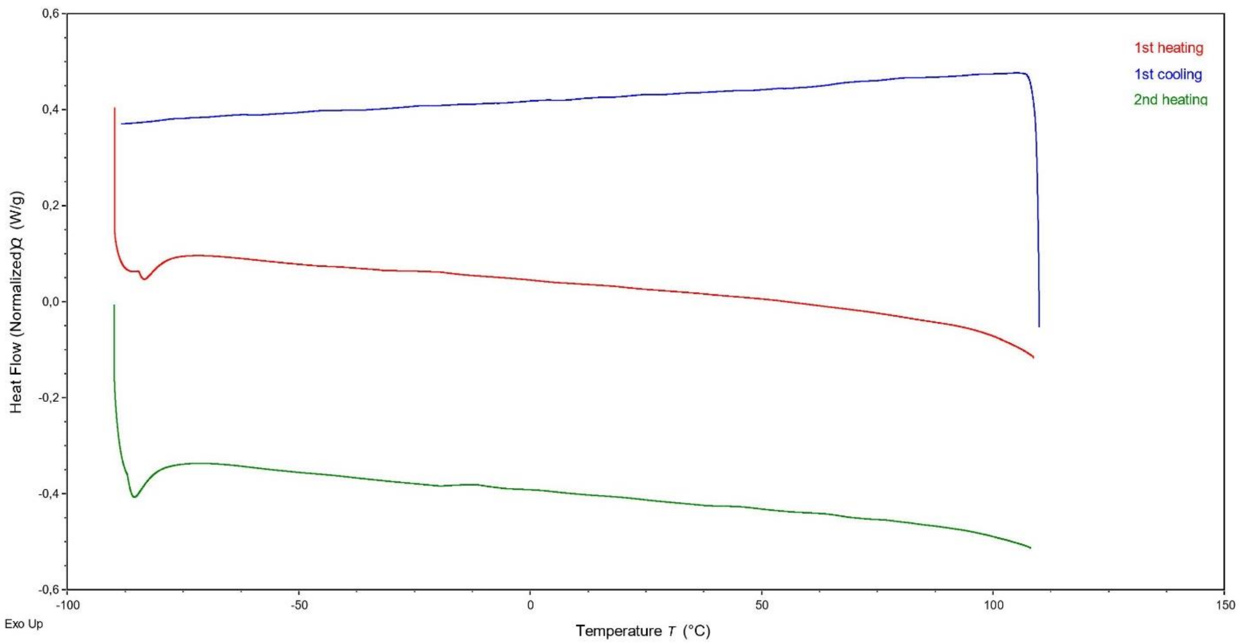

Figure S28. DSC curves of [LeuOEt][BA].

L-isoleucine ethyl ester betulinate - [IleOEt][BA]

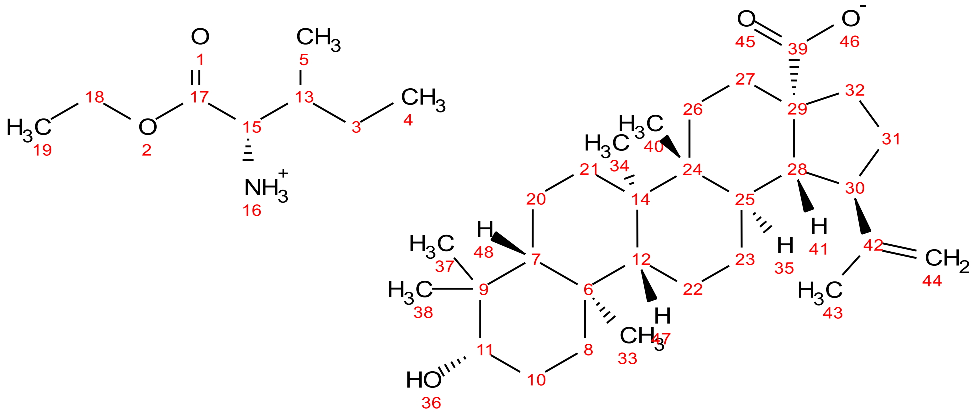

^{1}H NMR (400 MHz, DMSO-d_6) δ 4.68 (d, *J* = 2.5 Hz, 1H), 4.56 – 4.54 (m, 1H), 4.08 (qd, *J* = 7.1, 2.6 Hz, 2H), 3.38 (dd, *J* = 8.2, 4.9 Hz, 1H), 2.95 (td, *J* = 10.6, 5.8 Hz, 2H), 2.57 – 2.45 (m, 5H), 2.23 (t, *J* = 13.0 Hz, 1H), 2.12 (d, *J* = 10.7 Hz, 1H), 2.03 (s, 3H), 1.81 (dtd, *J* = 13.4, 7.7, 7.0, 4.2 Hz, 3H), 1.72 – 1.49 (m, 8H), 1.49 – 1.29 (m, 12H), 1.20 (q, *J* = 8.2, 7.1 Hz, 6H), 1.12 – 0.95 (m, 3H), 0.93 (s, 4H), 0.87 (s, 6H), 0.83 (s, 1H), 0.76 (s, 3H), 0.65 (s, 4H). ^{13}C NMR (100 MHz, DMSO-d_6) δ 177.75, 175.92, 150.80, 110.06, 99.99, 77.23, 60.51, 55.89, 55.36, 53.40, 50.40, 49.01, 47.08, 42.46, 40.71, 38.96, 38.73, 38.03, 37.18, 36.83, 34.37, 32.21, 30.58, 30.12, 29.68, 28.56, 27.63, 25.55, 20.92, 19.41, 18.44, 16.42, 16.27, 16.20, 15.04, 14.85, 14.60. FT–IR: ν (ATR): 3434, 3240, 3073, 2937, 2867, 2612, 1732, 1683, 1642, 1484, 1446, 1387, 1375, 1359, 1333, 1319, 1298, 1273, 1235, 1186, 1137, 1107, 1084, 1175, 1063, 1032, 1043, 1011, 984, 972, 945, 920, 881, 860, 792, 765, 749, 731, 701, 686, 651, 621, 608, 579, 556, 542, 525, 509, 482, 455, 438. Elemental analysis: calc. (%) for $C_{38}H_{65}NO_5$ (615.940 g/mol): C (74.10), H (10.64), N (2.27), O (12.99); found: C (74.11), H (10.64), N (2.27), O (13.00).

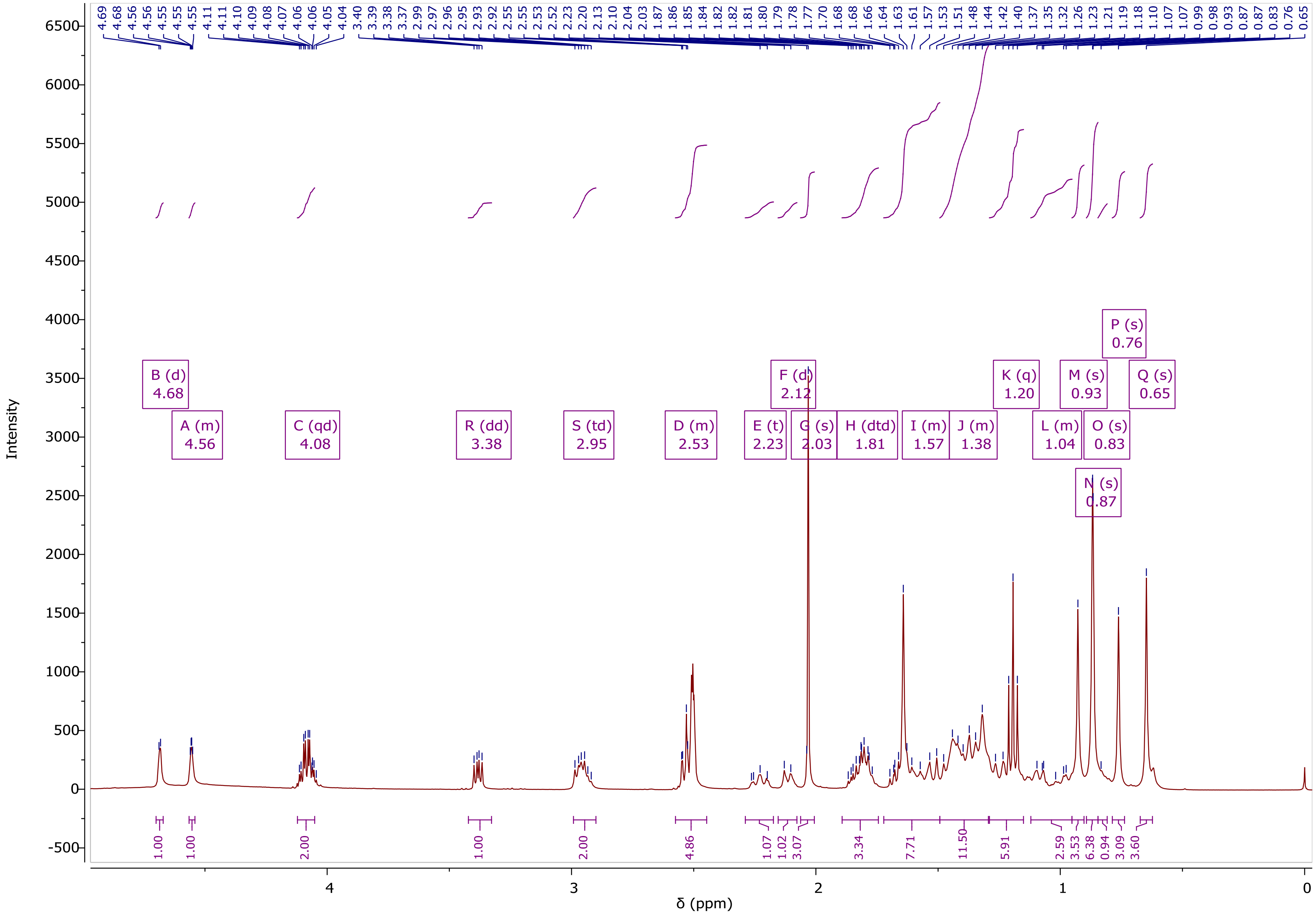

Figure S29. ^{1}H NMR spectrum of [IleOEt][BA].

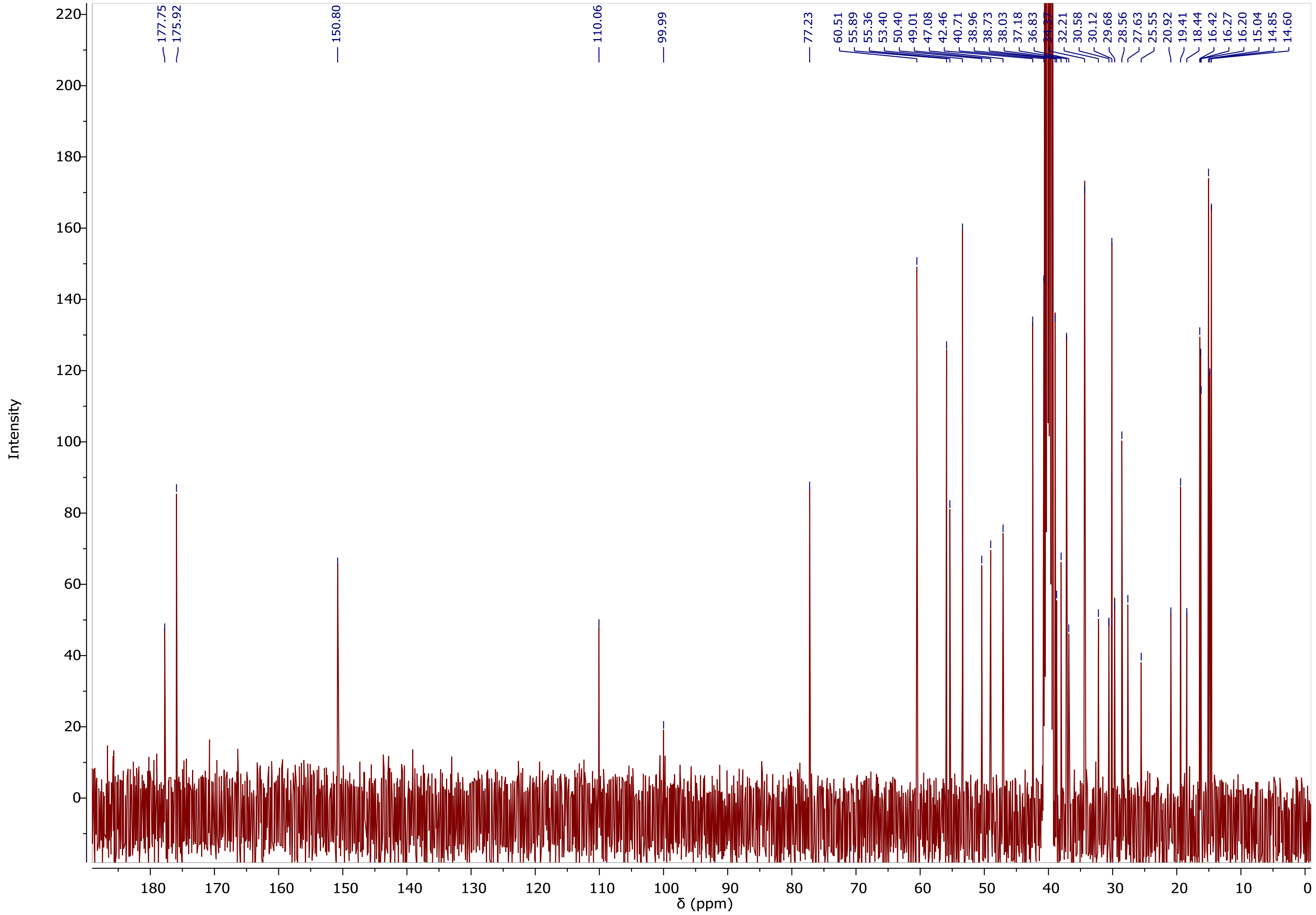

Figure S30. ^{13}C NMR spectrum of [IleOEt][BA].

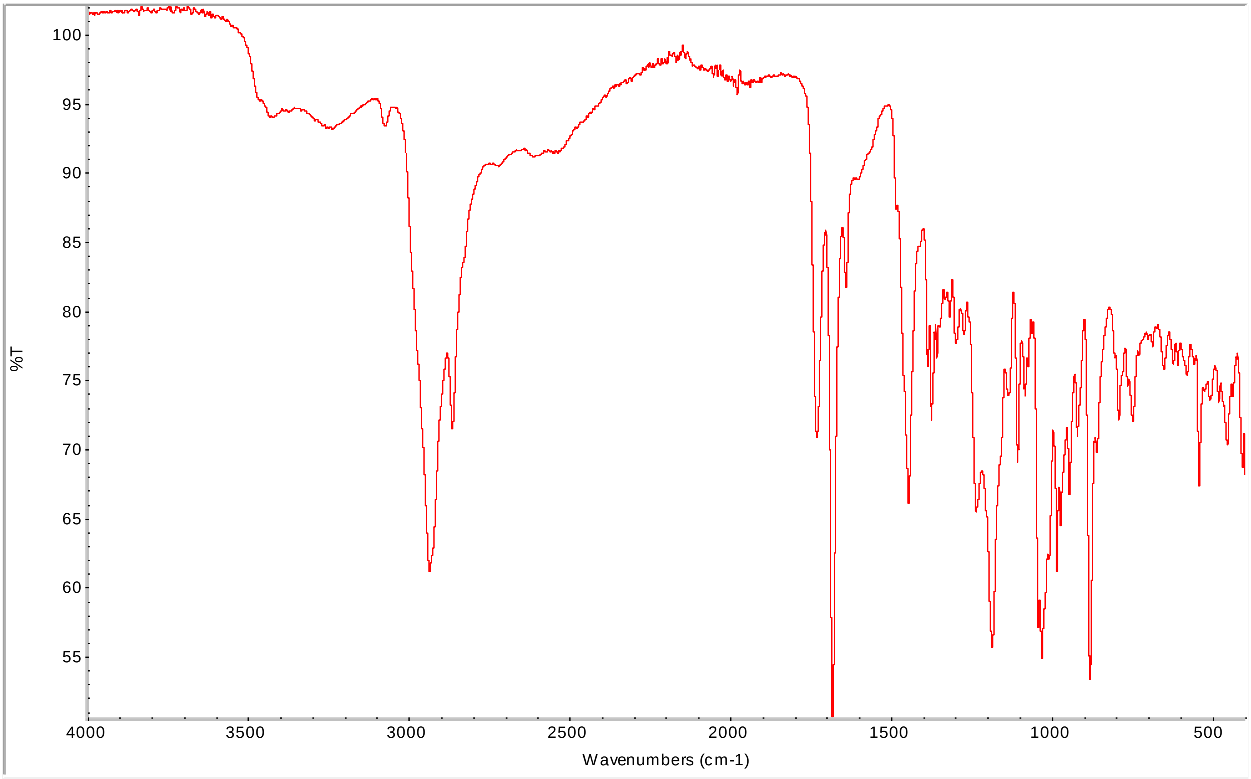

Figure S31. ATR-FTIR spectrum of [IleOEt][BA].

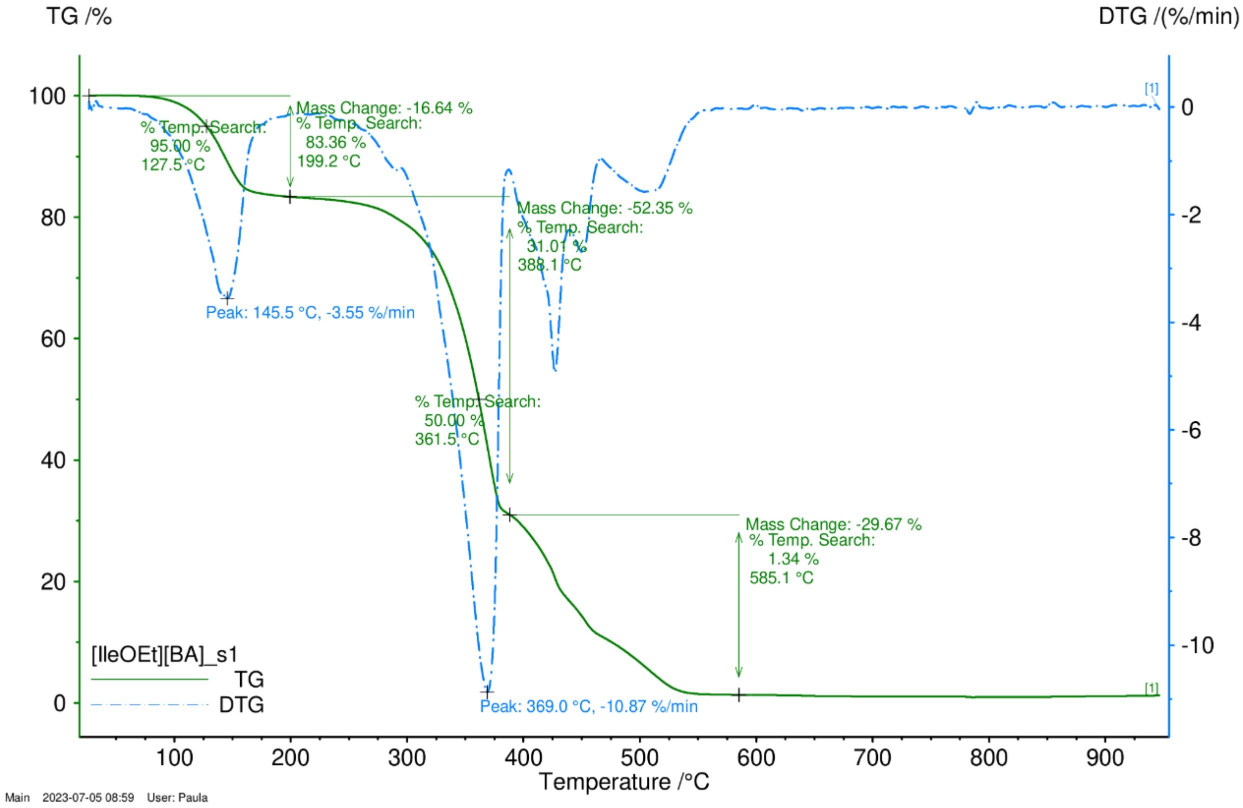

Figure S32. TG, DTG and c-DTA curves of [IleOEt][BA].

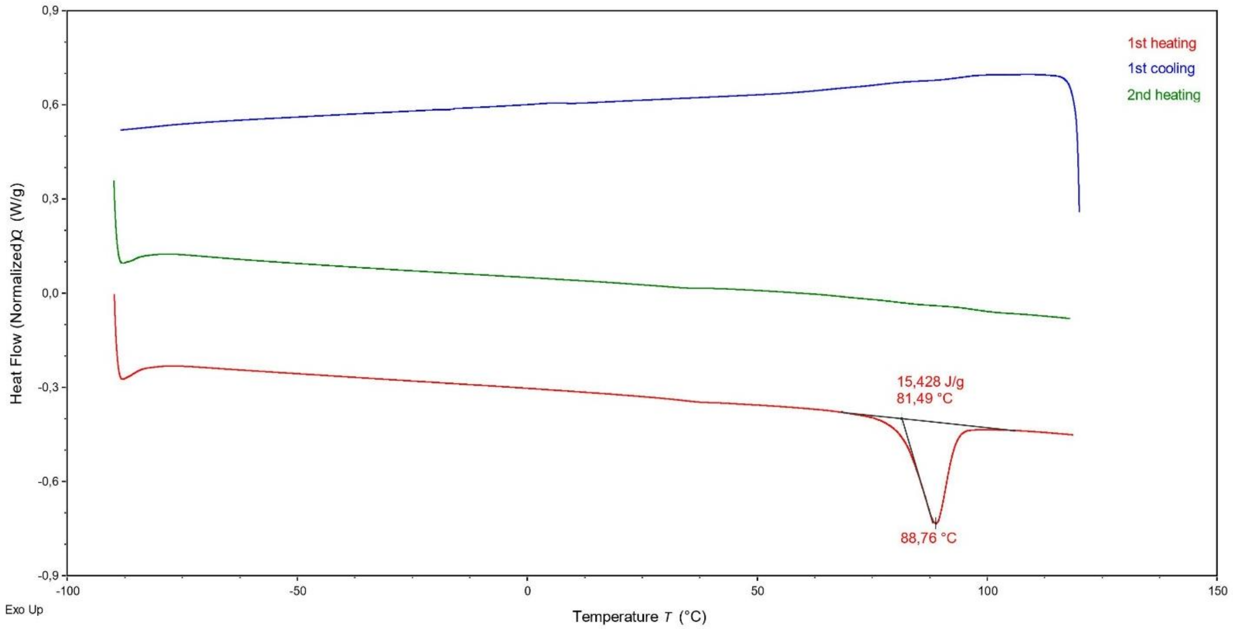

Figure S33. DSC curves of [IleOEt][BA].

L-threonine ethyl ester betulinate - [ThrOEt][BA]

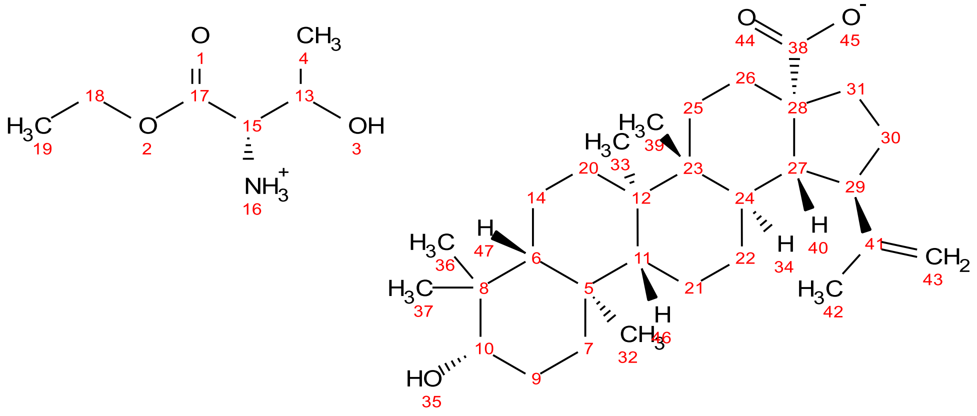

^{1}H NMR (400 MHz, DMSO-d_6) δ 4.68 (d, J = 2.5 Hz, 1H), 4.57 – 4.54 (m, 1H), 4.08 (q, J = 7.1 Hz, 2H), 3.83 (qd, J = 6.3, 4.0 Hz, 1H), 3.13 (d, J = 4.0 Hz, 1H), 2.95 (td, J = 10.2, 5.8 Hz, 2H), 2.23 (td, J = 12.4, 11.6, 3.3 Hz, 1H), 2.16 – 2.08 (m, 1H), 1.79 (q, J = 7.2, 6.0 Hz, 2H), 1.64 (s, 4H), 1.59 – 1.47 (m, 3H), 1.39 (dd, J = 27.8, 10.3 Hz, 8H), 1.33 – 1.27 (m, 4H), 1.19 (t, J = 7.1 Hz, 6H), 1.09 (d, J = 6.4 Hz, 5H), 0.93 (s, 4H), 0.89 – 0.81 (m, 7H), 0.76 (s, 3H), 0.65 (s, 4H). ^{13}C NMR (100 MHz, DMSO-d_6) δ 177.79, 174.69, 150.80, 110.06, 77.23, 68.35, 60.45, 60.39, 55.89, 55.37, 50.41, 49.01, 47.08, 42.45, 40.71, 38.96, 38.74, 38.03, 37.18, 36.85, 34.39, 32.23, 30.59, 29.68, 28.55, 27.63, 25.55, 20.93, 20.62, 19.41, 18.44, 16.42, 16.26, 16.20, 14.84, 14.60. FT–IR: ν (ATR): 3427, 3363, 3079, 2938, 2867, 1722, 1683, 1642, 1586, 1447, 1386, 1375, 1359, 1328, 1302, 1273, 1212, 1186, 1140, 1107, 1074, 1042, 1028, 982, 973, 937, 911, 881, 793, 766, 749, 685, 668, 651, 622, 608, 578, 558, 542, 526, 505, 453, 435. Elemental analysis: calc. (%) for $C_{36}H_{61}NO_6$ (603.885 g/mol): C (71.60), H (10.18), N (2.32), O (19.90); found: C (71.61), H (10.18), N (2.33), O (19.91).

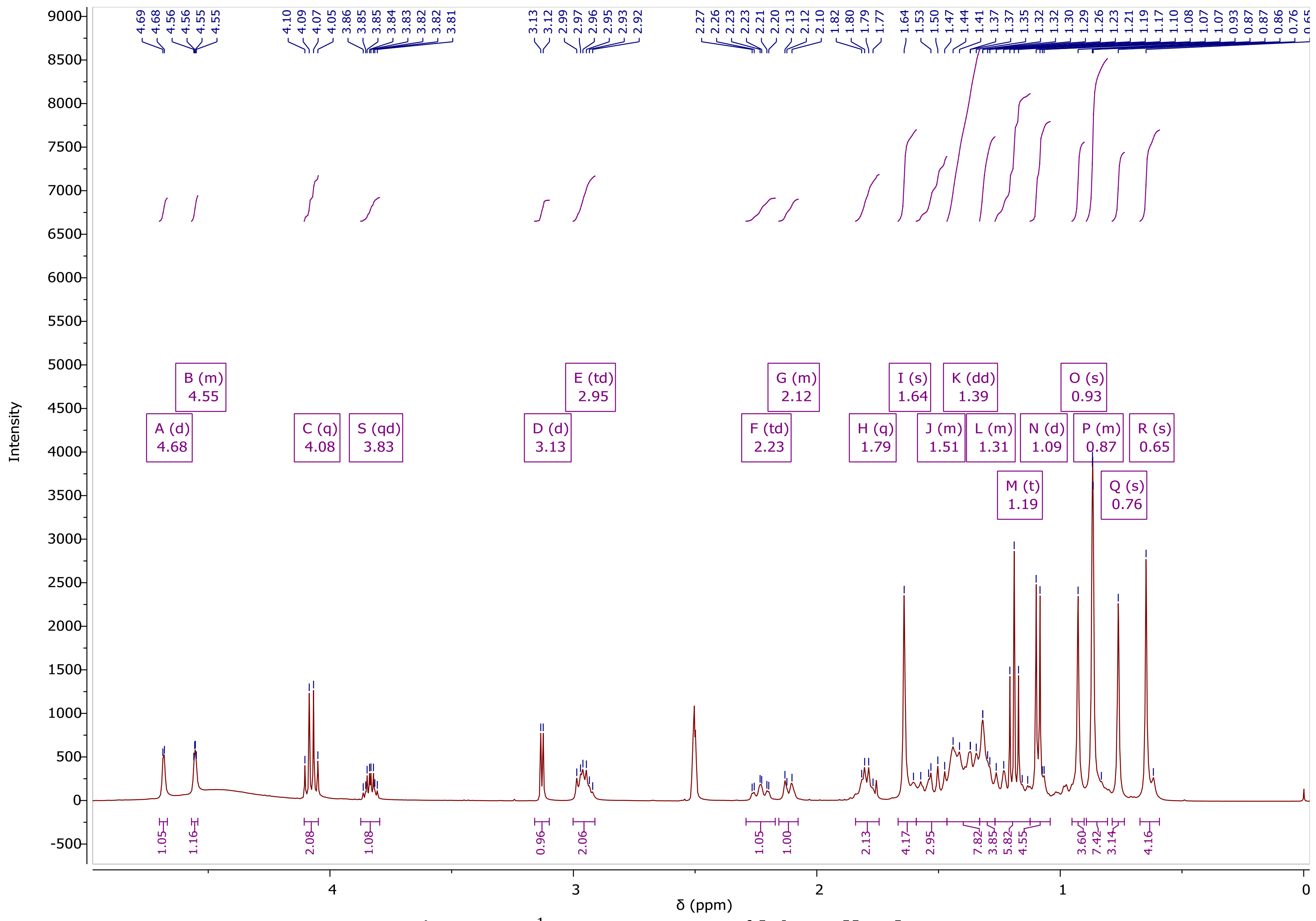

Figure S34. ^{1}H NMR spectrum of [ThrOEt][BA].

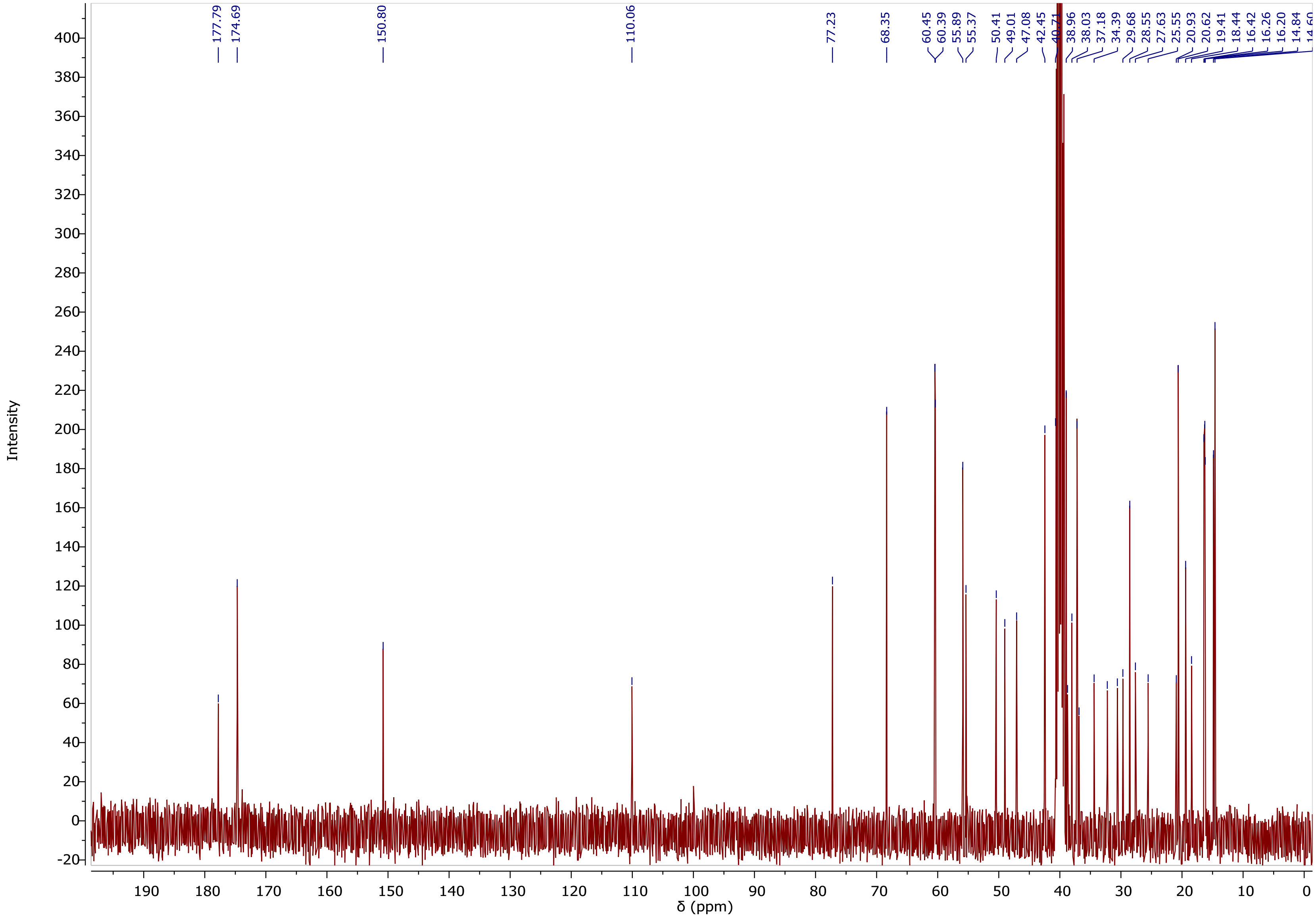

Figure S35. ^{13}C NMR spectrum of [ThrOEt][BA].

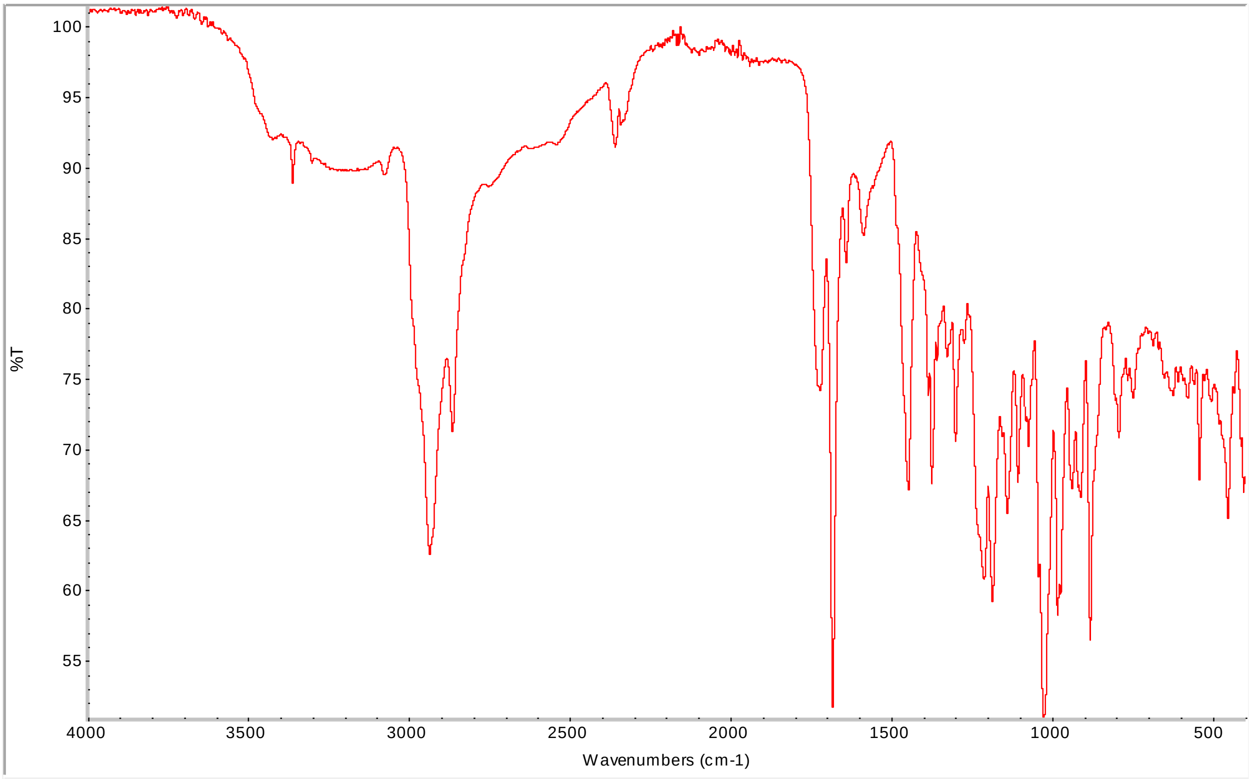

Figure S36. ATR-FTIR spectrum of [ThrOEt][BA].

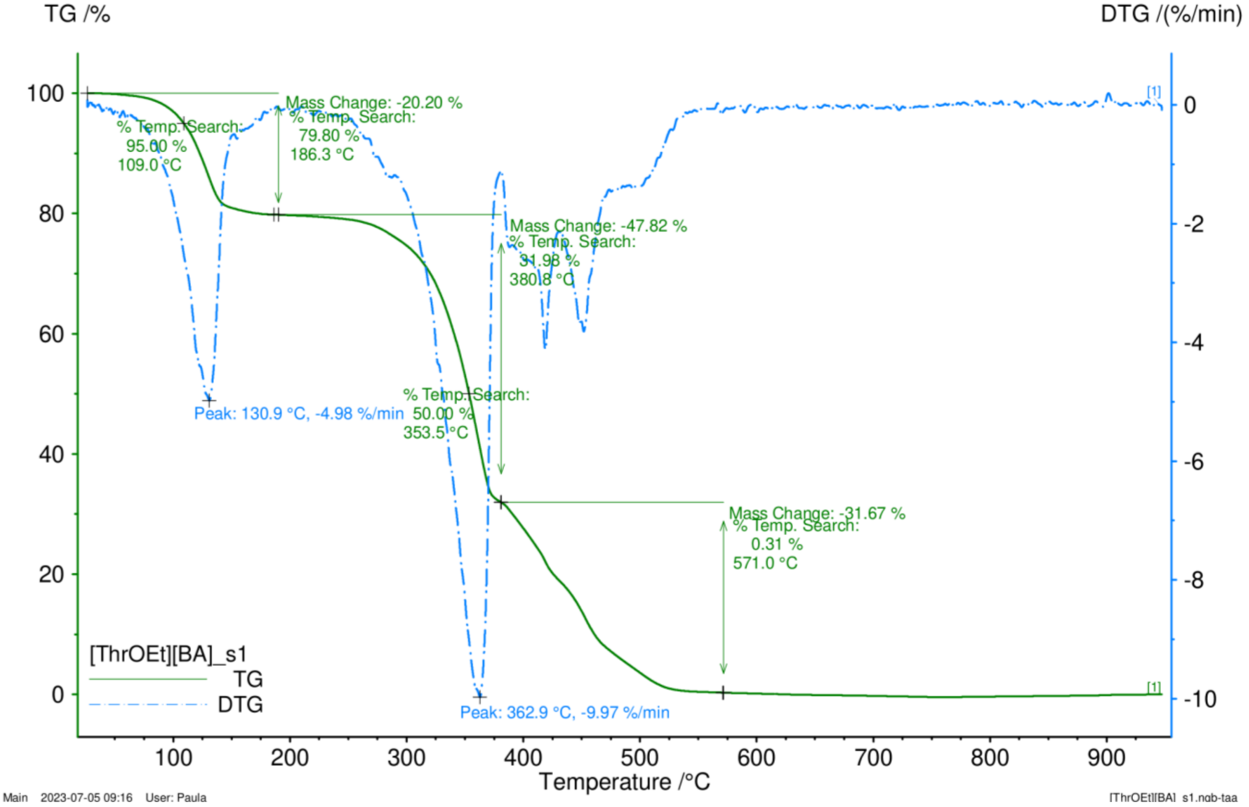

Figure S37. TG, DTG and c-DTA curves of [ThrOEt][BA].

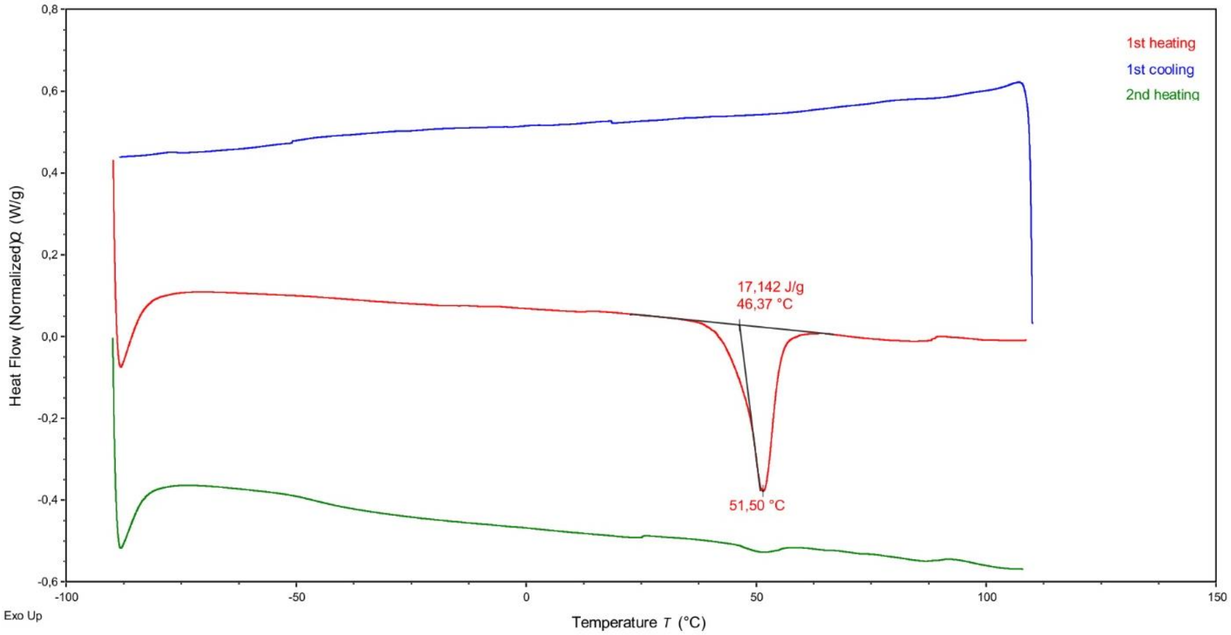

Figure S38. DSC curves of [ThrOEt][BA].

L-serine ethyl ester betulinate - [SerOEt][BA]

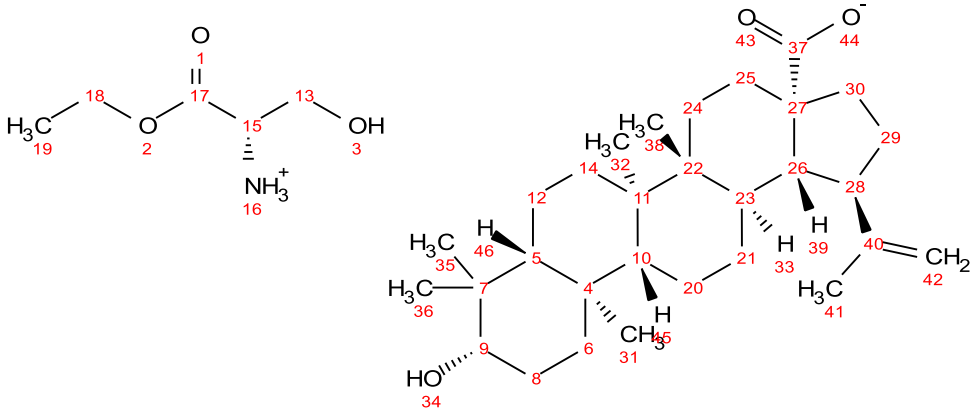

^{1}H NMR (400 MHz, DMSO-d_6) δ 4.68 (d, *J* = 2.5 Hz, 1H), 4.56 – 4.54 (m, 1H), 4.10 – 4.04 (m, 2H), 3.56 – 3.50 (m, 2H), 3.35 (t, *J* = 4.8 Hz, 1H), 3.02 – 2.89 (m, 2H), 2.24 (td, *J* = 12.3, 3.5 Hz, 1H), 2.16 – 2.05 (m, 1H), 1.79 (q, *J* = 7.4, 5.6 Hz, 2H), 1.64 (s, 3H), 1.59 – 1.43 (m, 5H), 1.43 – 1.27 (m, 9H), 1.27 – 1.20 (m, 3H), 1.20 – 1.03 (m, 5H), 0.93 (s, 3H), 0.88 – 0.81 (m, 6H), 0.76 (s, 3H), 0.65 (s, 3H). ^{13}C NMR (100 MHz, DMSO-d_6) δ 177.86, 174.54, 150.82, 110.03, 77.23, 64.47, 60.43, 56.78, 55.91, 55.37, 50.42, 49.02, 47.08, 42.45, 40.71, 38.95, 38.74, 38.02, 37.18, 36.88, 34.40, 32.27, 30.60, 29.69, 28.55, 27.63, 25.55, 20.94, 19.41, 18.44, 16.41, 16.26, 16.20, 14.84, 14.60. FT–IR: ν (ATR): 3366, 3248, 3080, 2937, 2867, 1738, 1684, 1642, 1558, 1446, 1386, 1375, 1360, 1332, 1298, 1273, 1228, 1210, 1194, 1136, 1106, 1083, 1071, 1043, 1032, 983, 972, 944, 920, 881, 862, 837, 793, 765, 748, 703, 686, 649, 621, 575, 558, 542, 526, 509, 478, 457, 445, 418. Elemental analysis: calc. (%) for $C_{35}H_{59}NO_6$ (589.858 g/mol): C (71.27), H (10.08), N (2.37), O (16.27); found: C (71.28), H (10.09), N (2.37), O (16.26).

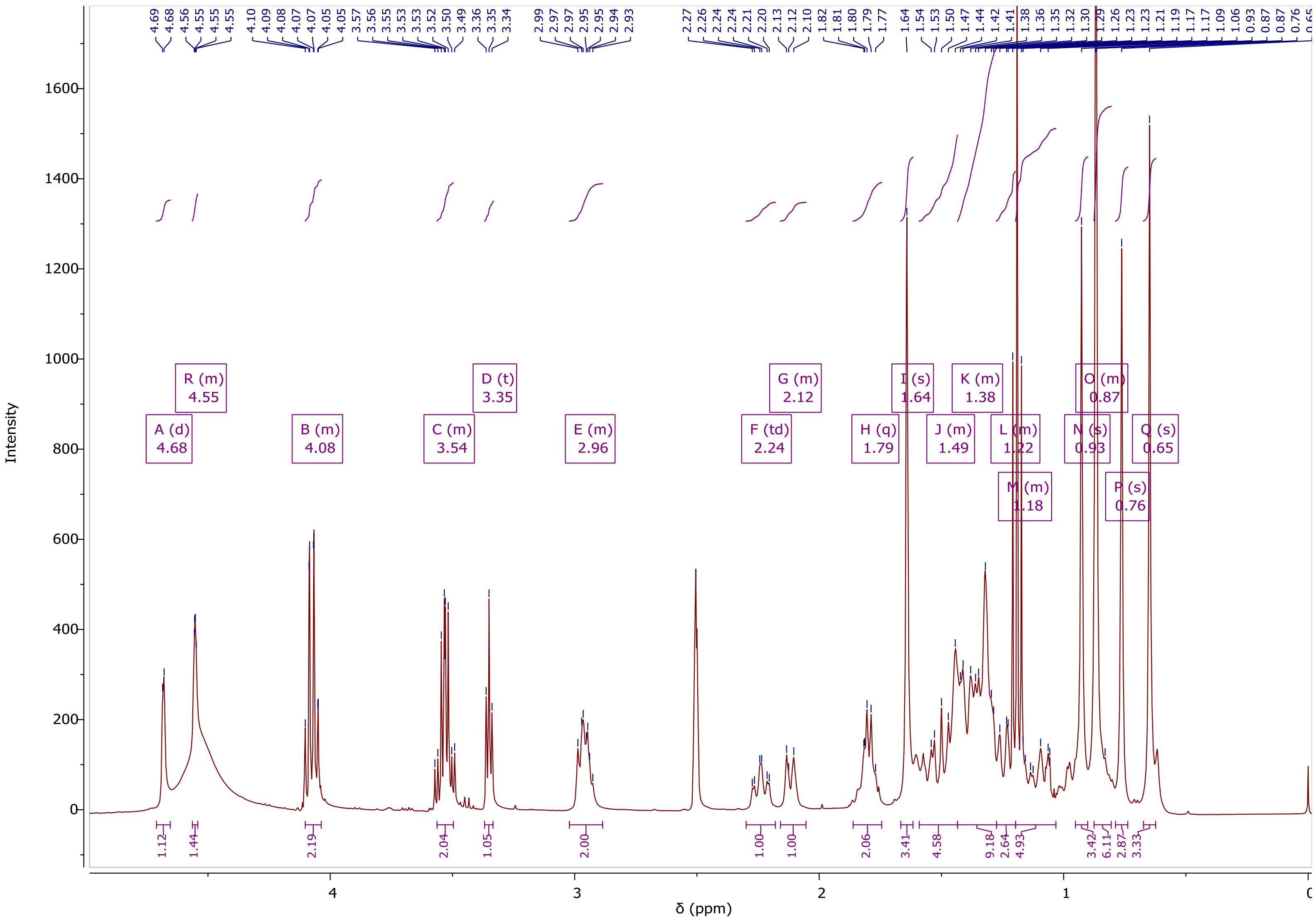

Figure S39. ^{1}H NMR spectrum of [SerOEt][BA].

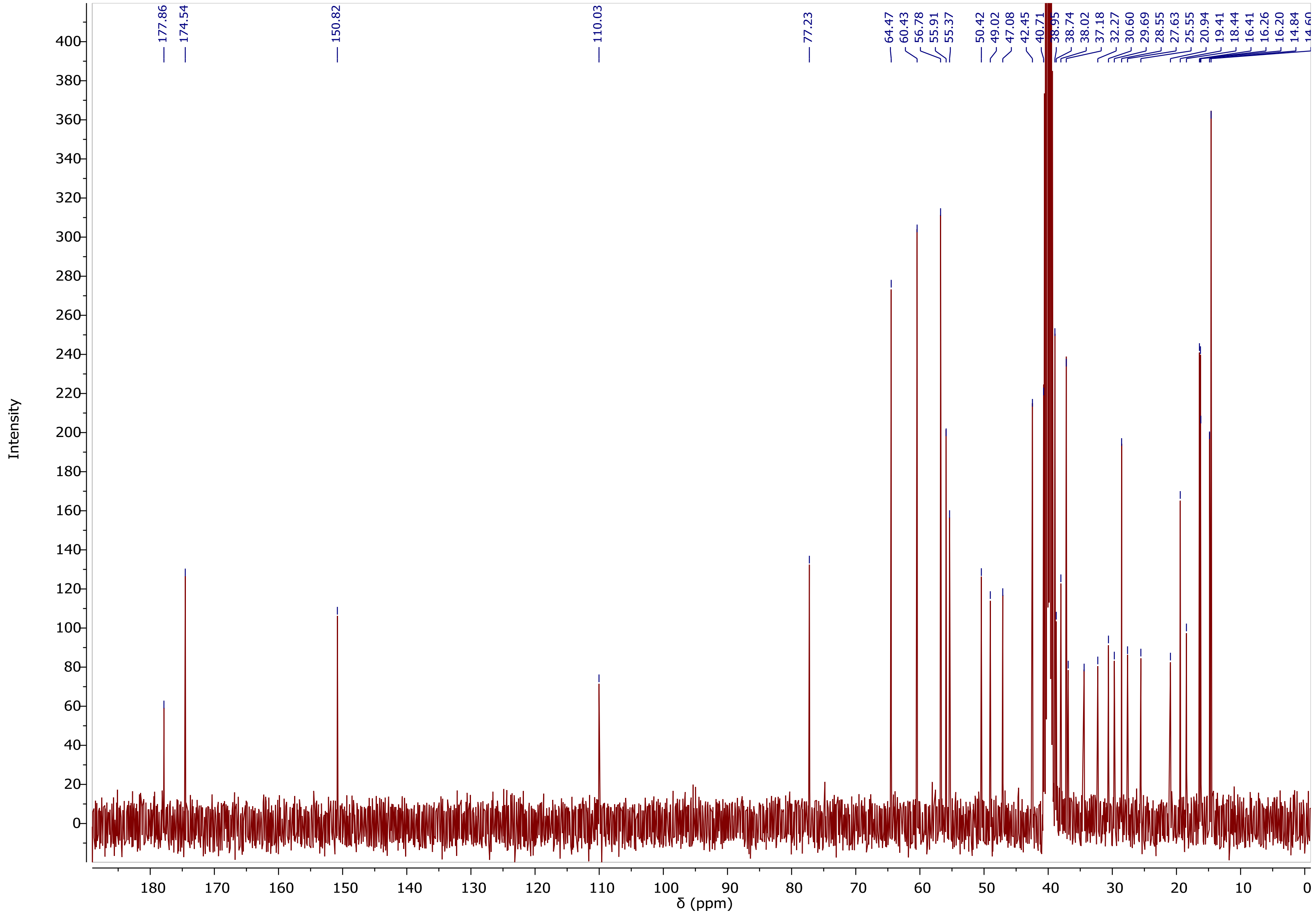

Figure S40. ^{13}C NMR spectrum of [SerOEt][BA].

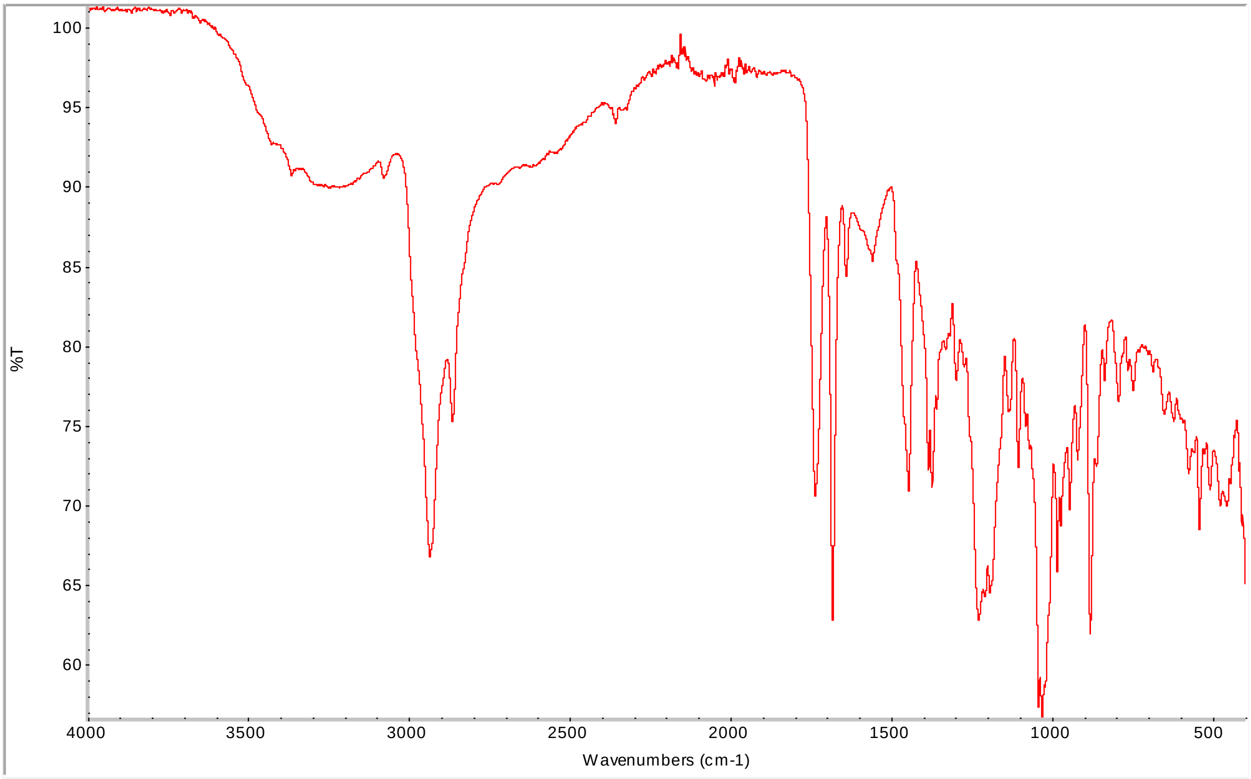

Figure S41. ATR-FTIR spectrum of [SerOEt][BA].

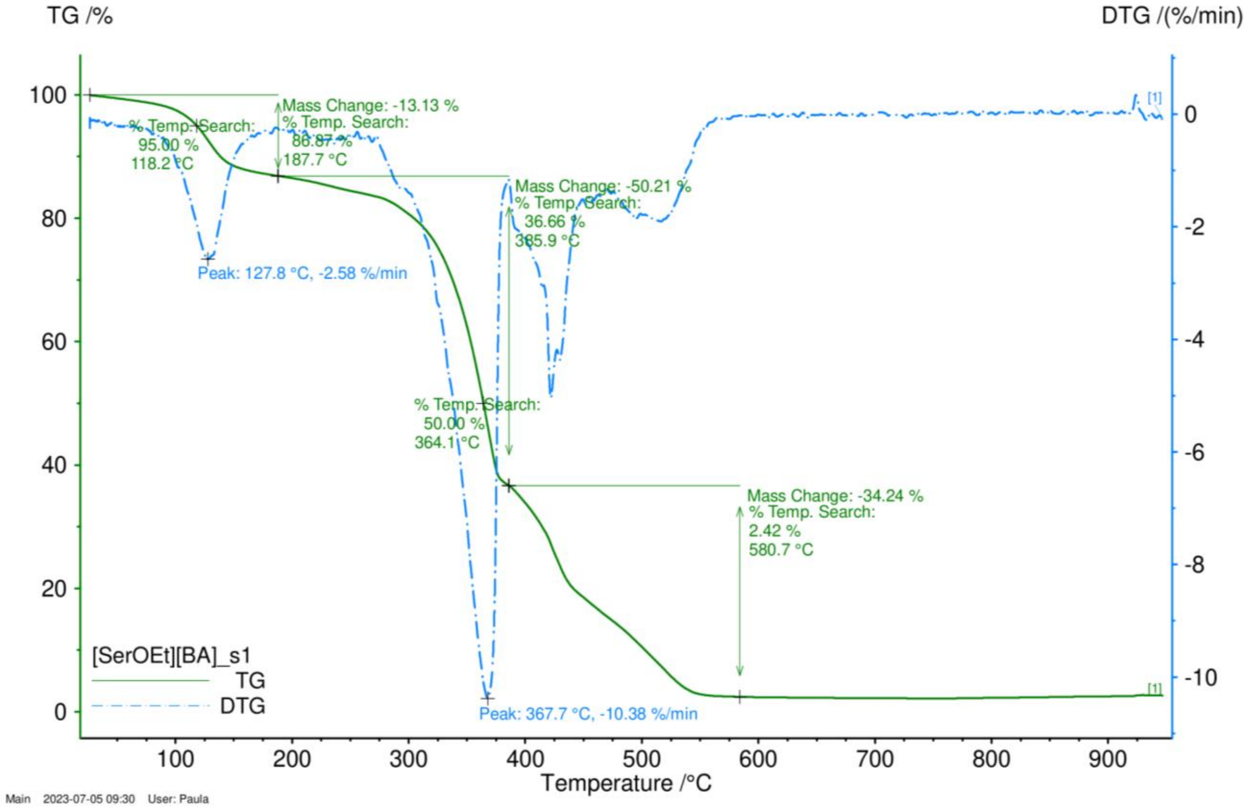

Figure S42. TG, DTG and c-DTA curves of [SerOEt][BA].

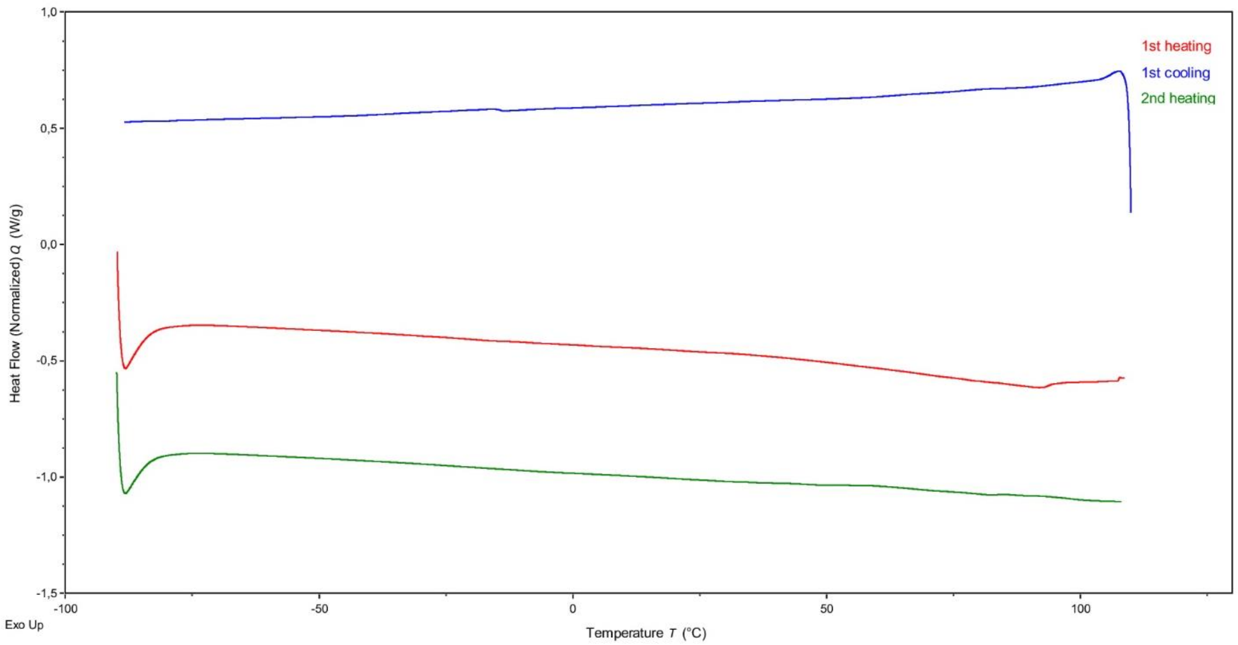

Figure S43. DSC curves of [SerOEt][BA].

L-cysteine ethyl ester betulinate - [CysOEt][BA]

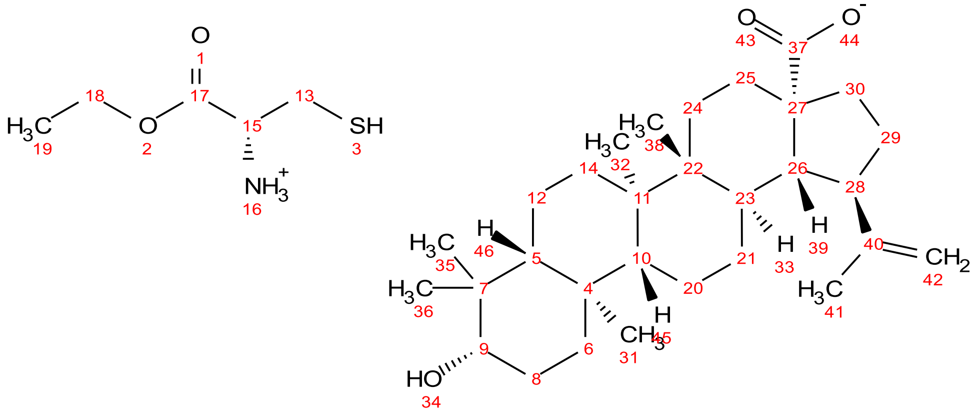

^{1}H NMR (400 MHz, DMSO-d_6) δ 4.69 (d, J = 2.5 Hz, 1H), 4.59 – 4.53 (m, 1H), 4.10 (q, J = 7.1 Hz, 2H), 3.59 (t, J = 6.4 Hz, 1H), 2.98 – 2.90 (m, 3H), 2.22 (td, J = 12.4, 3.5 Hz, 1H), 2.15 – 2.07 (m, 1H), 1.84 – 1.74 (m, 2H), 1.64 (s, 3H), 1.56 (dd, J = 25.8, 11.9 Hz, 3H), 1.49 – 1.38 (m, 5H), 1.38 – 1.24 (m, 7H), 1.21 (t, J = 7.1 Hz, 4H), 1.17 – 1.03 (m, 2H), 0.93 (s, 3H), 0.87 (d, J = 1.6 Hz, 6H), 0.76 (s, 3H), 0.65 (s, 4H). ^{13}C NMR (100 MHz, DMSO-d_6) δ 177.71, 174.18, 150.77, 110.09, 77.23, 60.83, 55.88, 55.36, 54.20, 50.40, 48.99, 47.07, 44.03, 42.46, 40.71, 38.96, 38.73, 38.04, 37.18, 36.81, 34.38, 32.18, 30.57, 29.67, 28.56, 27.62, 25.54, 20.92, 19.40, 18.43, 16.42, 16.27, 16.19, 14.85, 14.53. FT–IR: ν (ATR): 3436, 3376, 3250, 3073, 2987, 2938, 2867, 2721, 2604, 1733, 1683, 1642, 1593, 1558, 1484, 1446, 1411, 1387, 1375, 1359, 1350, 1318, 1301, 1272, 1235, 1186, 1137, 1107, 1085, 1075, 1062, 1042, 1030, 983, 972, 944, 920, 881, 860, 803, 792, 766, 749, 730, 702, 651, 621, 608, 576, 556, 542, 507, 482, 473, 453, 438, 429. Elemental analysis: calc. (%) for $C_{35}H_{59}NO_5S$ (605.919 g/mol): C (69.38), H (9.82), N (2.31), O (13.20), S (5.29); found: C (69.40), H (9.80), N (2.32), O (13.21), S (5.28).

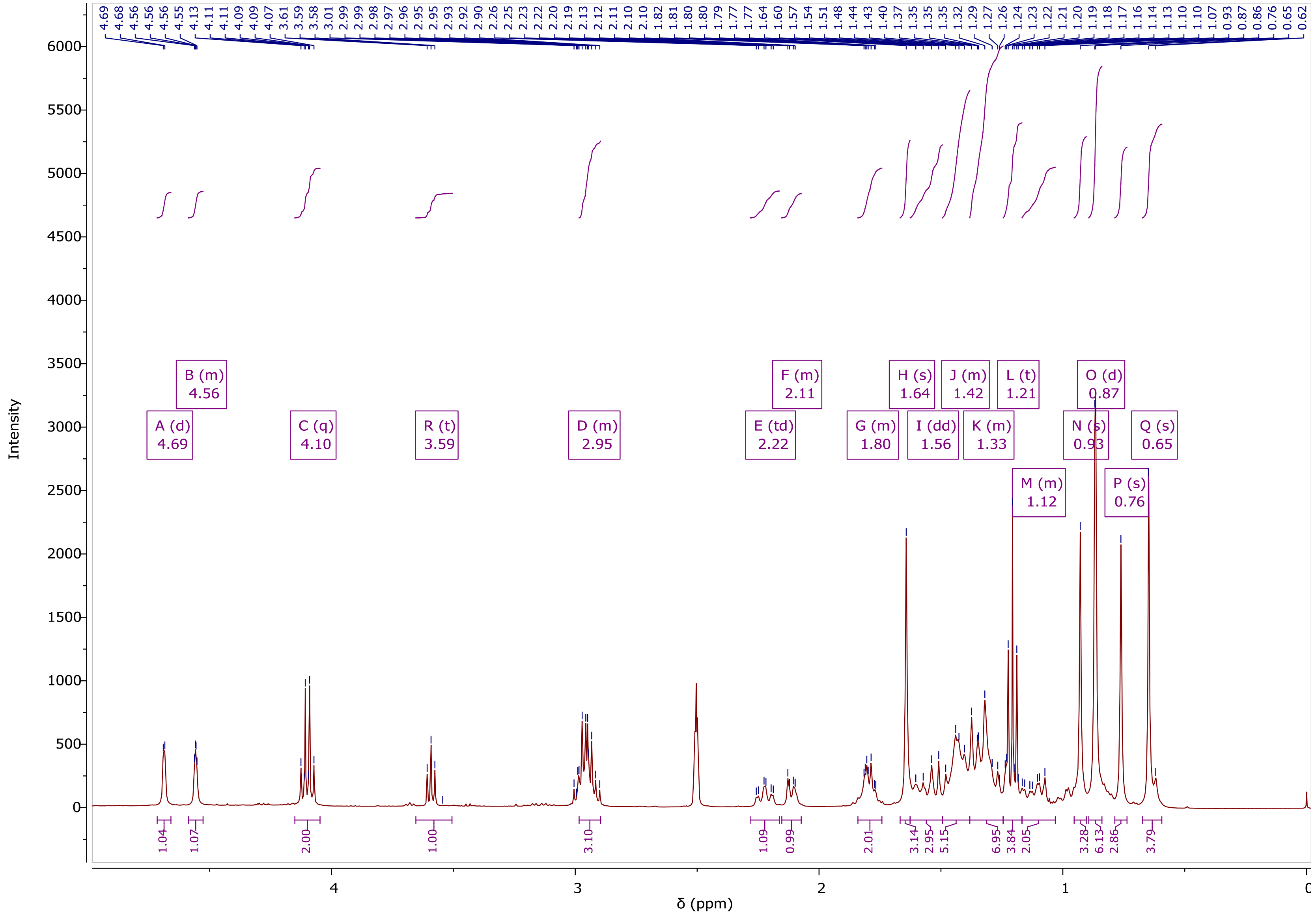

Figure S44. ^{1}H NMR spectrum of [CysOEt][BA].

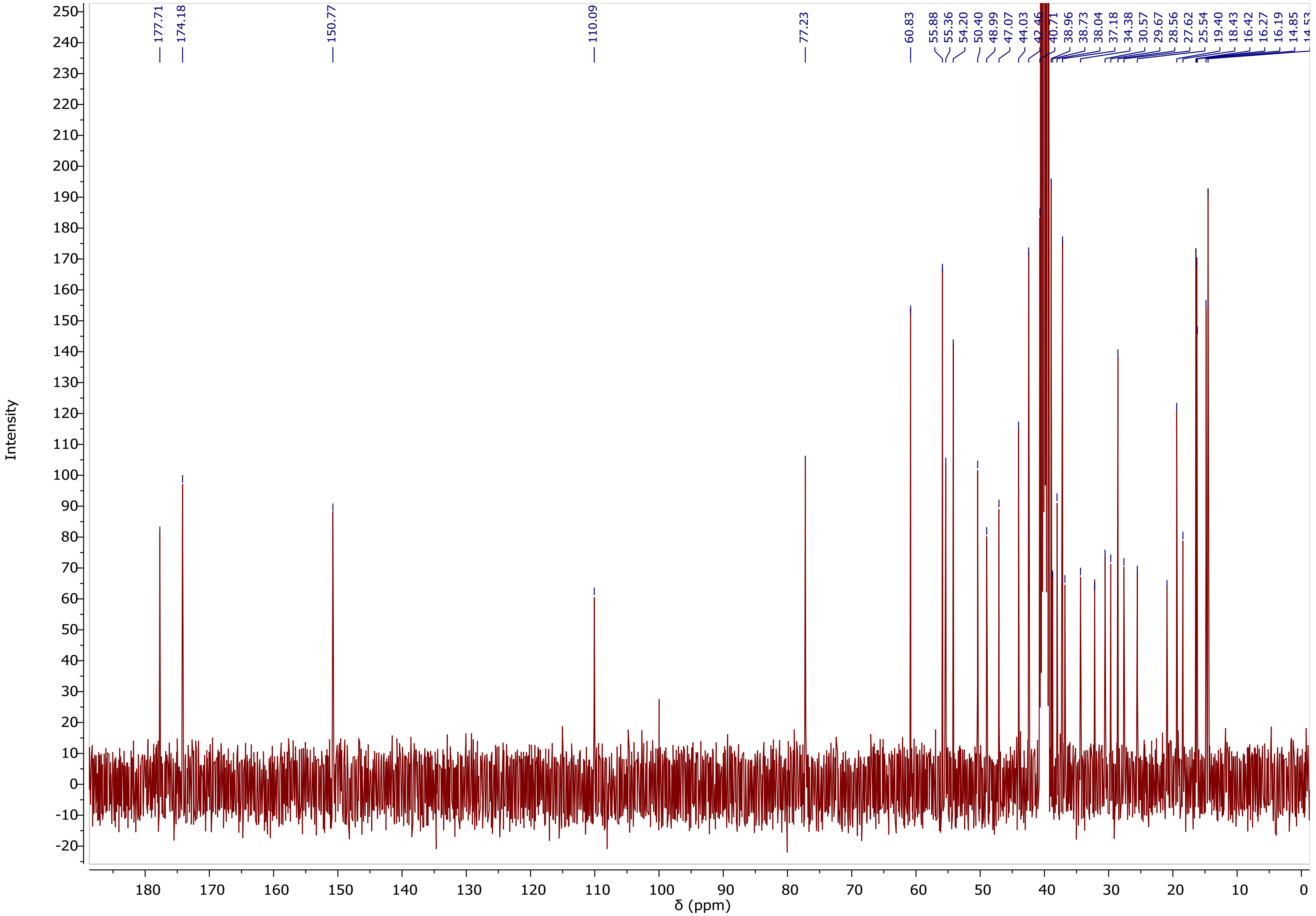

Figure S45. ^{13}C NMR spectrum of [CysOEt][BA].

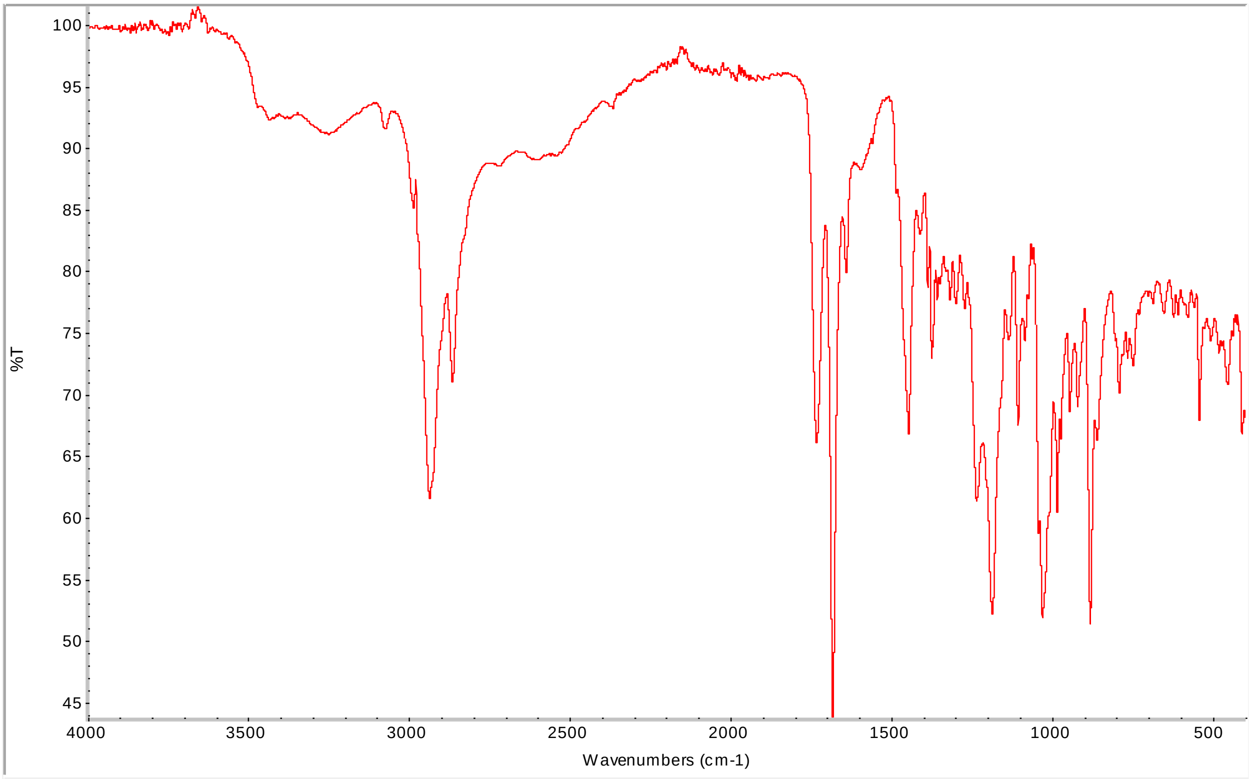

Figure S46. ATR-FTIR spectrum of [CysOEt][BA].

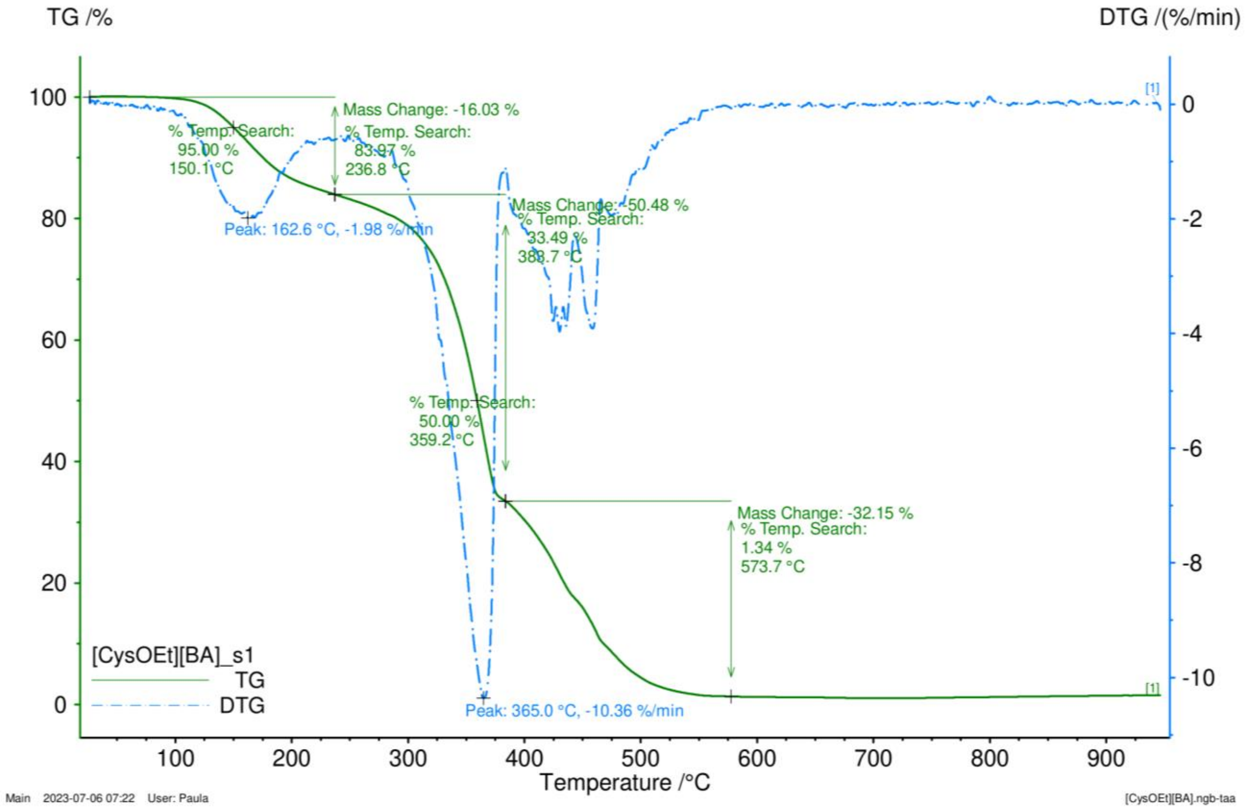

Figure S47. TG, DTG and c-DTA curves of [CysOEt][BA].

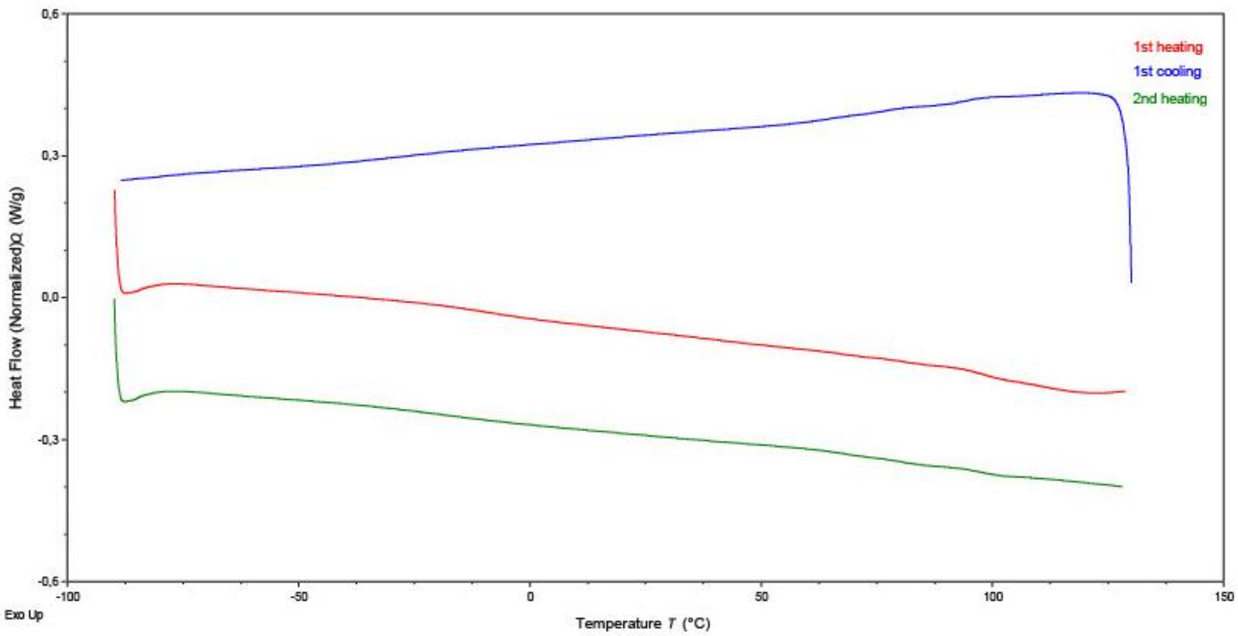

Figure S48. DSC curves of [CysOEt][BA].

L-methionine ethyl ester betulinate - [MetOEt][BA]

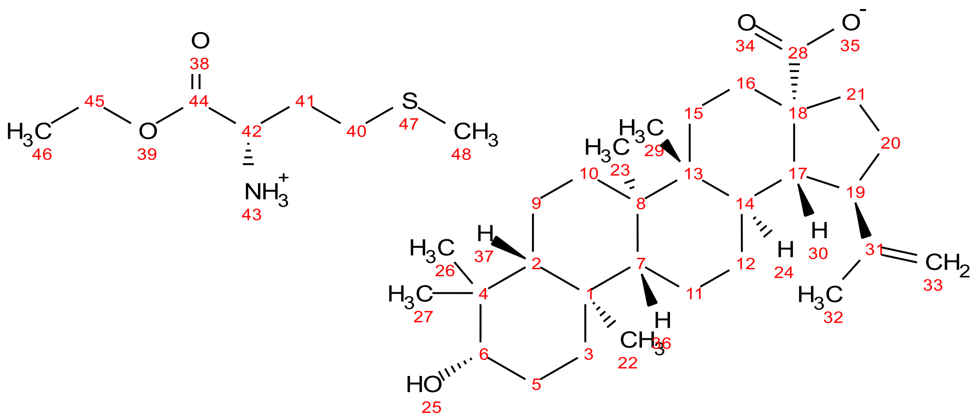

^{1}H NMR (400 MHz, DMSO-d_6) δ 4.68 (d, *J* = 2.5 Hz, 1H), 4.57 – 4.54 (m, 1H), 4.08 (qd, *J* = 7.1, 2.6 Hz, 2H), 3.38 (dd, *J* = 8.2, 4.9 Hz, 1H), 2.95 (td, *J* = 10.4, 5.8 Hz, 2H), 2.55 – 2.48 (m, 4H), 2.23 (t, *J* = 11.1 Hz, 1H), 2.12 (d, *J* = 10.8 Hz, 1H), 2.03 (s, 3H), 1.88 – 1.72 (m, 3H), 1.64 (s, 4H), 1.62 – 1.45 (m, 4H), 1.45 – 1.13 (m, 15H), 1.12 – 0.93 (m, 3H), 0.93 (s, 2H), 0.89 – 0.81 (m, 7H), 0.76 (s, 3H), 0.65 (s, 4H). ^{13}C NMR (100 MHz, DMSO-d_6) δ 177.76, 175.92, 150.81, 110.07, 77.23, 60.51, 55.89, 55.36, 53.40, 50.40, 49.01, 47.08, 42.46, 40.71, 38.96, 38.73, 38.03, 37.19, 36.84, 34.37, 32.23, 30.58, 30.12, 29.68, 28.56, 27.63, 25.55, 20.93, 19.41, 18.44, 16.42, 16.27, 16.20, 15.04, 14.85, 14.60. FT–IR: ν (ATR): 3437, 3246, 3079, 2937, 2867, 1732, 1683, 1642, 1484, 1446, 1387, 1375, 1359, 1333, 1319, 1300, 1272, 1259, 1234, 1185, 1136, 1107, 1084, 1074, 1062, 1043, 1031, 1010, 983, 972, 944, 920, 881, 859, 792, 765, 749, 701, 685, 669, 651, 621, 608, 581, 556, 542, 526, 506, 482, 454, 433. Elemental analysis: calc. (%) for $C_{37}H_{63}NO_5S$ (633.973 g/mol): C (70.10%), H (10.02%), N (2.21%), O (12.62%), S (5.06%); found: C (70.11%), H (10.02%), N (2.20%), O (12.62%), S (5.05%).

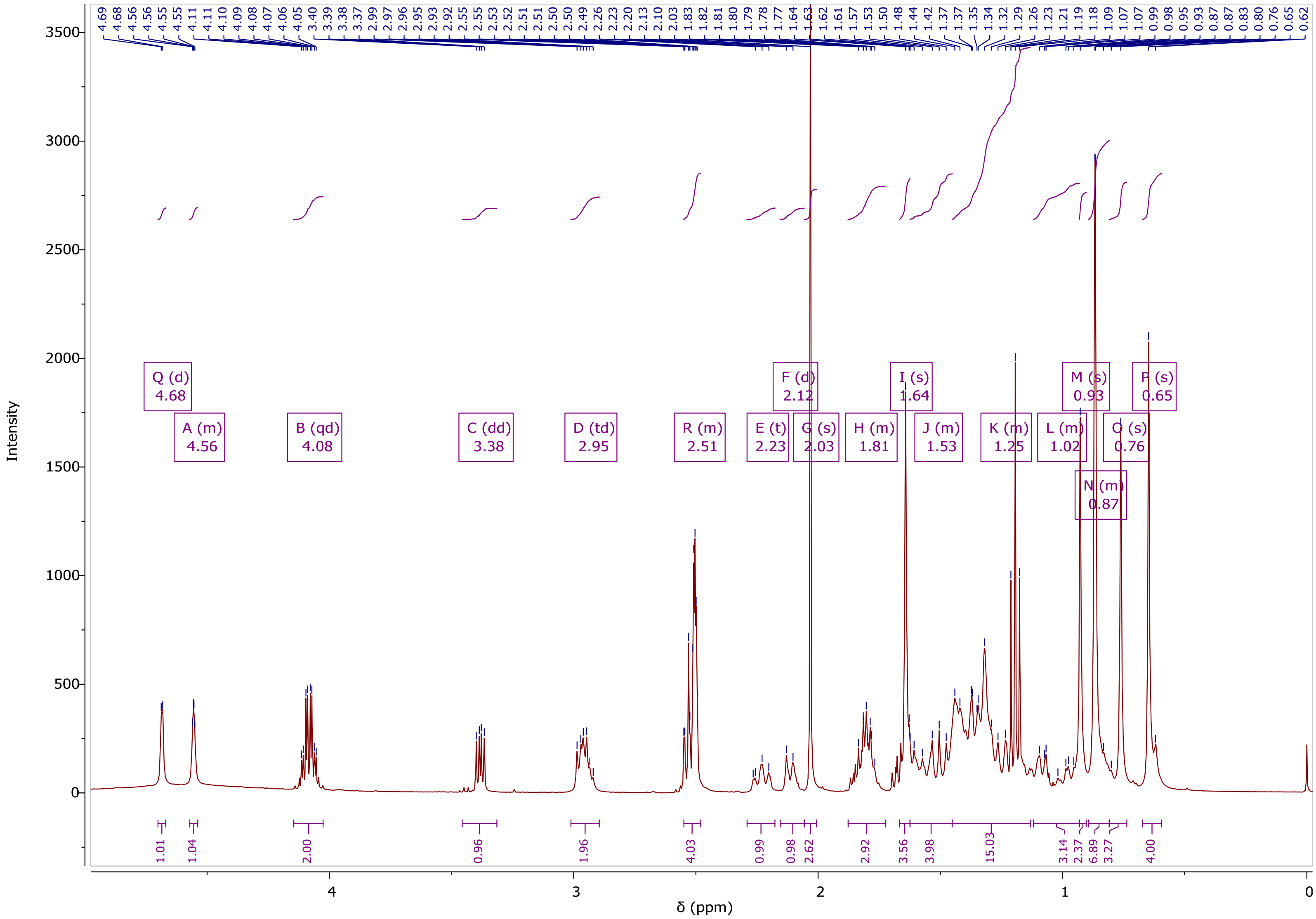

Figure S49. ^{1}H NMR spectrum of [MetOEt][BA].

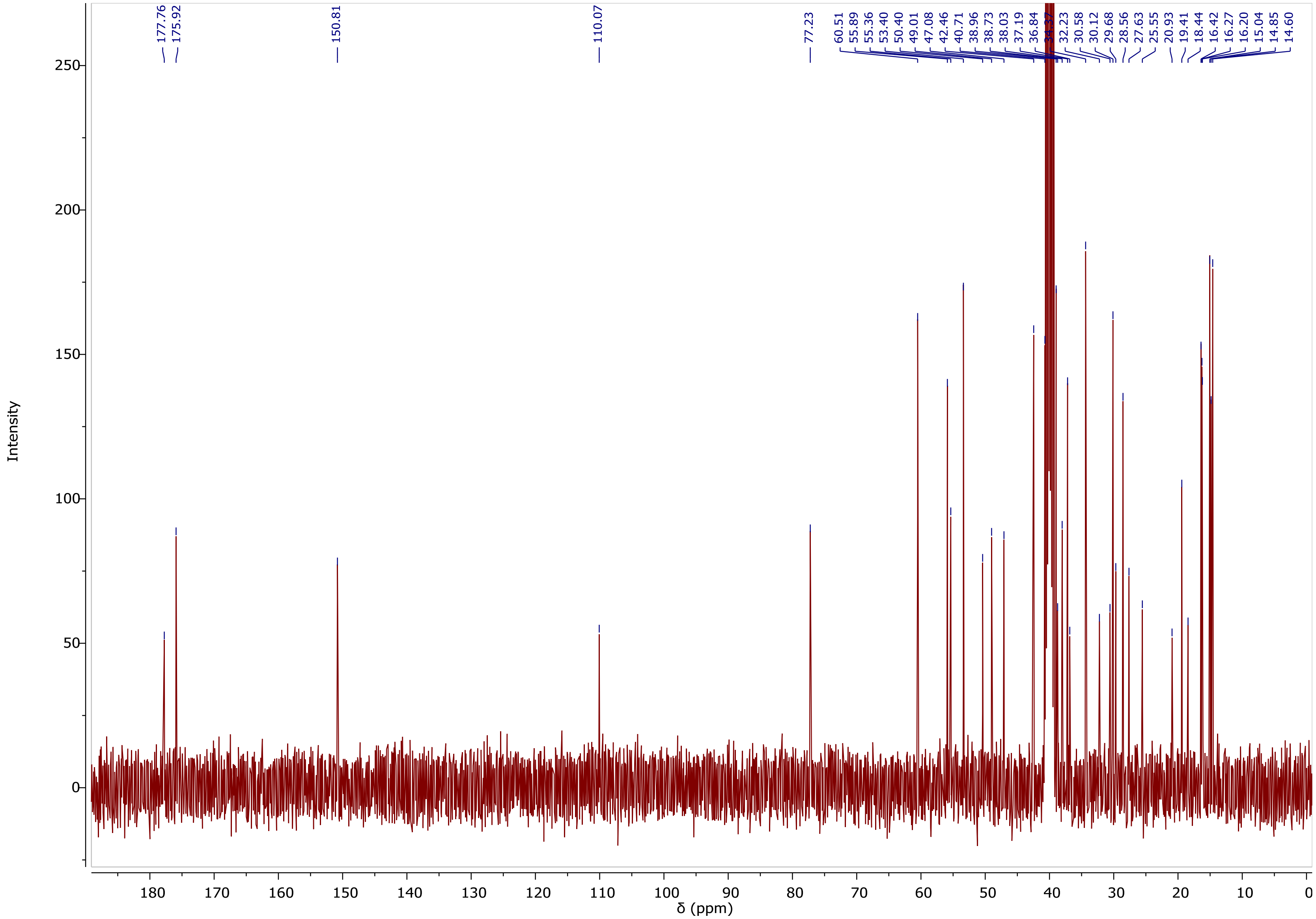

Figure S50. ^{13}C NMR spectrum of [MetOEt][BA].

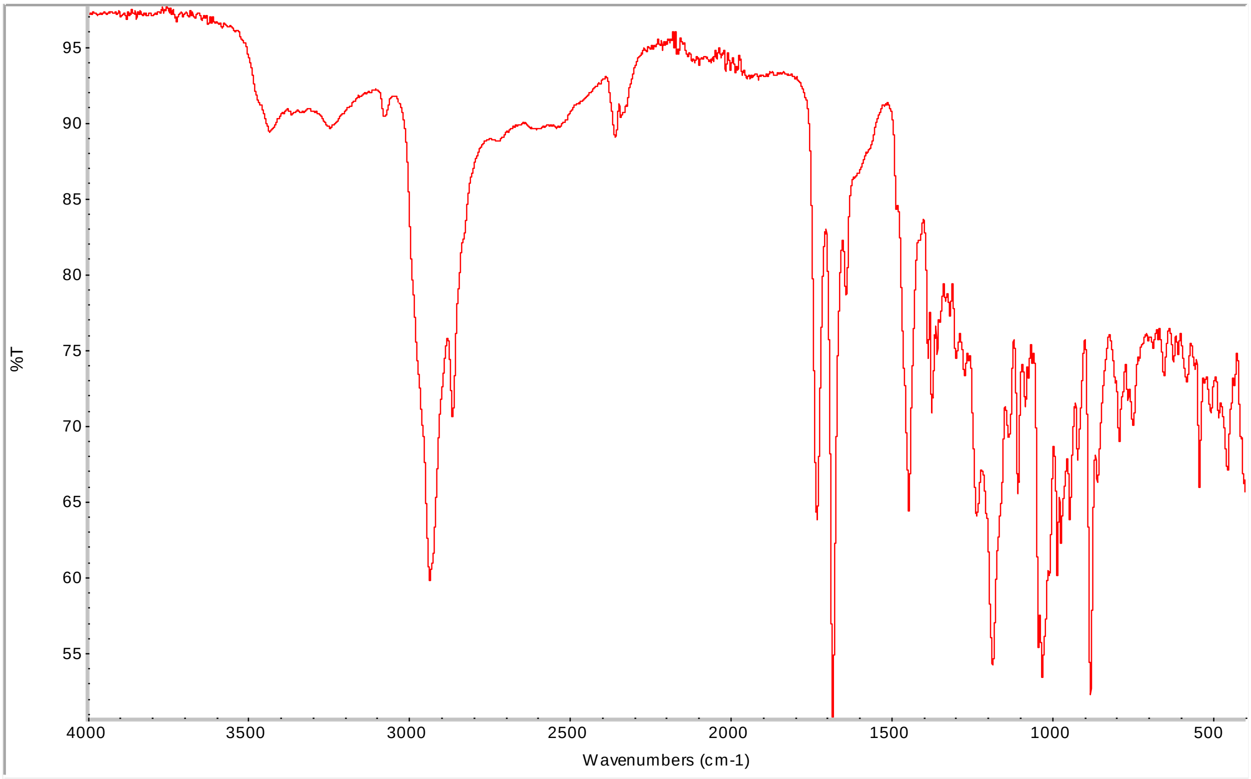

Figure S51. ATR-FTIR spectrum of [MetOEt][BA].

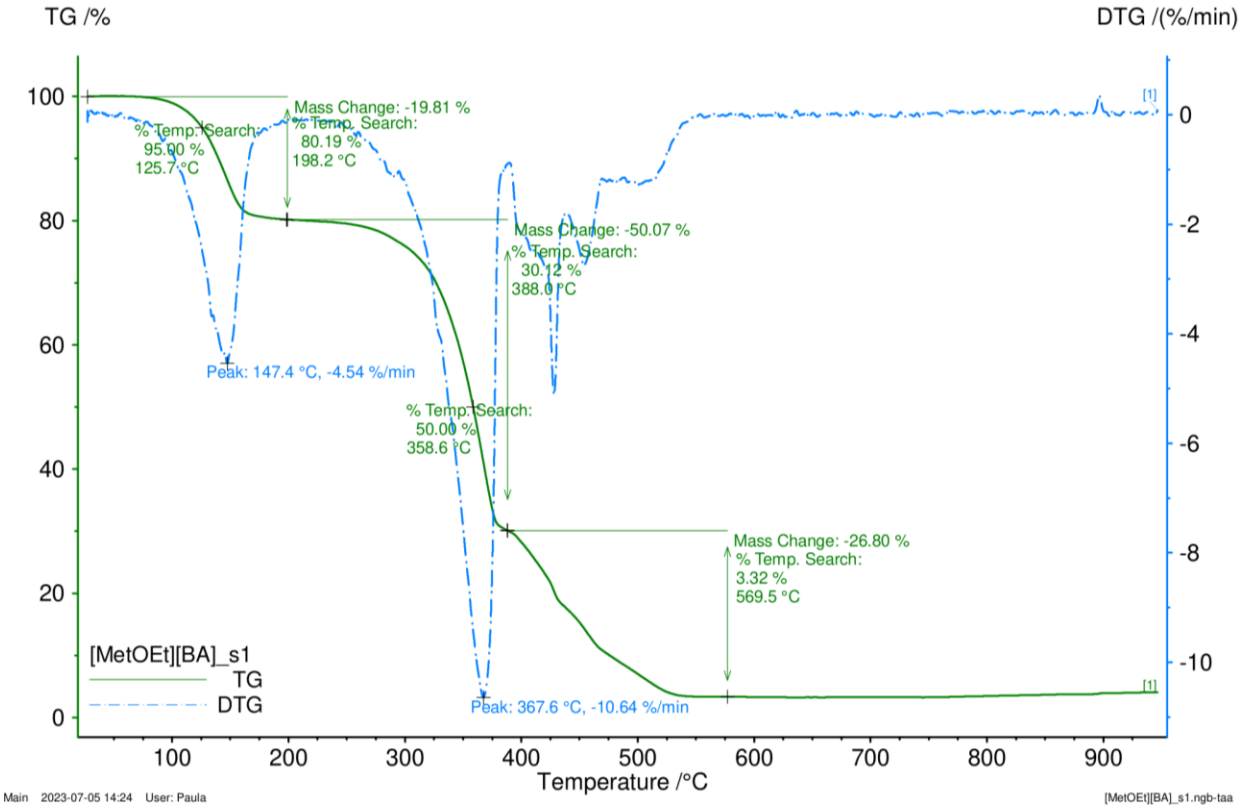

Figure S52. TG, DTG and c-DTA curves of [MetOEt][BA].

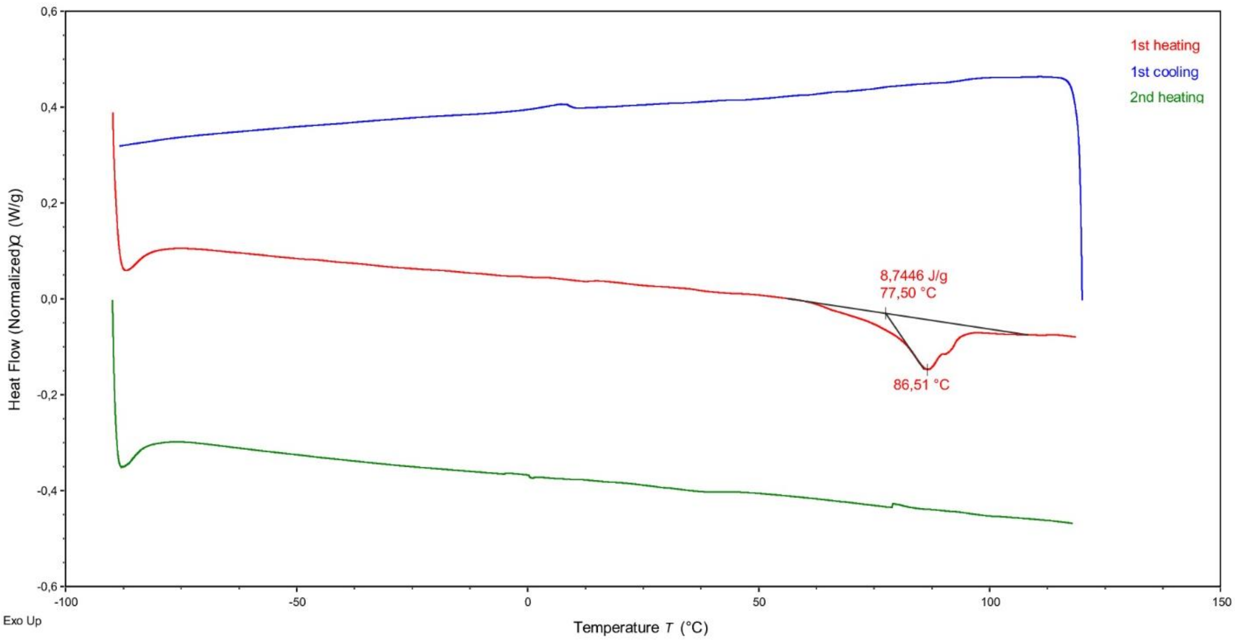

Figure S53. DSC curves of [MetOEt][BA].

L-lysine ethyl ester betulinate - [LysOEt][BA]

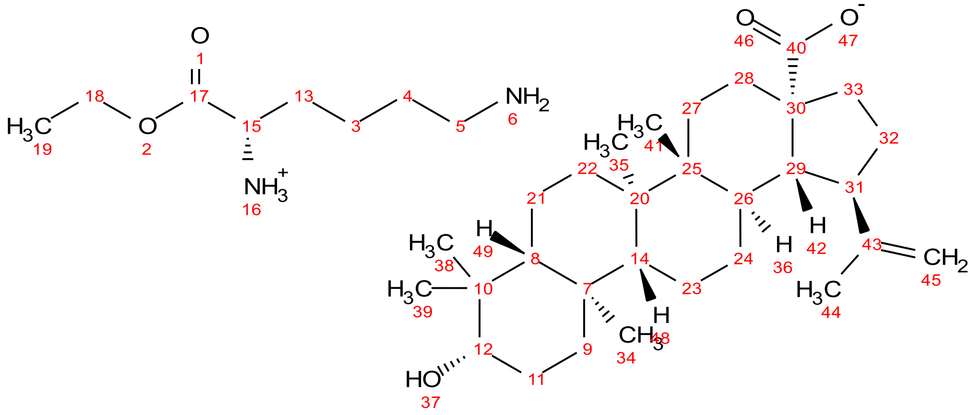

^{1}H NMR (400 MHz, DMSO-d_6) δ 4.65 (d, J = 2.7 Hz, 1H), 4.55 – 4.49 (m, 1H), 4.07 (qd, J = 7.1, 2.8 Hz, 5H), 3.28 – 3.20 (m, 1H), 3.04 (q, J = 6.8 Hz, 2H), 2.99 – 2.92 (m, 1H), 2.59 (t, J = 7.1 Hz, 2H), 2.43 – 2.32 (m, 1H), 2.12 (d, J = 12.2 Hz, 1H), 1.80 (dd, J = 17.9, 9.3 Hz, 2H), 1.63 (s, 4H), 1.60 – 1.47 (m, 4H), 1.47 – 1.31 (m, 14H), 1.31 (s, 4H), 1.28 – 1.12 (m, 11H), 1.06 – 0.93 (m, 3H), 0.91 (s, 3H), 0.89 – 0.84 (m, 7H), 0.81 (s, 2H), 0.76 (s, 3H), 0.64 (s, 5H). ^{13}C NMR (100 MHz, DMSO-d_6) δ 178.89, 176.28, 151.52, 109.62, 77.24, 60.33, 56.24, 55.40, 54.37, 50.50, 49.26, 42.48, 38.96, 37.90, 37.20, 34.86, 30.97, 28.58, 22.98, 21.03, 19.51, 16.42, 16.39, 16.28, 14.85, 14.62. FT–IR: ν (ATR): 3374, 3079, 2925, 2863, 1726, 1640, 1553, 1447, 1384, 1372, 1331, 1285, 1258, 1181, 1105, 1084, 1042, 1029, 1010, 984, 972, 943, 919, 879, 860, 830, 797, 765, 737, 702, 686, 624, 600, 571, 559, 545, 510, 484, 464, 444, 418. Elemental analysis: calc. (%) for $C_{38}H_{66}N_2O_5$ (630.955 g/mol): C (72.34%), H (10.54%), N (4.44%), O (12.68%); found: C (72.35%), H (10.53%), N (4.45%), O (12.68%).

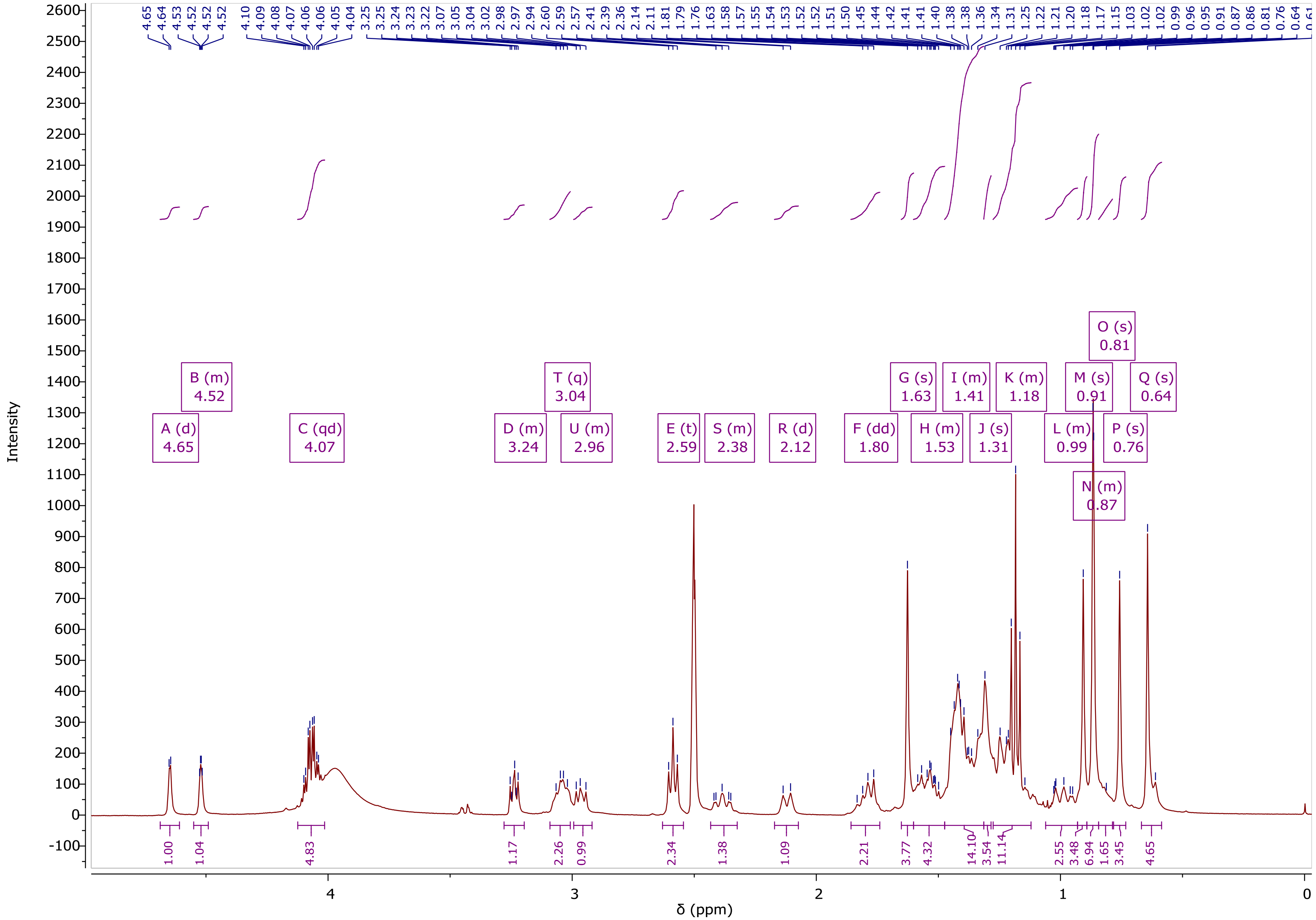

Figure S54. ^{1}H NMR spectrum of [LysOEt][BA].

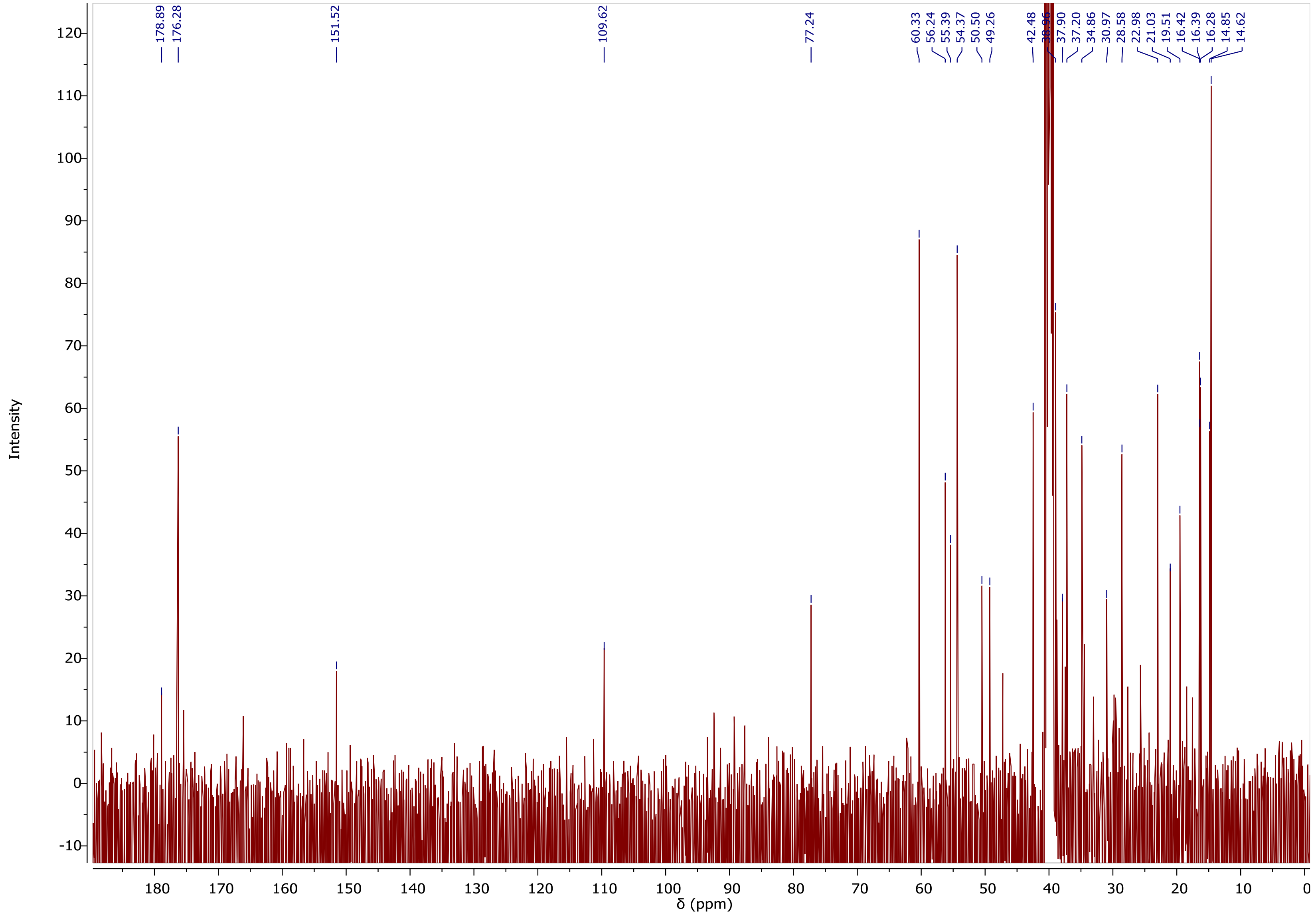

Figure S55. ^{13}C NMR spectrum of [LysOEt][BA].

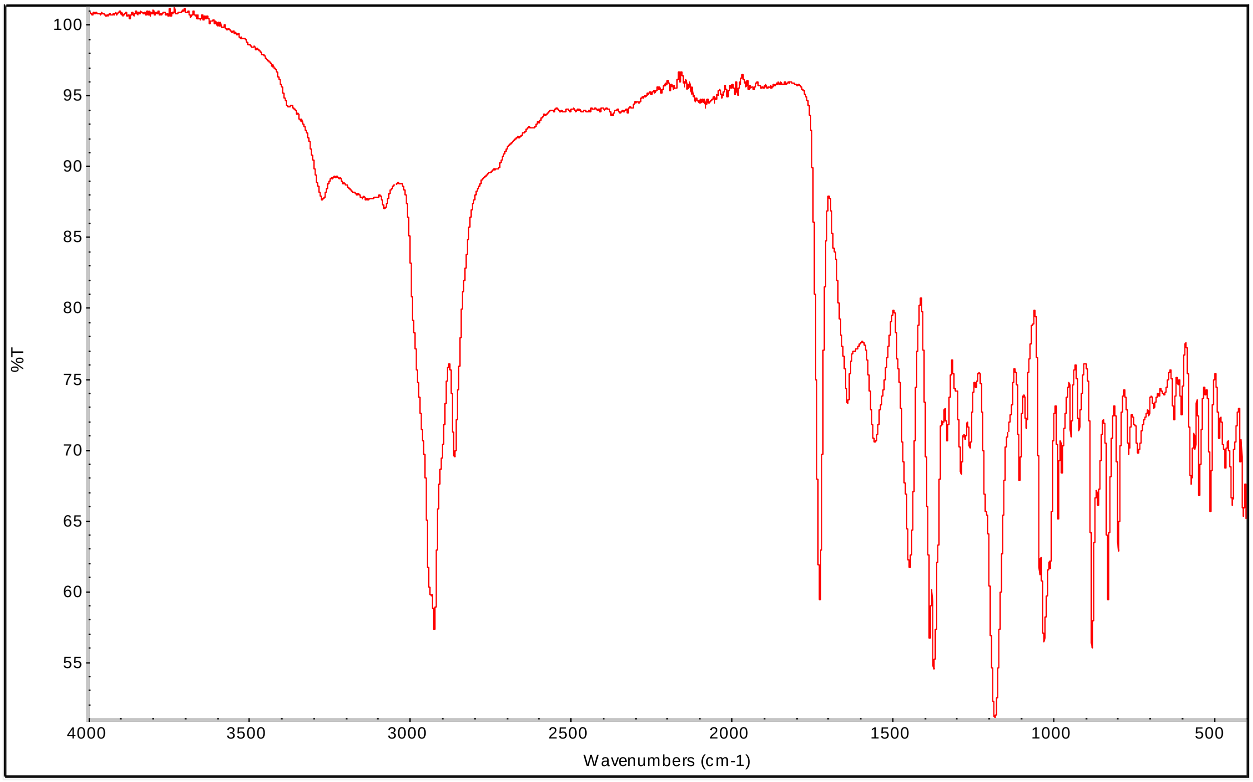

Figure S56. ATR-FTIR spectrum of [LysOEt][BA].

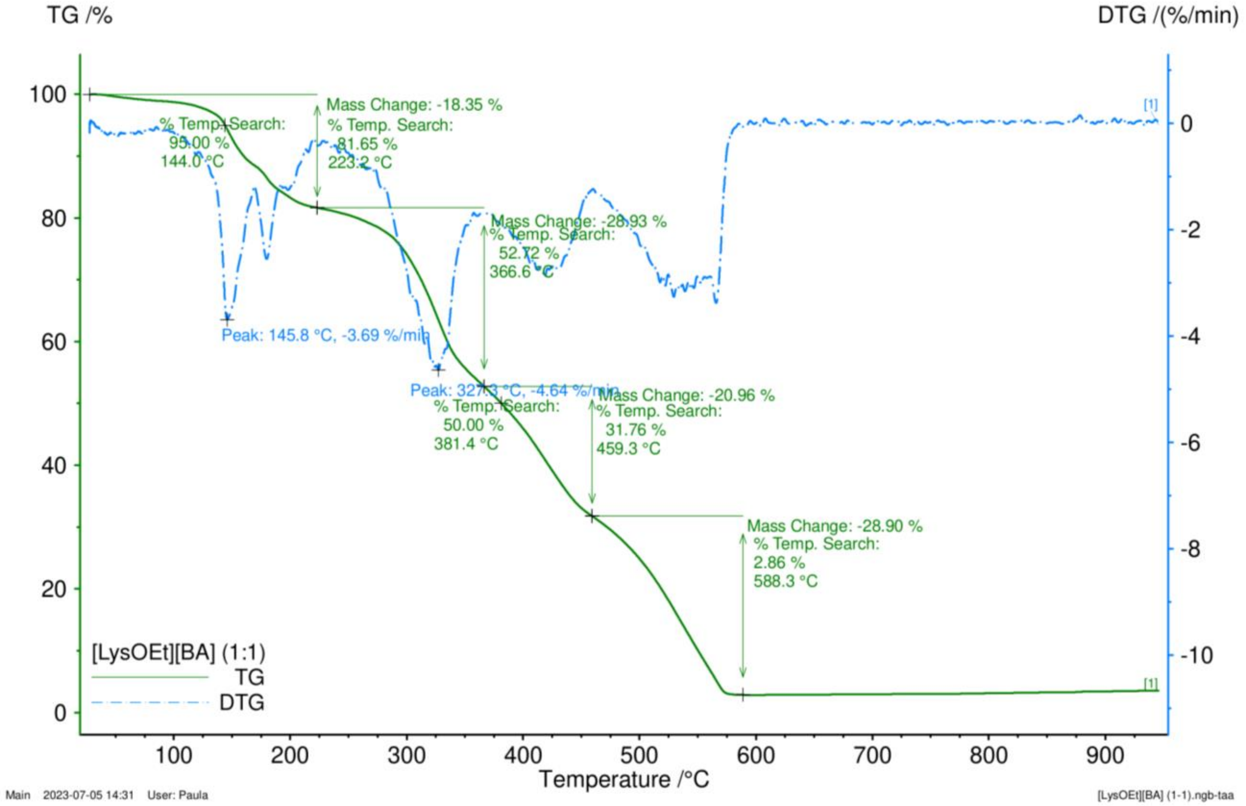

Figure S57. TG, DTG and c-DTA curves of [LysOEt][BA].

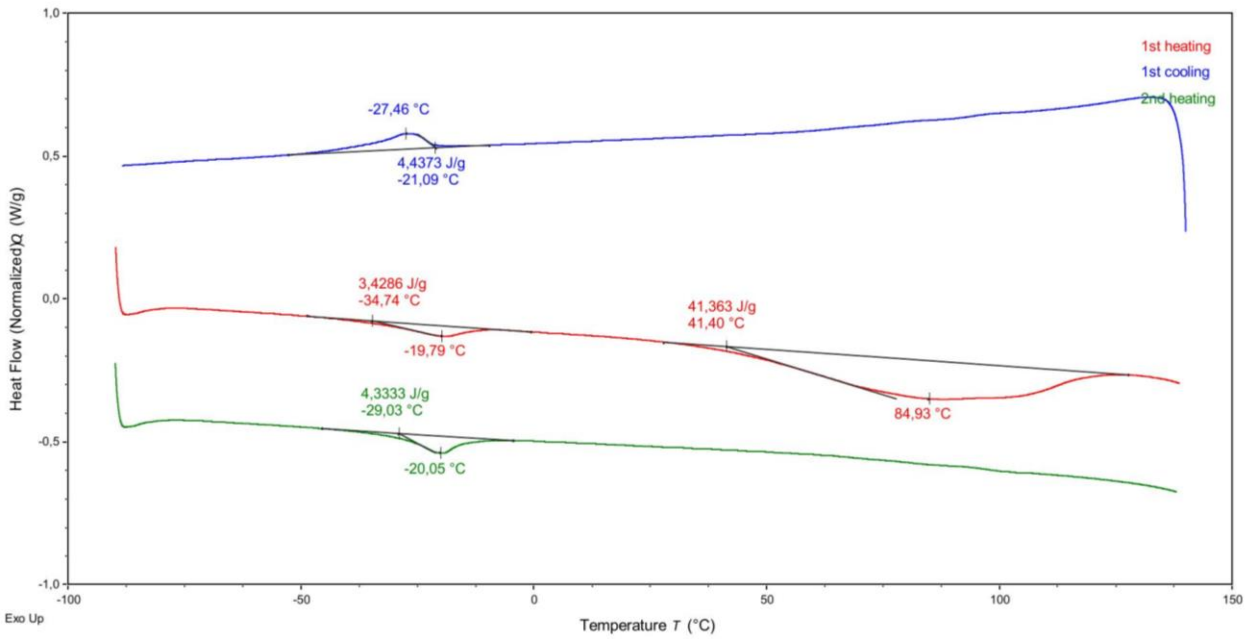

Figure S58. DSC curves of [LysOEt][BA].

L-lysine ethyl ester bis(betulinate) - [LysOEt][BA]$_2$

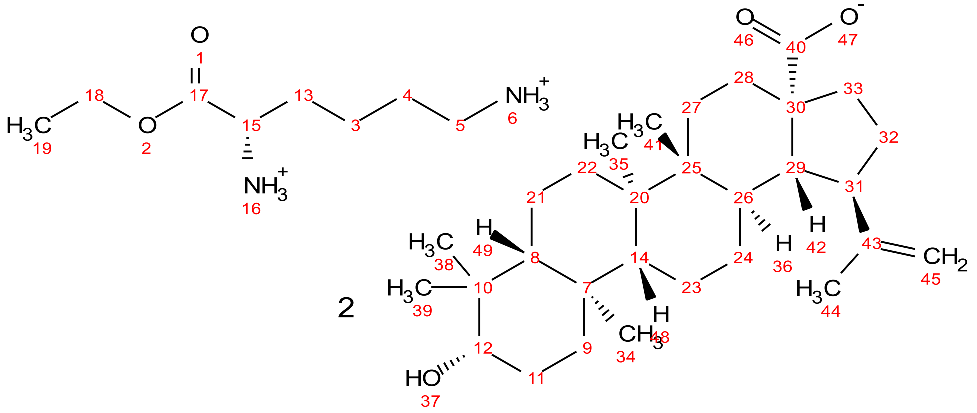

^{1}H NMR (400 MHz, DMSO-d_6) δ 5.76 (s, 1H), 4.66 (d, J = 2.6 Hz, 2H), 4.56 – 4.48 (m, 2H), 4.07 (qd, J = 7.1, 2.4 Hz, 2H), 3.24 (dd, J = 7.5, 5.5 Hz, 1H), 3.06 – 2.89 (m, 4H), 2.63 (t, J = 7.3 Hz, 2H), 2.34 (s, 1H), 2.12 (d, J = 12.4 Hz, 2H), 1.82 – 1.73 (m, 3H), 1.63 (s, 6H), 1.61 – 1.50 (m, 5H), 1.50 – 1.37 (m, 13H), 1.31 (s, 6H), 1.30 – 1.07 (m, 16H), 1.07 – 0.99 (m, 2H), 0.89 (d, J = 18.4 Hz, 22H), 0.76 (s, 6H), 0.64 (s, 8H). ^{13}C NMR (100 MHz, DMSO-d_6) δ 178.57, 176.23, 151.32, 109.73, 77.23, 60.34, 56.14, 55.39, 54.33, 50.47, 49.19, 47.19, 42.47, 40.72, 38.96, 38.75, 37.93, 37.20, 34.76, 34.46, 32.80, 30.82, 29.78, 28.57, 27.64, 25.65, 21.00, 19.48, 18.46, 16.42, 16.34, 16.27, 14.85, 14.62. FT–IR: ν (ATR): 3361, 3072, 2937, 2864, 1732, 1684, 1640, 1529, 1447, 1385, 1372, 1328, 1297, 1275, 1255, 1234, 1185, 1140, 1107, 1083, 1064, 1043, 1032, 1010, 983, 972, 945, 920, 880, 861, 831, 794, 765, 747, 703, 686, 653, 624, 577, 543, 510, 472, 464, 457, 445. Elemental analysis: calc. (%) for $C_{68}H_{114}N_2O_8$ (1087.666 g/mol): C (75.09%), H (10.57%), N (2.58%), O (11.77%); found: C (75.10%), H (10.56%), N (2.58%), O (11.77%).

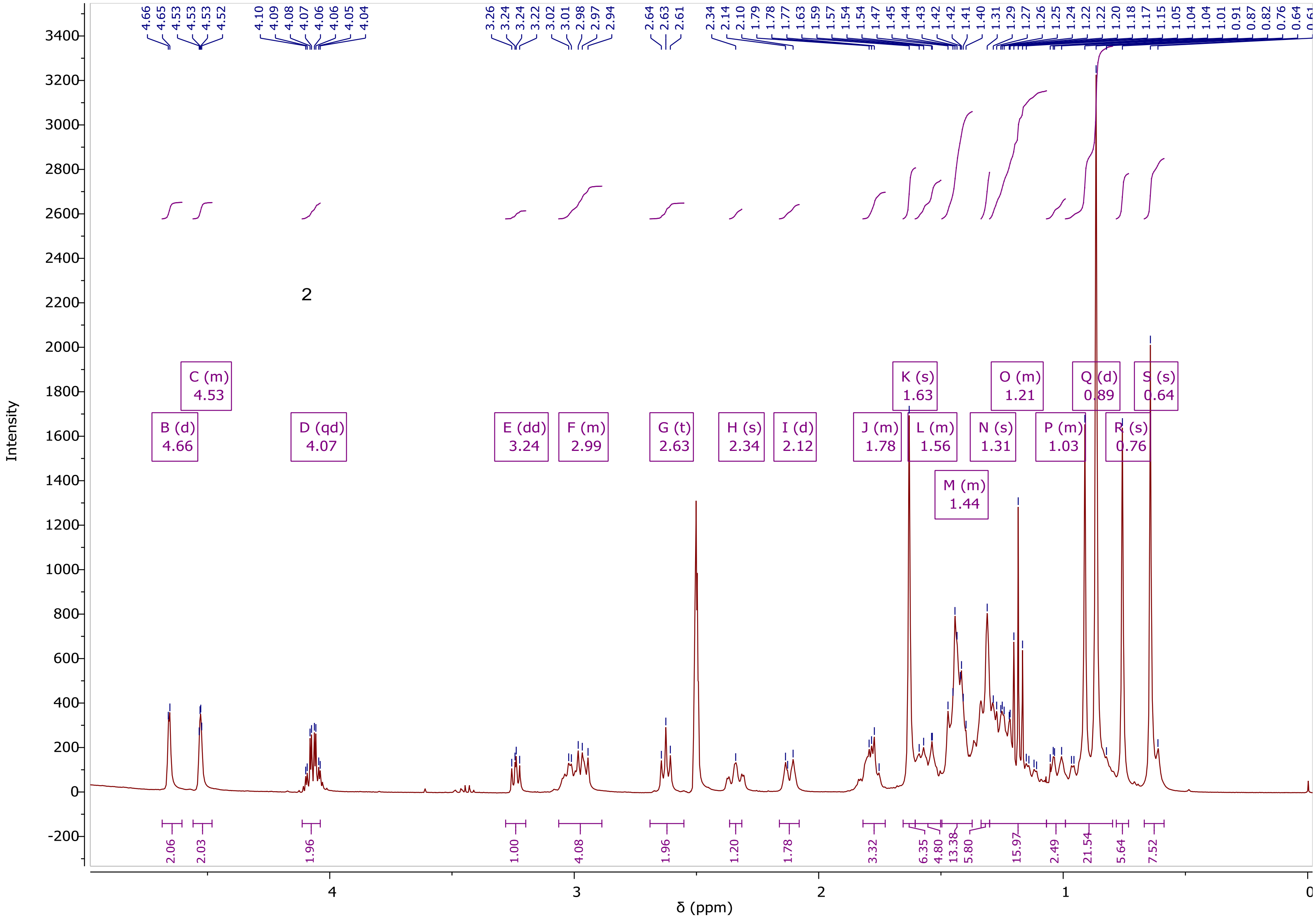

Figure S59. ^{1}H NMR spectrum of [LysOEt][BA]$_2$.

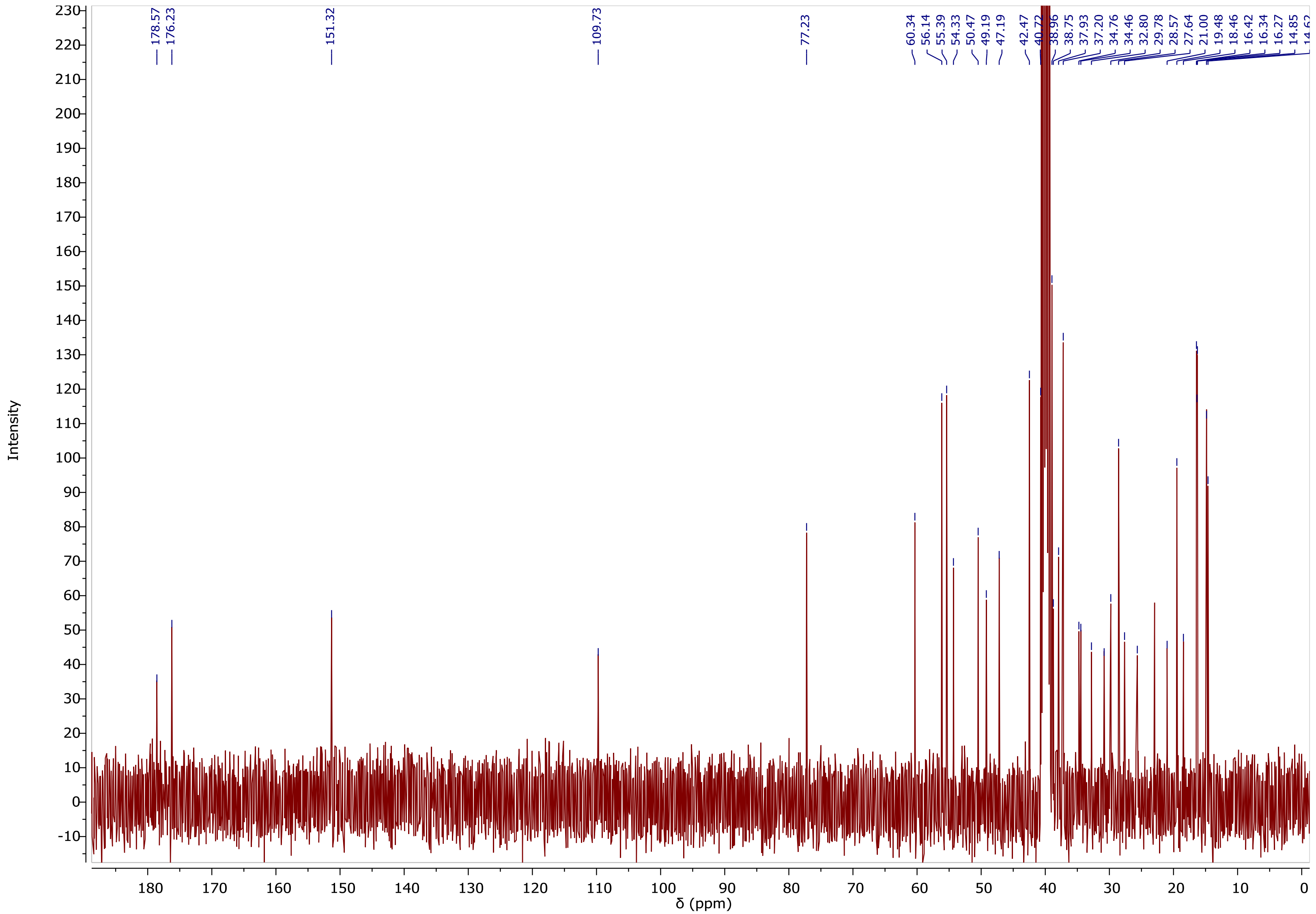

Figure S60. ^{13}C NMR spectrum of [LysOEt][BA]$_2$.

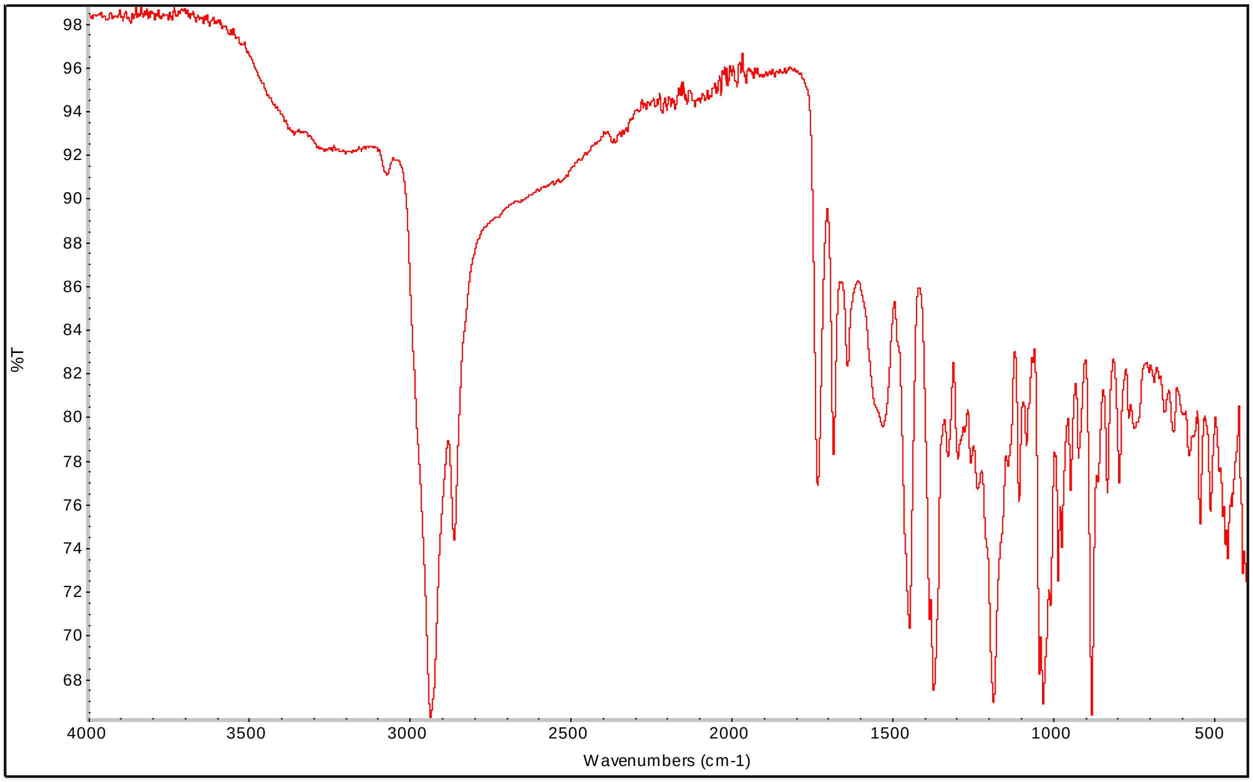

Figure S61. ATR-FTIR spectrum of [LysOEt][BA]$_2$.

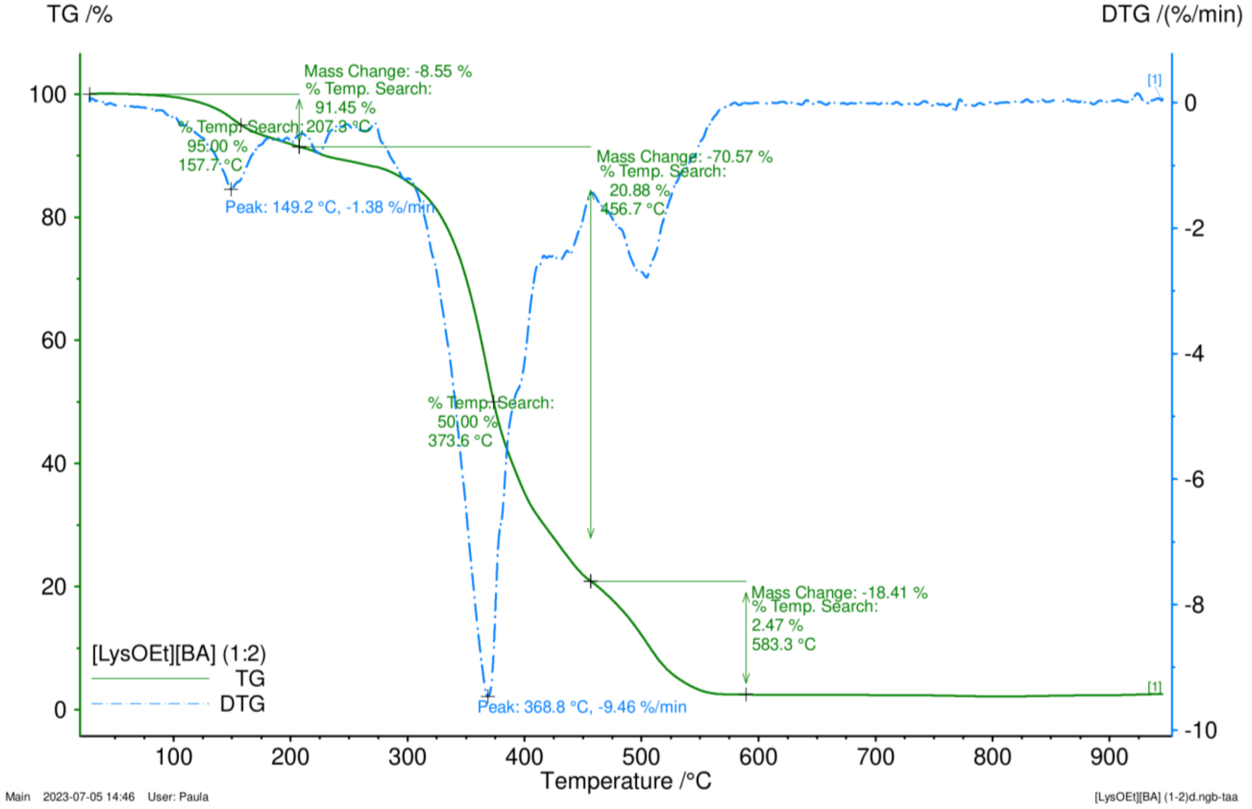

Figure S62. TG, DTG and c-DTA curves of [LysOEt][BA]$_2$.

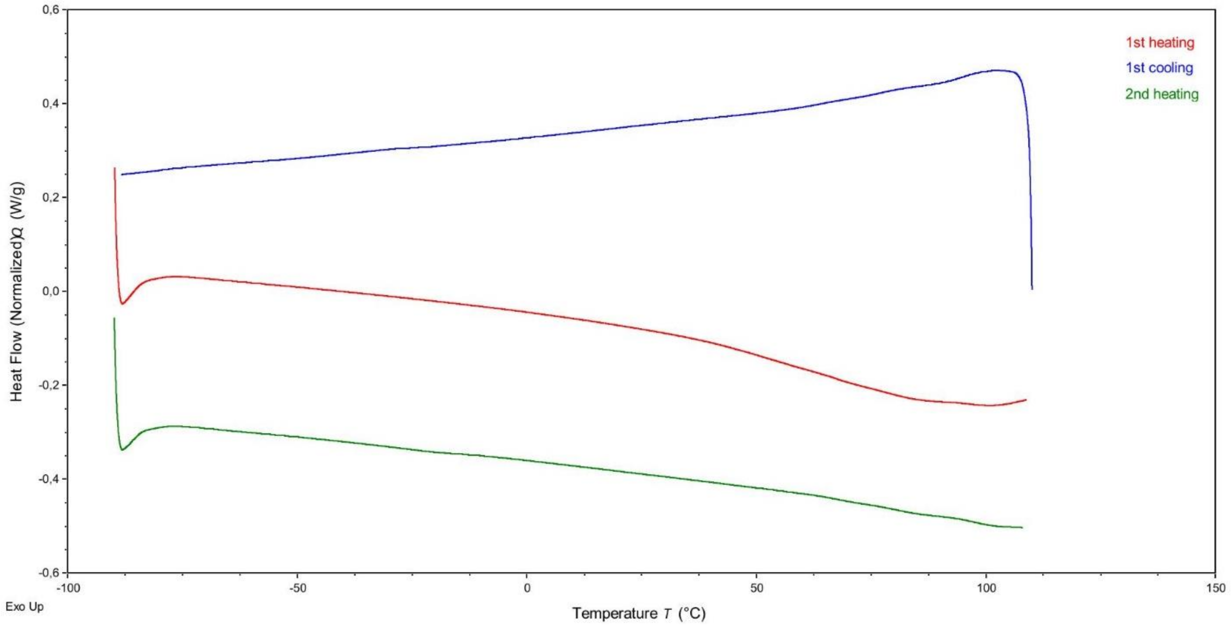

Figure S63. DSC curves of [LysOEt][BA]$_2$.

L-phenylalanine ethyl ester betulinate - [PheOEt][BA]

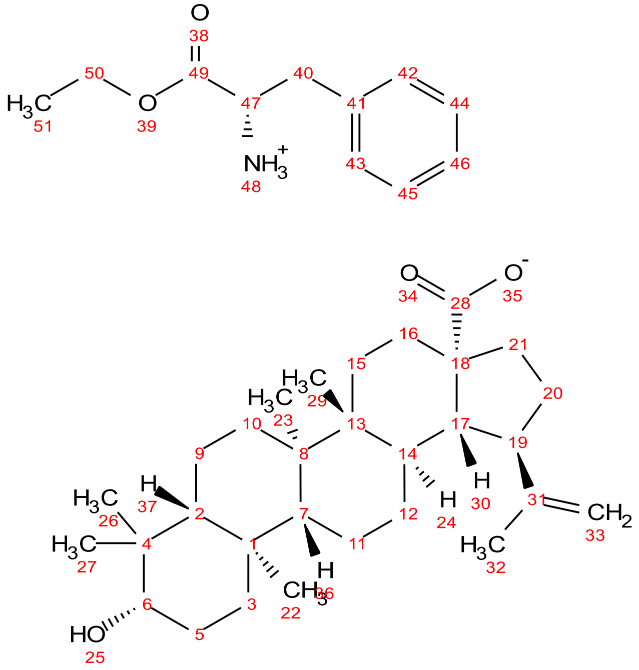

^{1}H NMR (400 MHz, DMSO-d_6) δ 7.32 – 7.14 (m, 5H), 4.69 (d, J = 2.5 Hz, 1H), 4.56 (t, J = 2.0 Hz, 1H), 4.06 – 3.98 (m, 2H), 3.54 (t, J = 6.8 Hz, 1H), 2.95 (td, J = 10.6, 5.8 Hz, 2H), 2.81 (qd, J = 13.3, 6.8 Hz, 2H), 2.12 (d, J = 10.1 Hz, 1H), 1.79 (q, J = 5.6, 4.8 Hz, 2H), 1.64 (s, 3H), 1.59 – 1.42 (m, 5H), 1.40 – 1.29 (m, 5H), 1.29 – 1.21 (m, 2H), 1.10 (t, J = 7.1 Hz, 5H), 0.93 (s, 3H), 0.87 (d, J = 1.7 Hz, 6H), 0.76 (s, 2H), 0.65 (s, 3H). ^{13}C NMR (100 MHz, DMSO-d_6) δ 177.75, 175.38, 150.80, 138.37, 129.70, 128.54, 126.71, 110.09, 77.23, 60.34, 56.21, 55.89, 55.35, 50.39, 49.00,

47.08, 42.46, 41.33, 40.71, 38.96, 38.72, 38.03, 37.19, 36.83, 34.38, 32.21, 30.57, 29.68, 28.56, 27.63, 25.54, 20.92, 19.41, 18.43, 16.42, 16.28, 16.20, 14.85, 14.49. FT–IR: ν (ATR): 3336, 3068, 3027, 2980, 2971, 2939, 2928, 2866, 1736, 1682, 1639, 1619, 1606, 1576, 1496, 1484, 1447, 1386, 1375, 1360, 1332, 1300, 1231, 1182, 1105, 1078, 1043, 1032, 1009, 983, 972, 955, 944, 915, 891, 876, 857, 792, 743, 698, 651, 623, 596, 541, 511, 482, 454, 437. Elemental analysis: calc. (%) for $C_{41}H_{63}NO_5$ (649.957 g/mol): C (75.77%), H (9.77%), N (2.16%), O (12.31%); found: C (75.11%), H (10.55%), N (2.59%), O (11.78%).

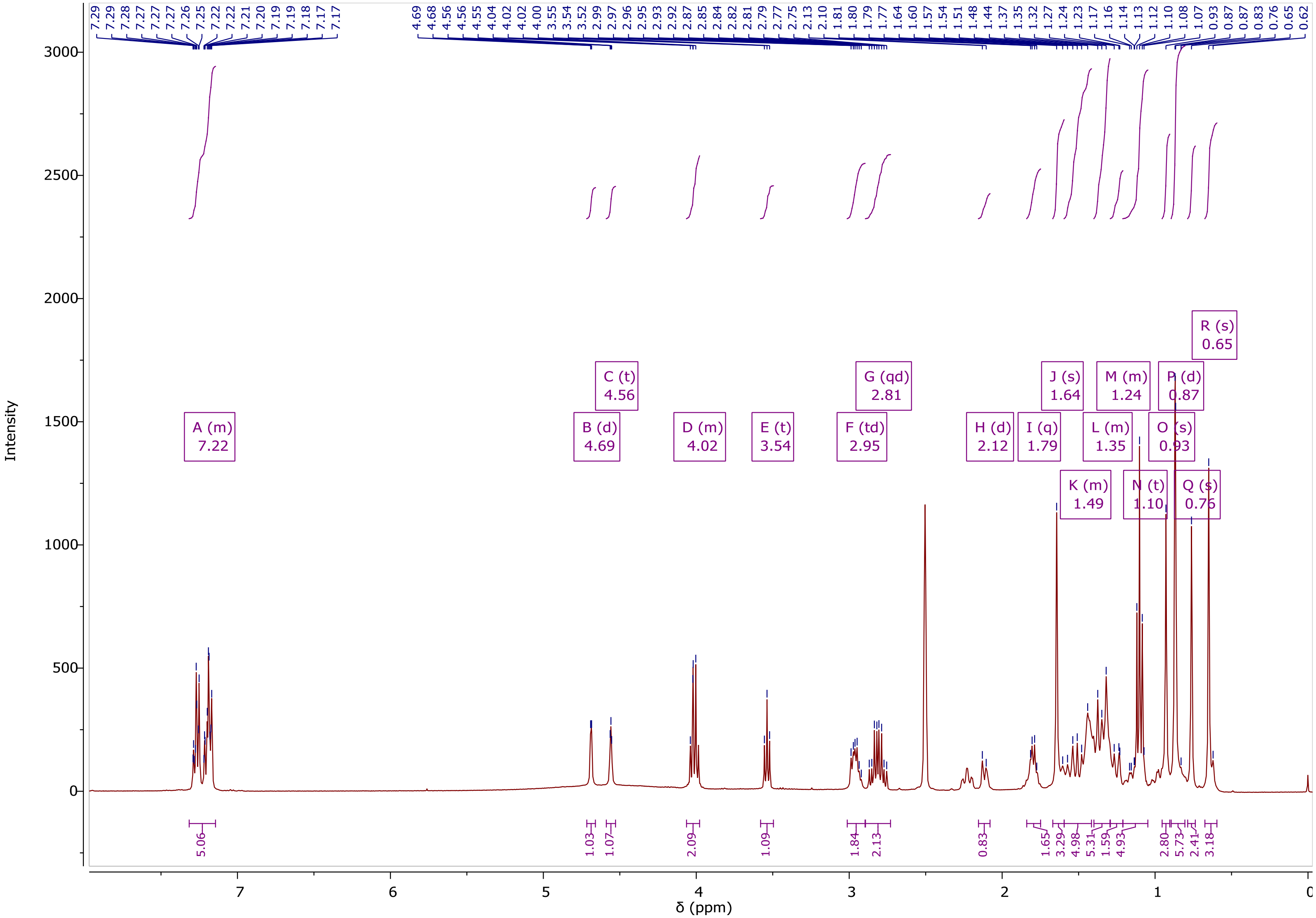

Figure S64. ^{1}H NMR spectrum of [PheOEt][BA].

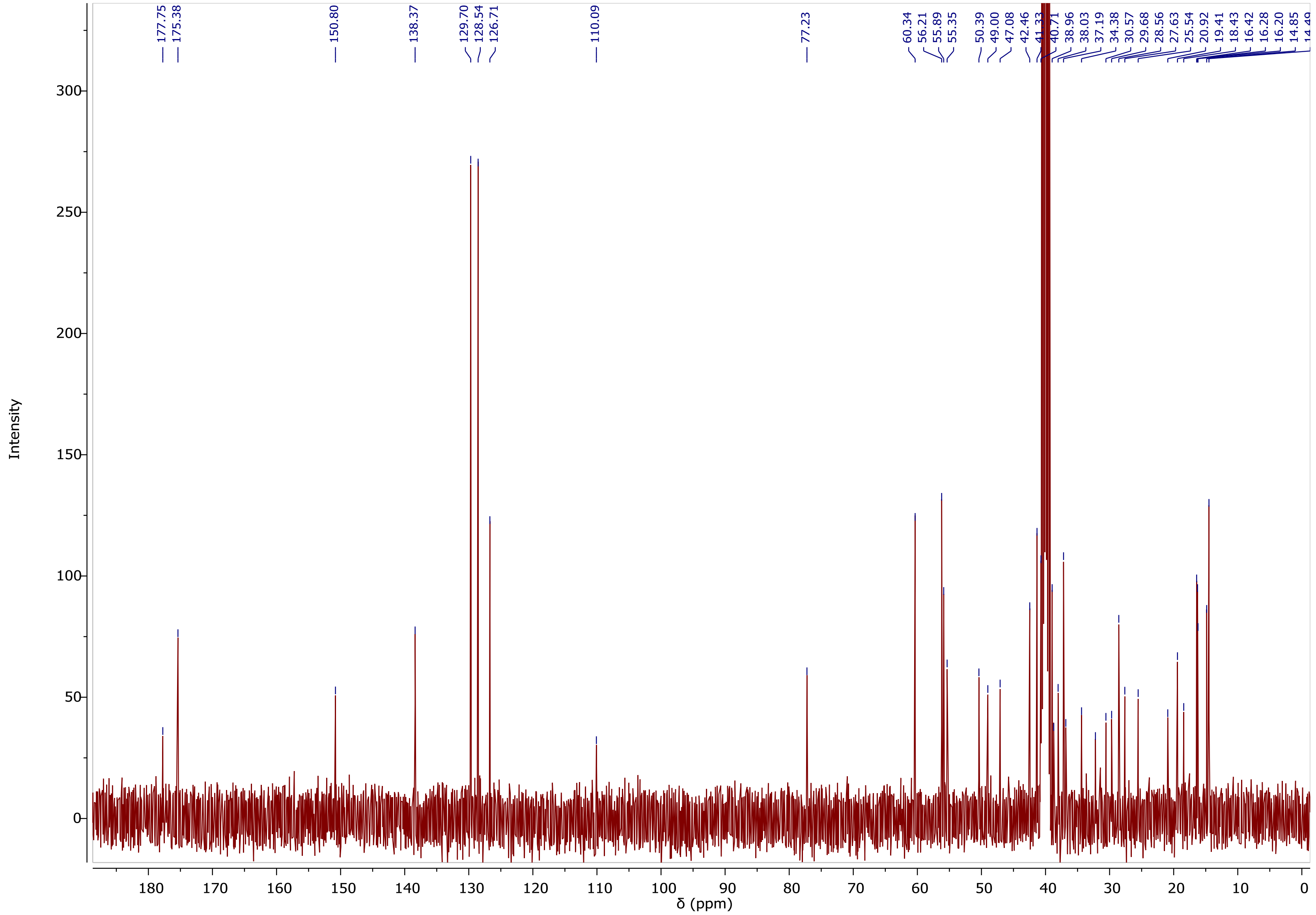

Figure S65. ^{13}C NMR spectrum of [PheOEt][BA].

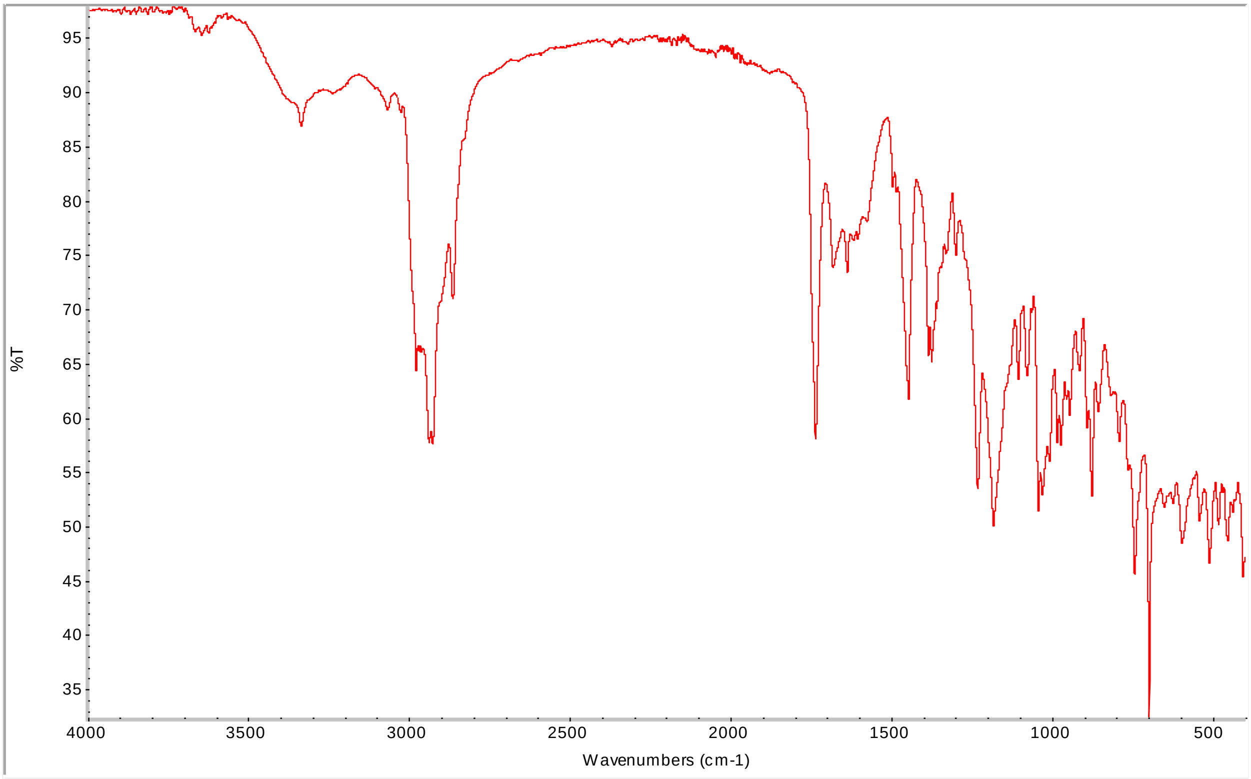

Figure S66. ATR-FTIR spectrum of [PheOEt][BA].

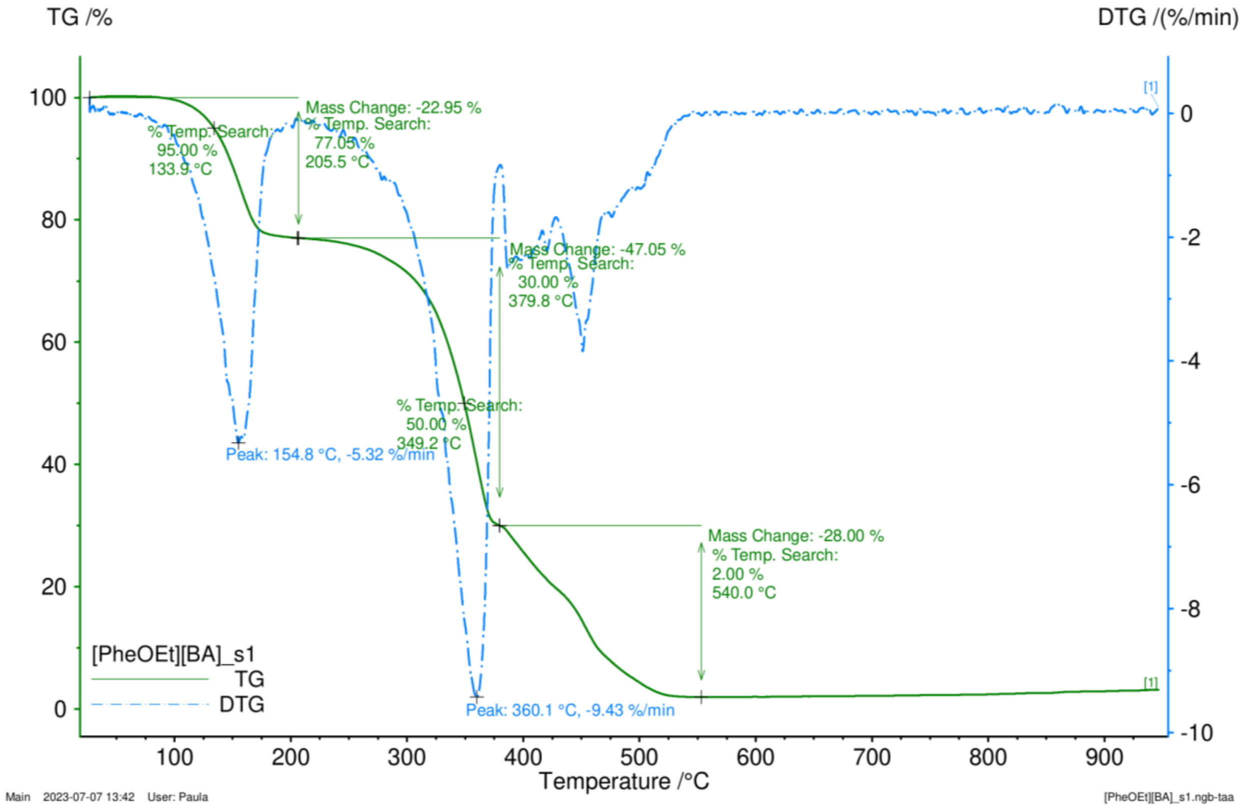

Figure S67. TG, DTG and c-DTA curves of [PheOEt][BA].

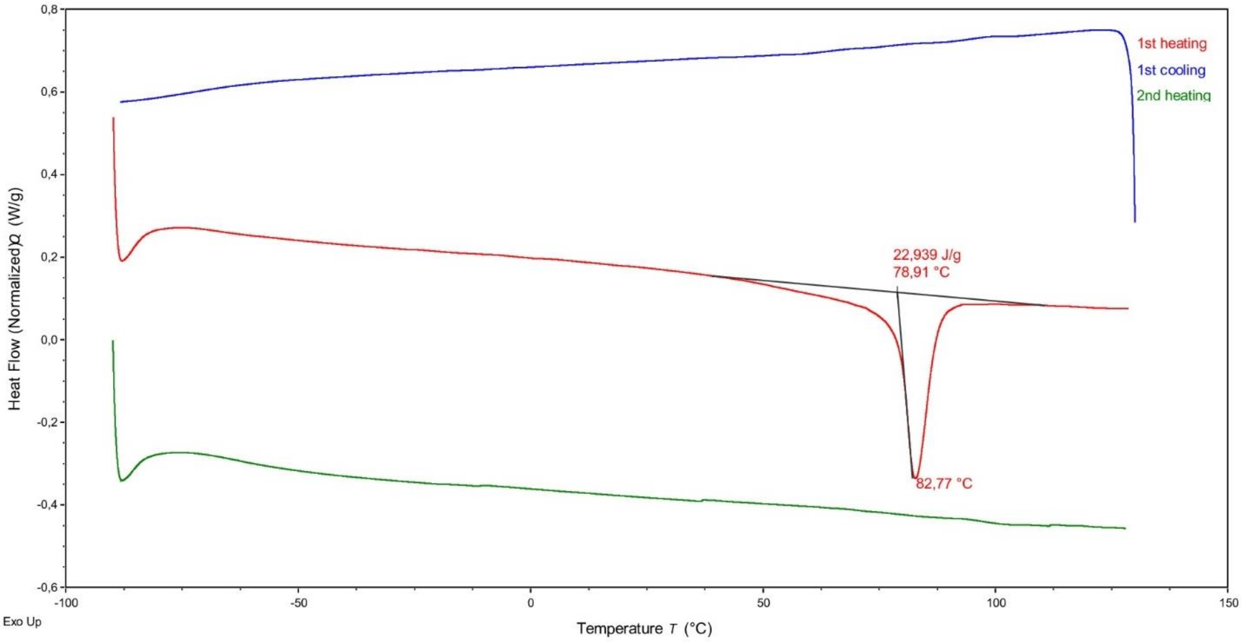

Figure S68. DSC curves of [PheOEt][BA].

L-proline ethyl ester betulinate - [ProOEt][BA]

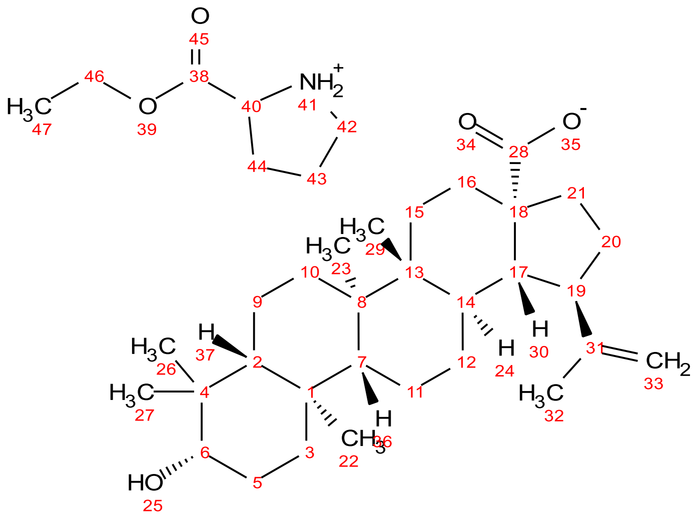

^{1}H NMR (400 MHz, DMSO-d_6) δ 4.69 (d, J = 2.7 Hz, 1H), 4.59 – 4.53 (m, 1H), 4.07 (q, J = 7.1 Hz, 2H), 3.63 (dd, J = 8.6, 5.3 Hz, 1H), 3.01 – 2.83 (m, 3H), 2.75 (ddd, J = 10.0, 7.0, 6.1 Hz, 1H), 2.15 – 2.08 (m, 1H), 2.02 – 1.90 (m, 1H), 1.84 – 1.79 (m, 1H), 1.79 – 1.70 (m, 2H), 1.70 – 1.38 (m, 14H), 1.38 – 1.30 (m, 5H), 1.30 – 1.23 (m, 2H), 1.19 (t, J = 7.1 Hz, 4H), 1.10 – 0.93 (m, 3H), 0.93 (s, 3H), 0.89 – 0.80 (m, 7H), 0.76 (s, 3H), 0.65 (s, 4H).^{13}C NMR (100 MHz, DMSO-d_6) δ 177.78, 175.22, 150.80, 110.07, 77.23, 60.52, 59.52, 55.89, 55.36, 50.40, 49.01, 47.08, 46.87, 42.46, 40.71, 38.96, 38.73, 38.03, 37.18, 36.84, 34.38, 32.22, 30.58, 30.09, 29.68, 28.56, 27.63, 25.67, 25.55, 20.92, 19.41, 18.44, 16.42, 16.27, 16.19, 14.85, 14.57. FT–IR: ν (ATR): 3190, 3079, 2985, 2940, 2925, 2863, 1742, 1639, 1590, 1481, 1451, 1386, 1371, 1327, 1306, 1258, 1210, 1184, 1130, 1104, 1083, 1059, 1043, 1032, 1010, 984, 971, 943, 918, 877, 859, 827, 796, 765, 748, 701, 653, 623, 610, 600, 569, 544, 511, 486, 469. Elemental analysis: calc. (%) for $C_{37}H_{61}NO_5$ (599.897 g/mol): C (74.08%), H (10.25%), N (2.33%), O (13.33%); found: C (74.10%), H (10.24%), N (2.33%), O (13.34%).

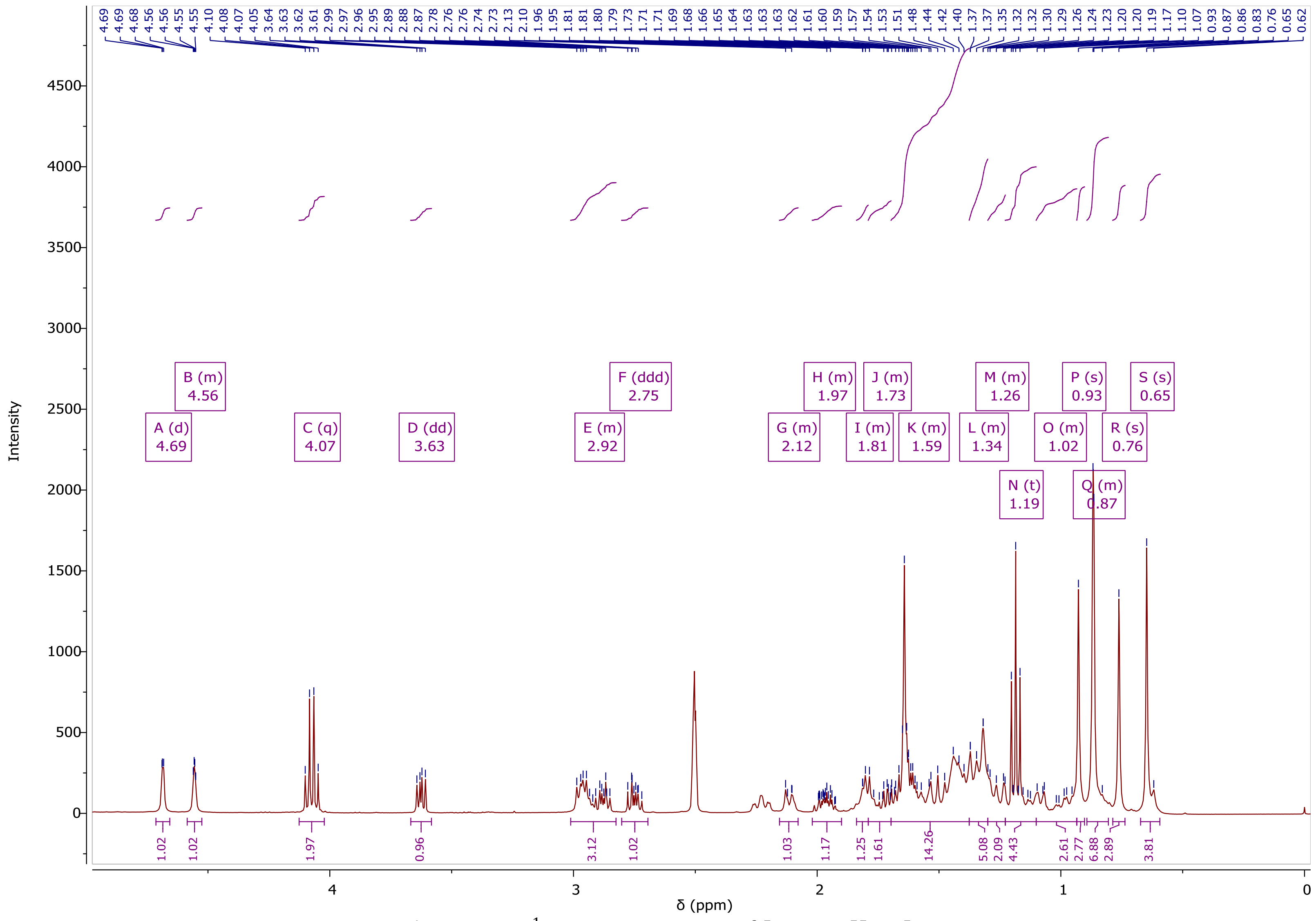

Figure S69. ^{1}H NMR spectrum of [ProOEt][BA].

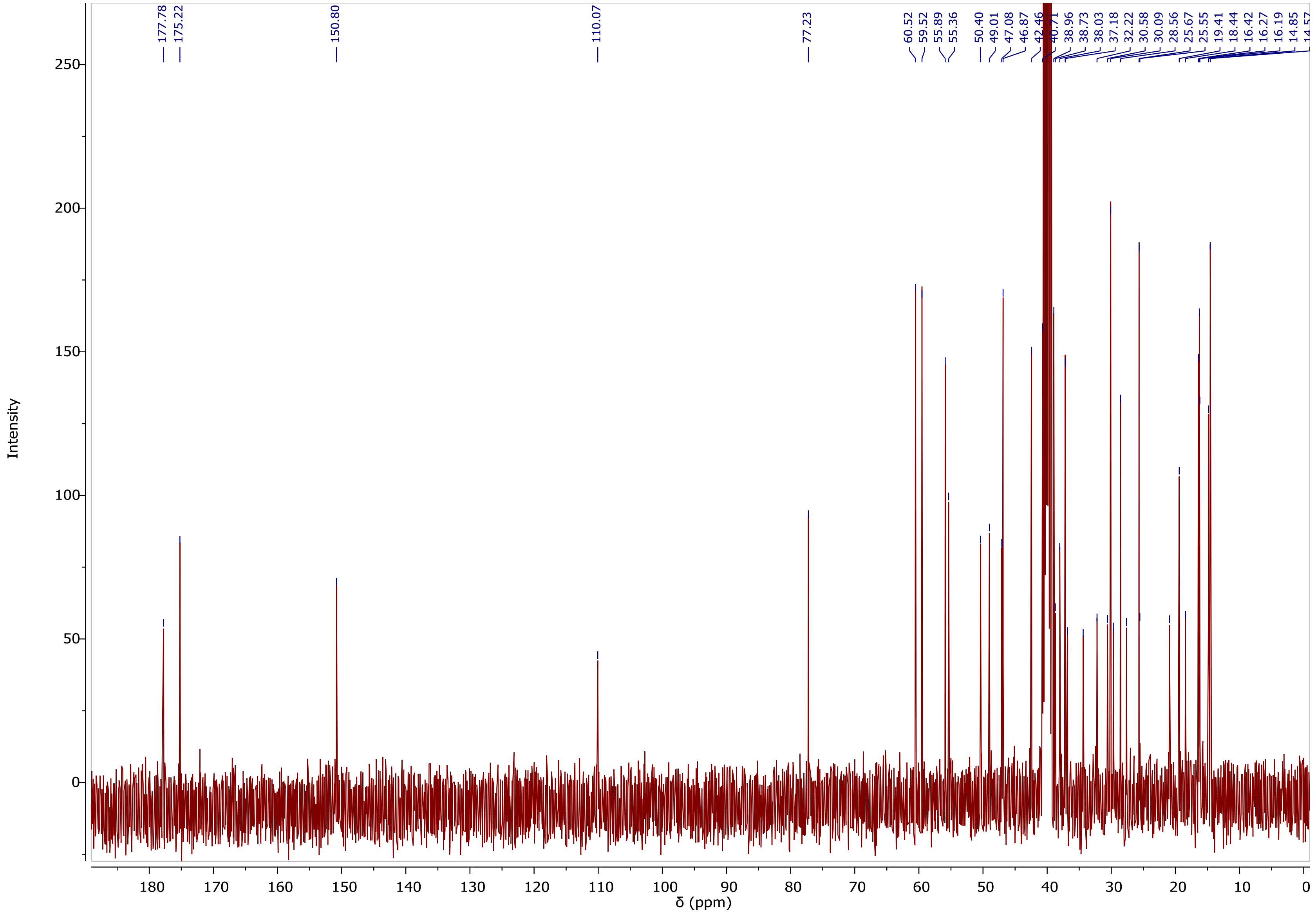

Figure S70. ^{13}C NMR spectrum of [ProOEt][BA].

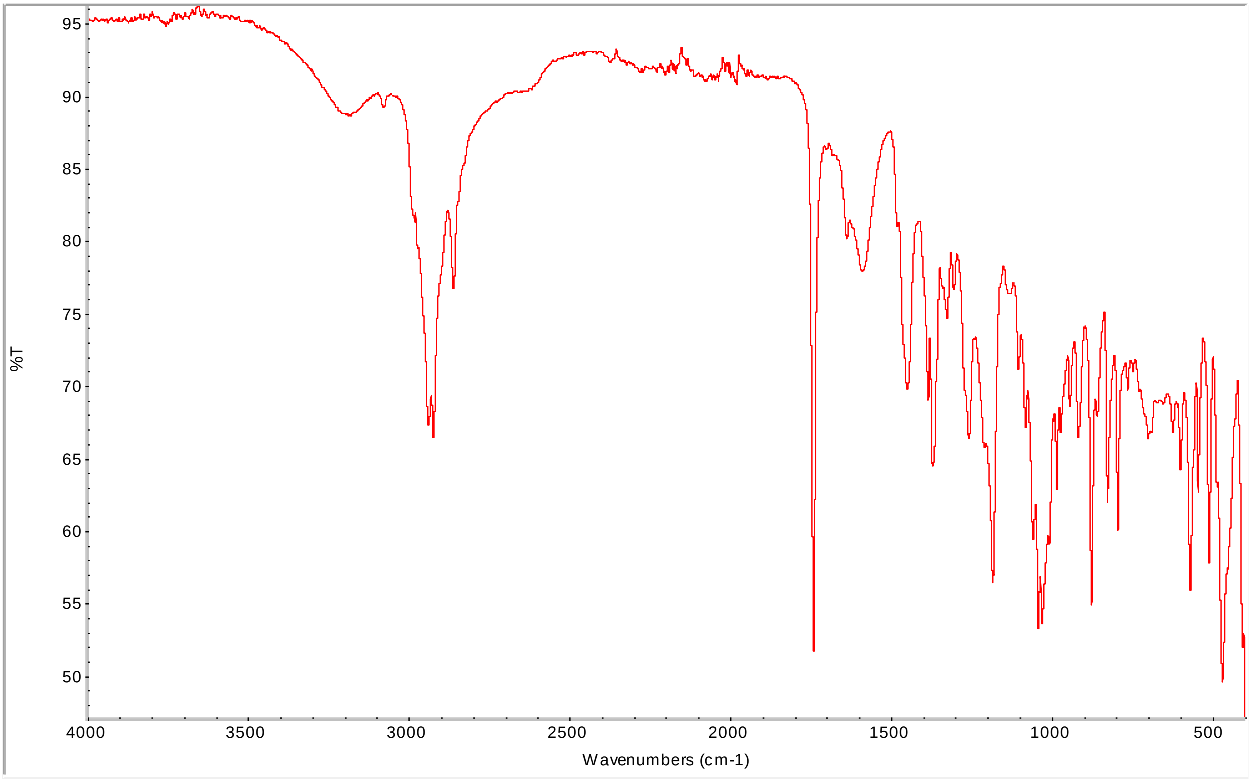

Figure S71. ATR-FTIR spectrum of [ProOEt][BA].

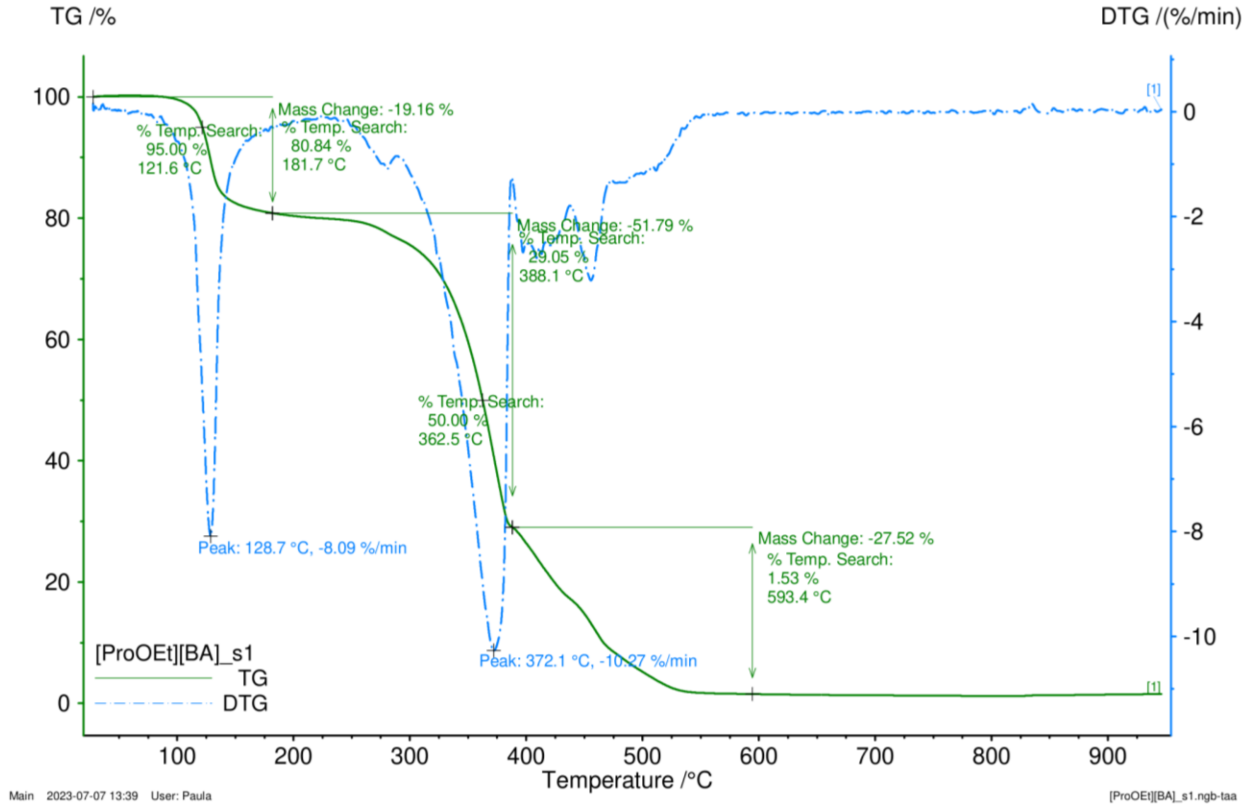

Figure S72. TG, DTG and c-DTA curves of [ProOEt][BA].

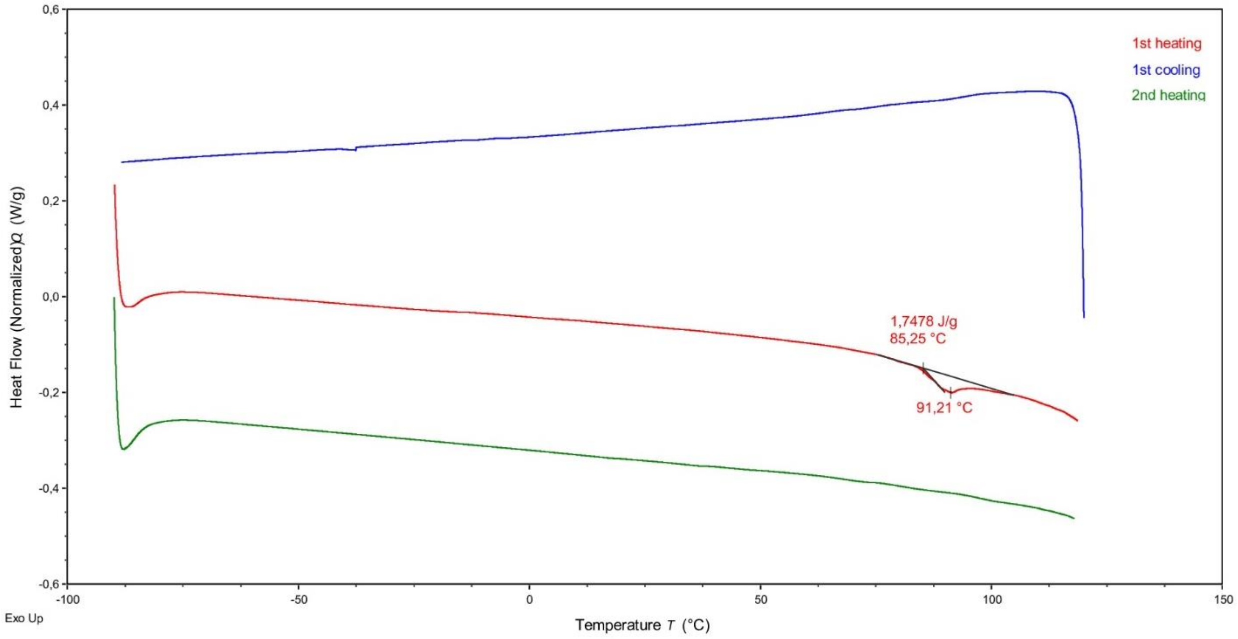

Figure S73. DSC curves of [ProOEt][BA].

L-tyrosine ethyl ester betulinate - [TyrOEt][BA]

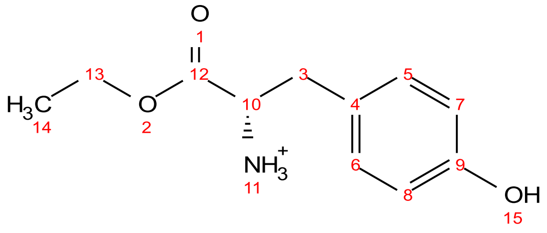

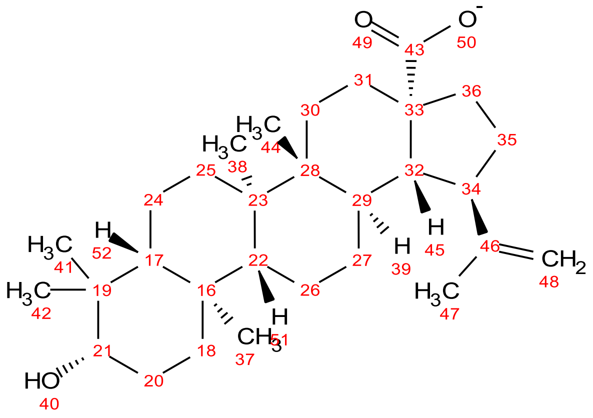

^{1}H NMR (400 MHz, DMSO-d_6) δ 6.98 – 6.91 (m, 2H), 6.68 – 6.57 (m, 2H), 4.71 – 4.65 (m, 1H), 4.58 – 4.52 (m, 1H), 4.04 – 3.96 (m, 2H), 3.46 (t, J = 6.7 Hz, 1H), 2.97 (dd, J = 9.8, 6.1 Hz, 2H), 2.77 – 2.62 (m, 2H), 2.32 – 2.17 (m, 1H), 2.12 (d, J = 10.4 Hz, 1H), 1.83 – 1.74 (m, 2H), 1.64 (s, 3H), 1.56 – 1.46 (m, 2H), 1.39 (dd, J = 28.8, 9.0 Hz, 7H), 1.32 – 1.21 (m, 4H), 1.11 (t, J = 7.1 Hz, 5H), 1.04 – 0.84 (m, 10H), 0.76 (s, 3H), 0.65 (s, 4H). ^{13}C NMR (100 MHz, DMSO-d_6) δ 177.76,

175.45, 156.29, 150.82, 130.57, 128.19, 115.36, 110.07, 77.23, 60.28, 56.37, 55.89, 55.36, 50.40, 49.01, 47.08, 42.46, 40.71, 40.55, 38.96, 38.72, 38.03, 37.19, 36.84, 34.38, 32.22, 30.58, 29.68, 28.56, 27.63, 25.55, 20.92, 19.41, 18.44, 16.42, 16.28, 16.20, 14.85, 14.53. FT–IR: ν (ATR): 3424, 3240, 3076, 2939, 2867, 2722, 1726, 1684, 1642, 1616, 1516, 1484, 1447, 1410, 1387, 1375, 1359, 1351, 1333, 1319, 1297, 1272, 1235, 1205, 1191, 1157, 1135, 1107, 1084, 1075, 1063, 1043, 1032, 1010, 983, 972, 945, 920, 881, 861, 828, 804, 793, 766, 747, 731, 703, 686, 651, 621, 608, 577, 556, 542, 523, 509, 483, 460, 433. Elemental analysis: calc. (%) for $C_{41}H_{63}NO_6$ (665.956 g/mol): C (73.95%), H (9.54%), N (2.10%), O (14.41%); found: C (73.96%), H (9.54%), N (2.09%), O (14.42%).

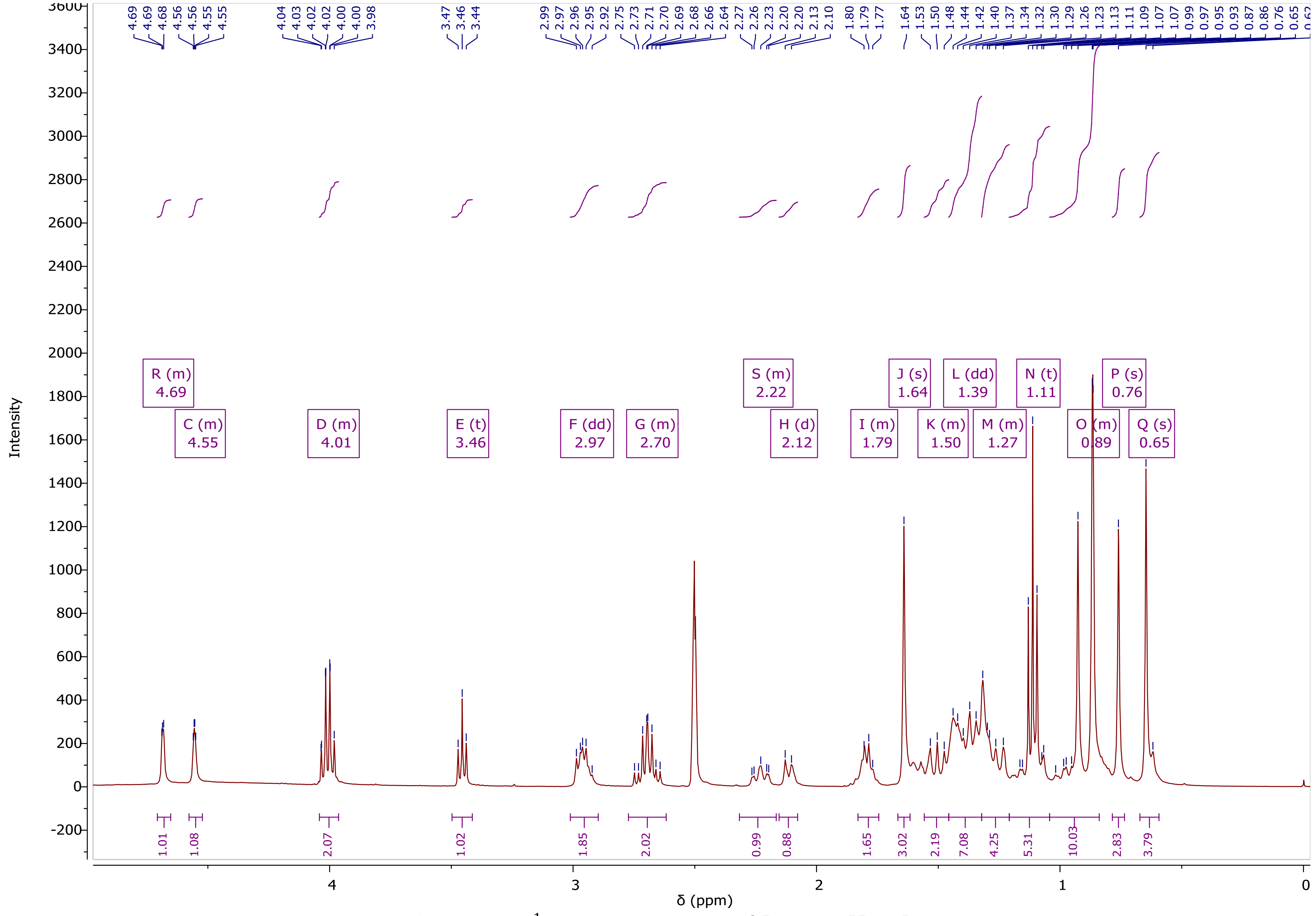

Figure S74. ^{1}H NMR spectrum of [TyrOEt][BA].

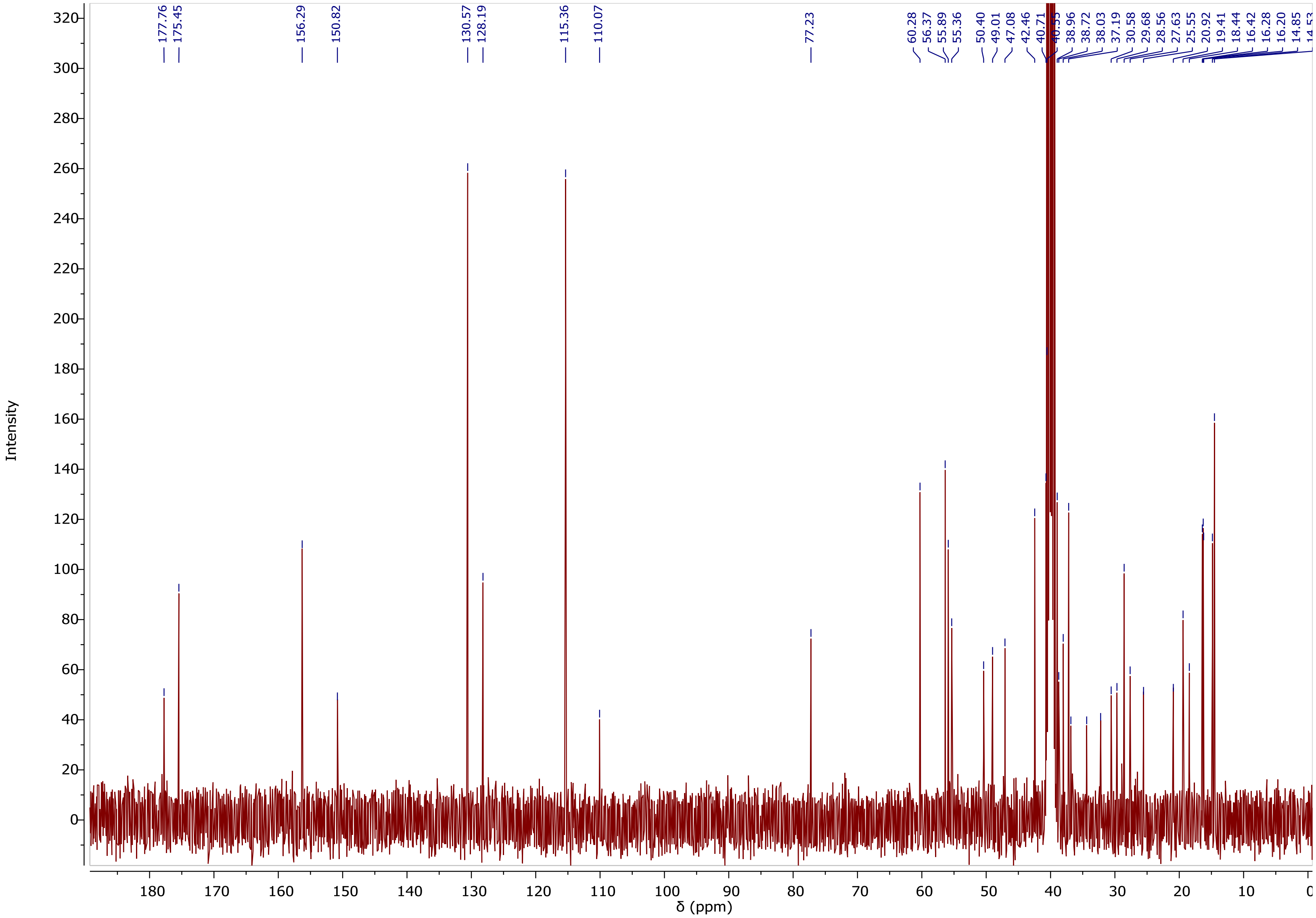

Figure S75. ^{13}C NMR spectrum of [TyrOEt][BA].

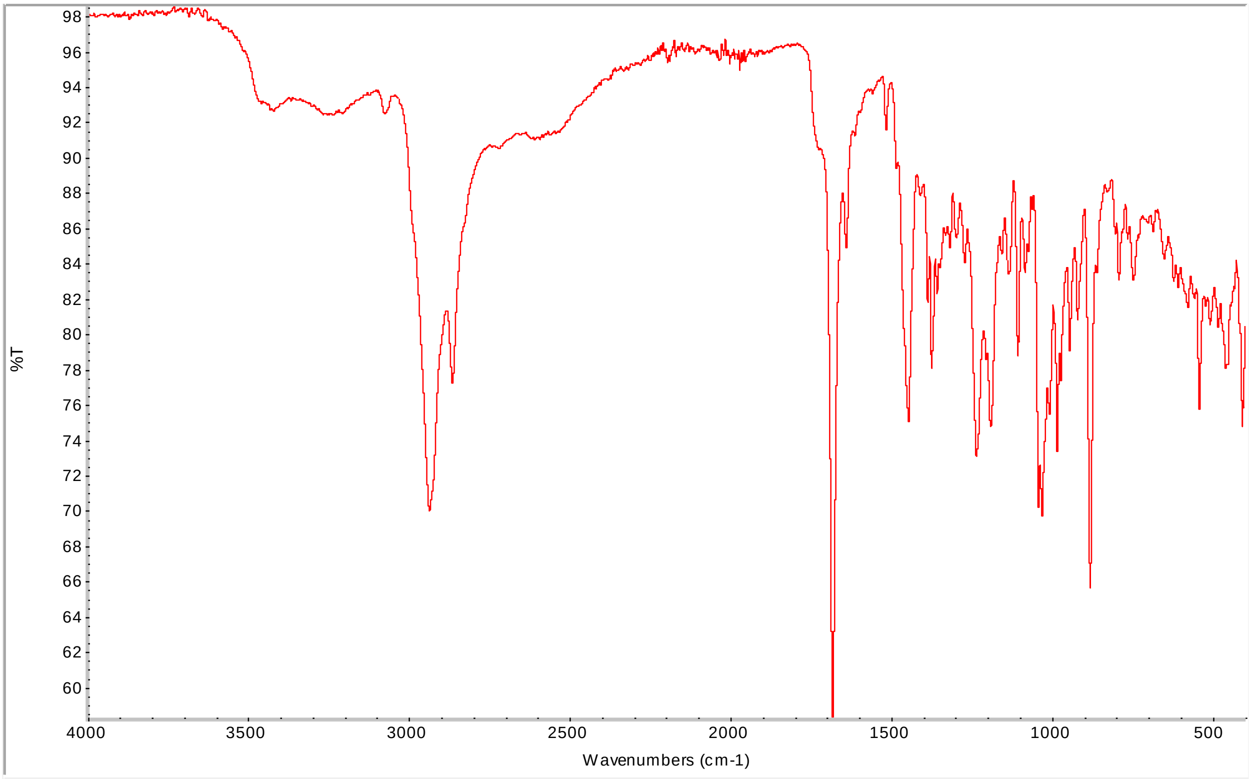

Figure S76. ATR-FTIR spectrum of [TyrOEt][BA].

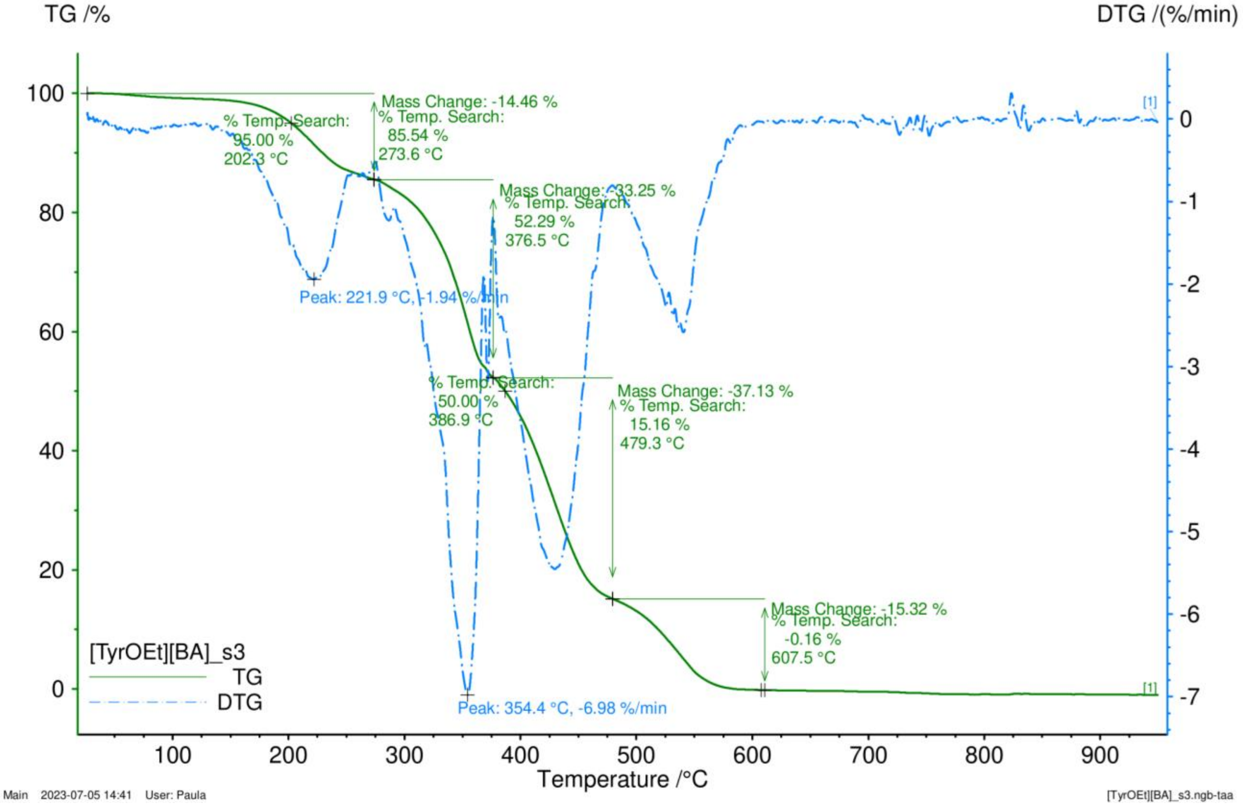

Figure S77. TG, DTG and c-DTA curves of [TyrOEt][BA].

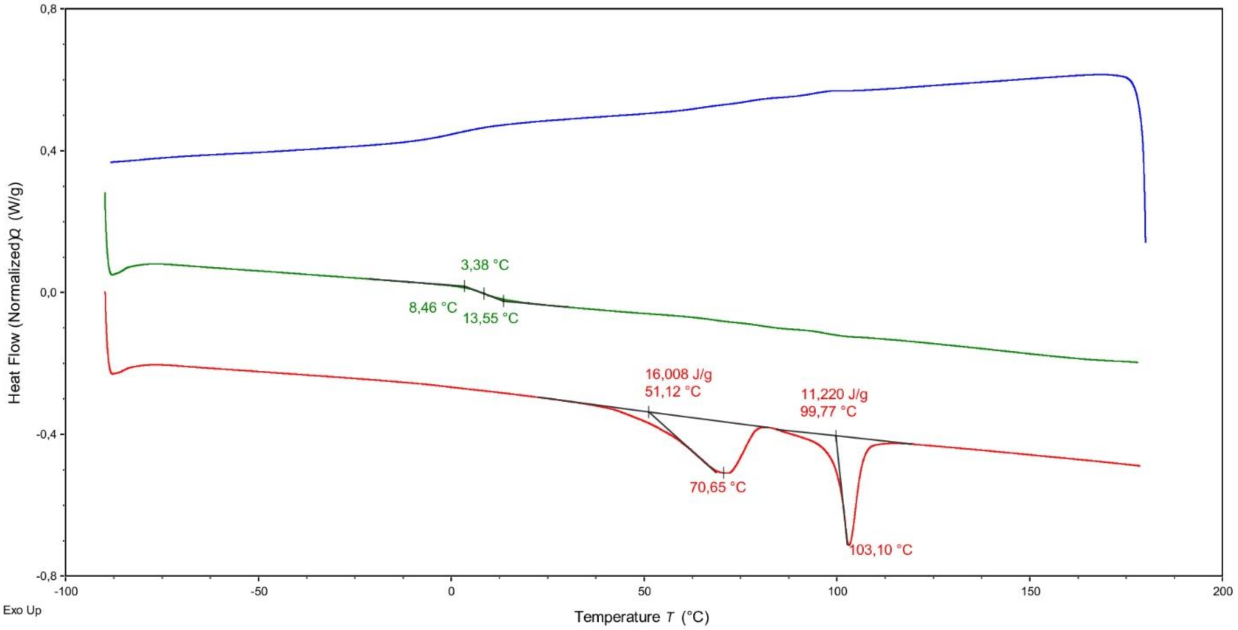

Figure S78. DSC curves of [TyrOEt][BA].

L-tryptophan ethyl ester betulinate - [TrpOEt][BA]

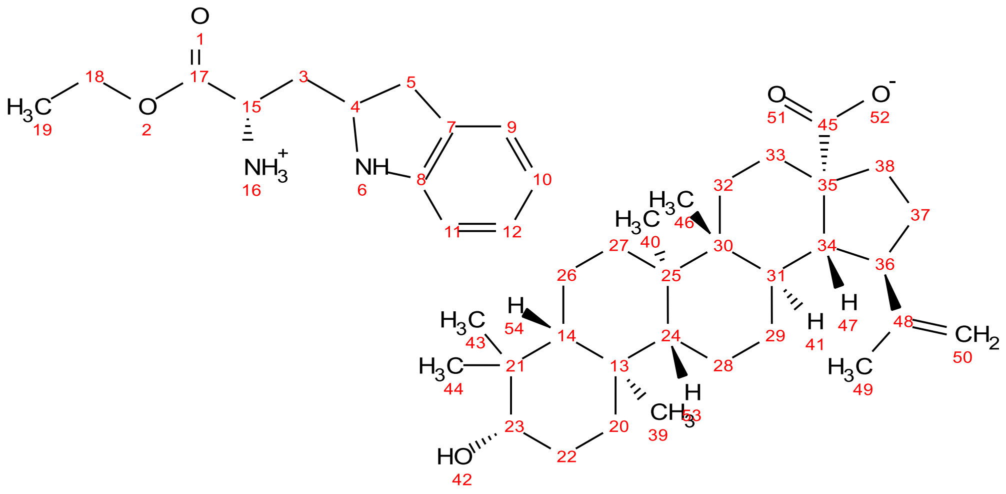

^{1}H NMR (400 MHz, DMSO-d_6) δ 10.86 (s, 1H), 7.49 (dd, J = 7.8, 1.1 Hz, 1H), 7.33 (dt, J = 8.1, 0.9 Hz, 1H), 7.11 (d, J = 2.4 Hz, 1H), 7.05 (ddd, J = 8.1, 7.0, 1.2 Hz, 1H), 6.96 (ddd, J = 8.0, 7.0, 1.1 Hz, 1H), 4.70 – 4.67 (m, 1H), 4.57 – 4.54 (m, 1H), 4.03 – 3.94 (m, 2H), 3.60 (t, J = 6.4 Hz, 1H), 3.06 – 2.88 (m, 4H), 2.27 – 2.18 (m, 1H), 2.16 – 2.08 (m, 1H), 1.80 (q, J = 7.6, 6.3 Hz, 2H), 1.64 (s, 4H), 1.60 – 1.42 (m, 6H), 1.42 – 1.21 (m, 10H), 1.09 (t, J = 7.1 Hz, 6H), 0.93 (s, 4H), 0.87 (d, J = 2.0 Hz, 8H), 0.76 (s, 3H), 0.65 (s, 4H). ^{13}C NMR (100 MHz, DMSO-d_6) δ 177.76, 175.63, 150.80, 136.54, 127.87, 124.08, 121.29, 118.73, 118.69, 111.78, 110.37, 110.08, 77.23, 60.32, 55.89, 55.62, 55.36, 50.40, 49.01, 47.09, 42.46, 40.71, 38.96, 38.73, 38.04, 37.19, 36.84, 34.39, 32.22, 31.23, 30.58, 29.68, 28.57, 27.63, 25.55, 20.93, 19.41, 18.44, 16.42, 16.28, 16.20, 14.85, 14.46. FT–IR: ν (ATR): 3411, 3250, 3073, 2938, 2867, 2619, 1729, 1683, 1642, 1585, 1446, 1387, 1358, 1319, 1298, 1272, 1234, 1190, 1157, 1136, 1106, 1074, 1043, 1031, 1009, 983, 972, 944, 920, 881, 860, 793, 740, 704, 685, 651, 621, 608, 577, 556, 542, 607, 482, 459, 426. Elemental analysis: calc. (%) for $C_{43}H_{66}N_2O_5$ (691.010 g/mol): C (74.74), H (9.63), N (4.05), O (11.58); found: C (74.73), H (9.64), N (4.05), O (11.59).

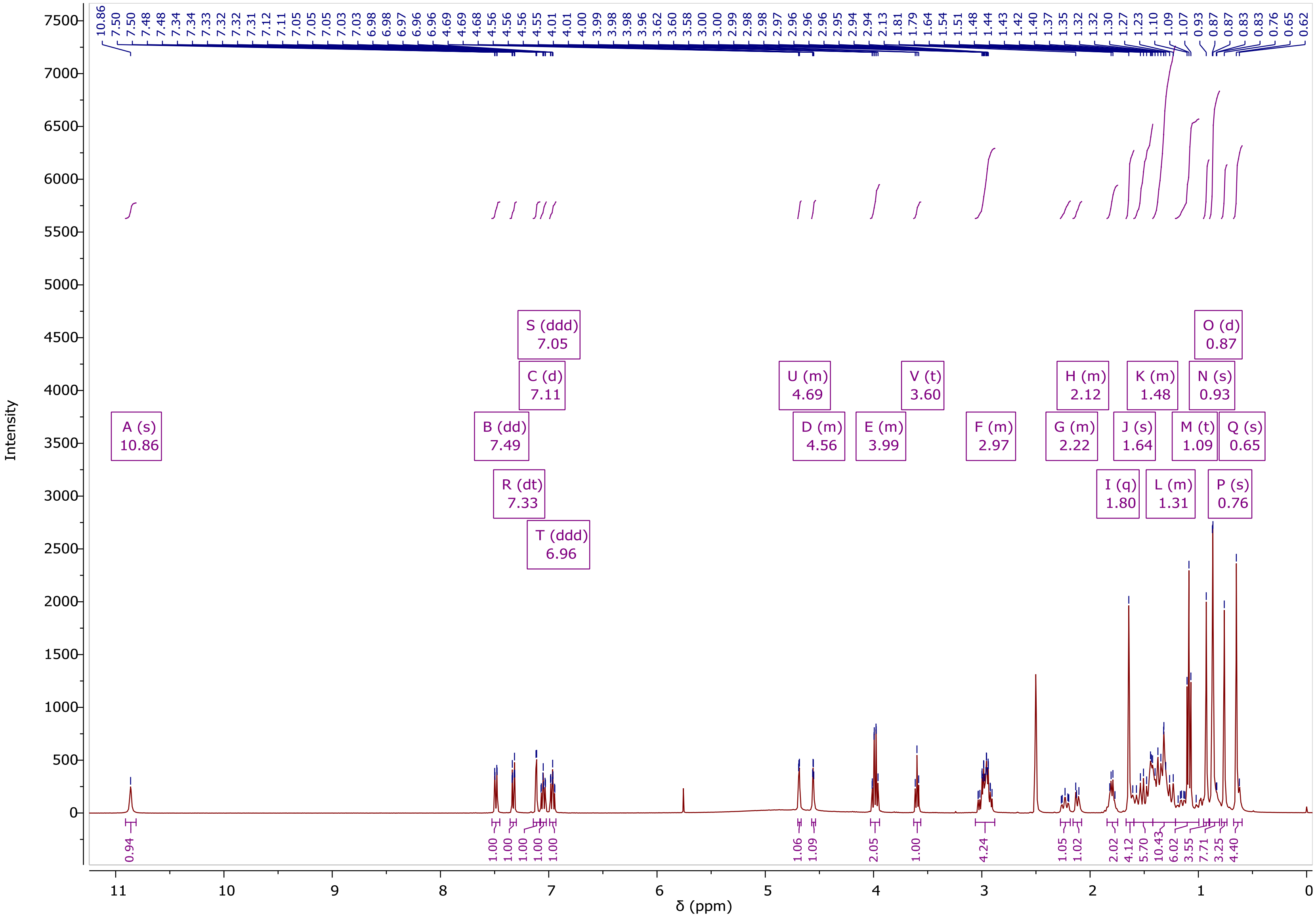

Figure S79. ^{1}H NMR spectrum of [TrpOEt][BA].

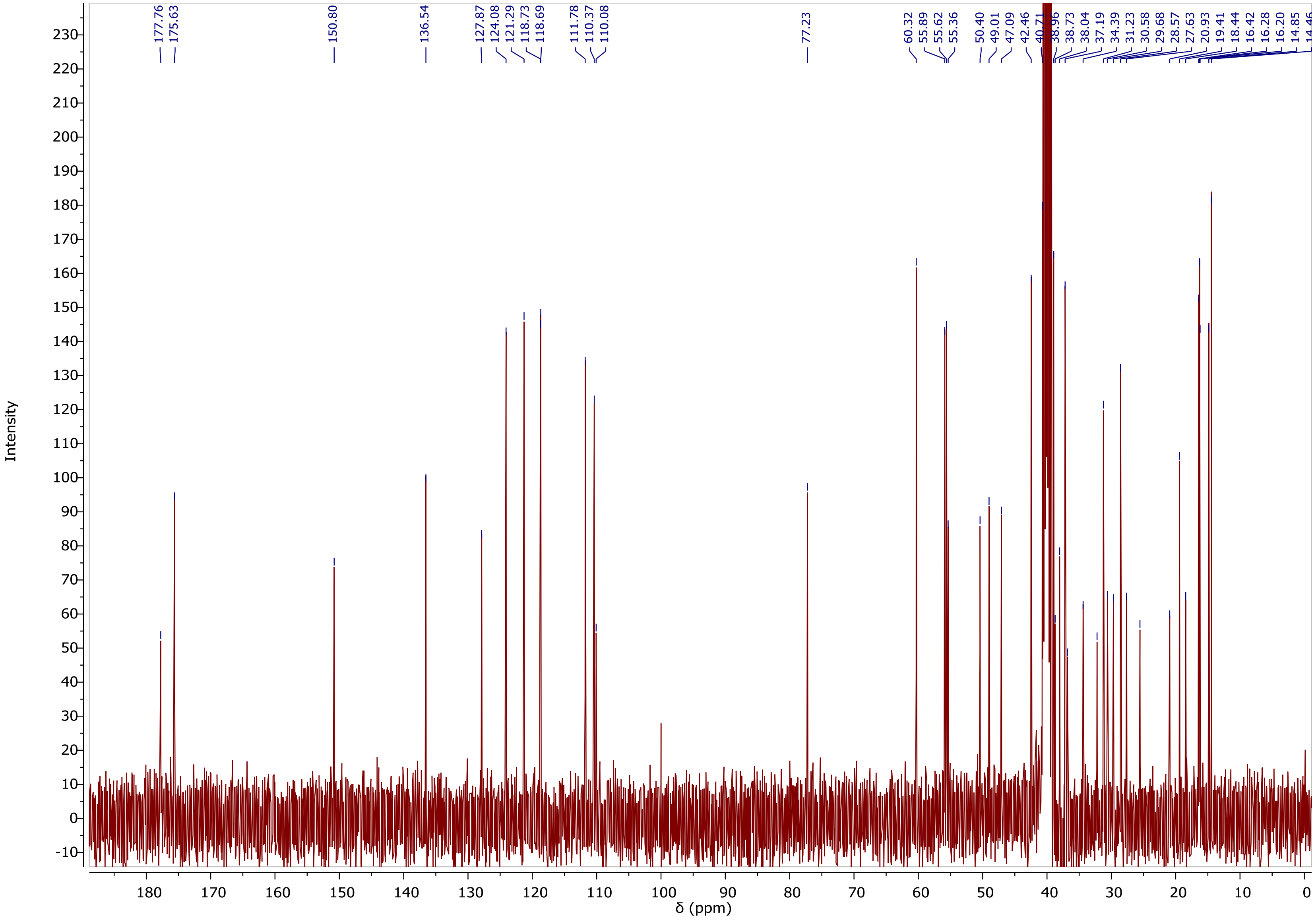

Figure S80. ^{13}C NMR spectrum of [TrpOEt][BA].

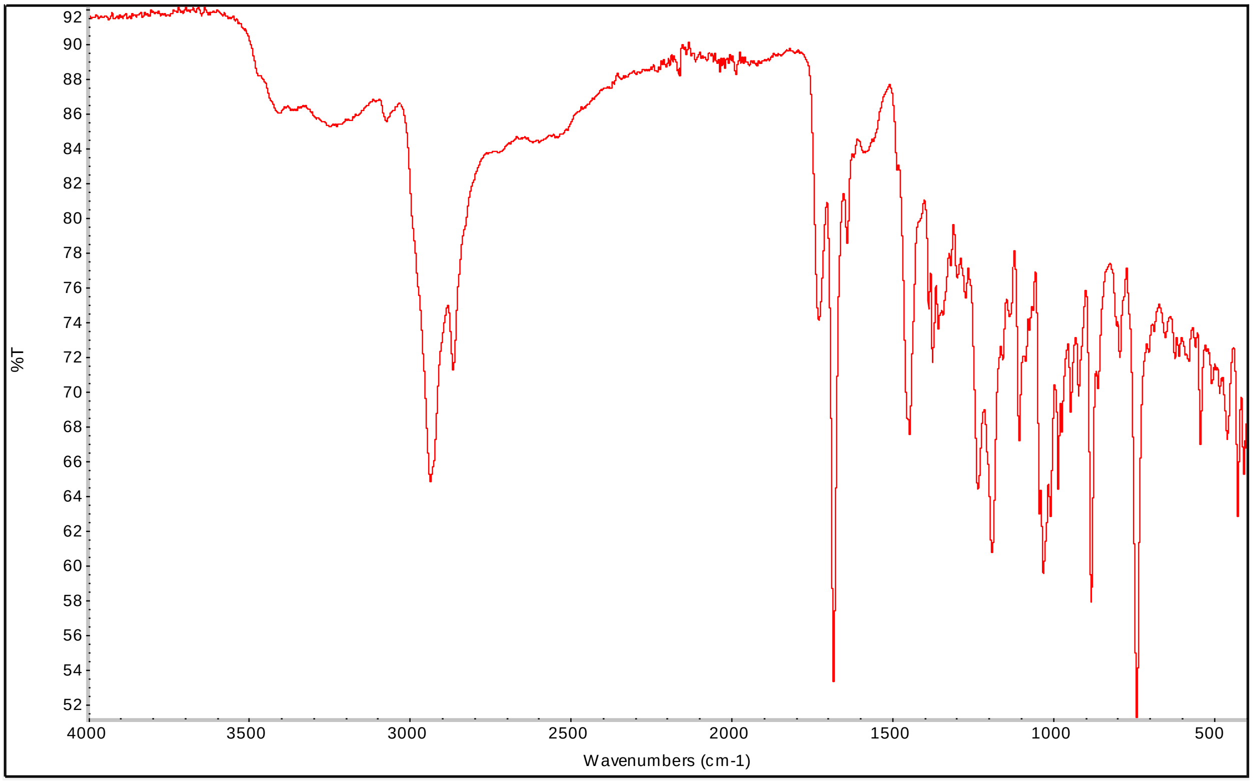

Figure S81. ATR-FTIR spectrum of [TrpOEt][BA].

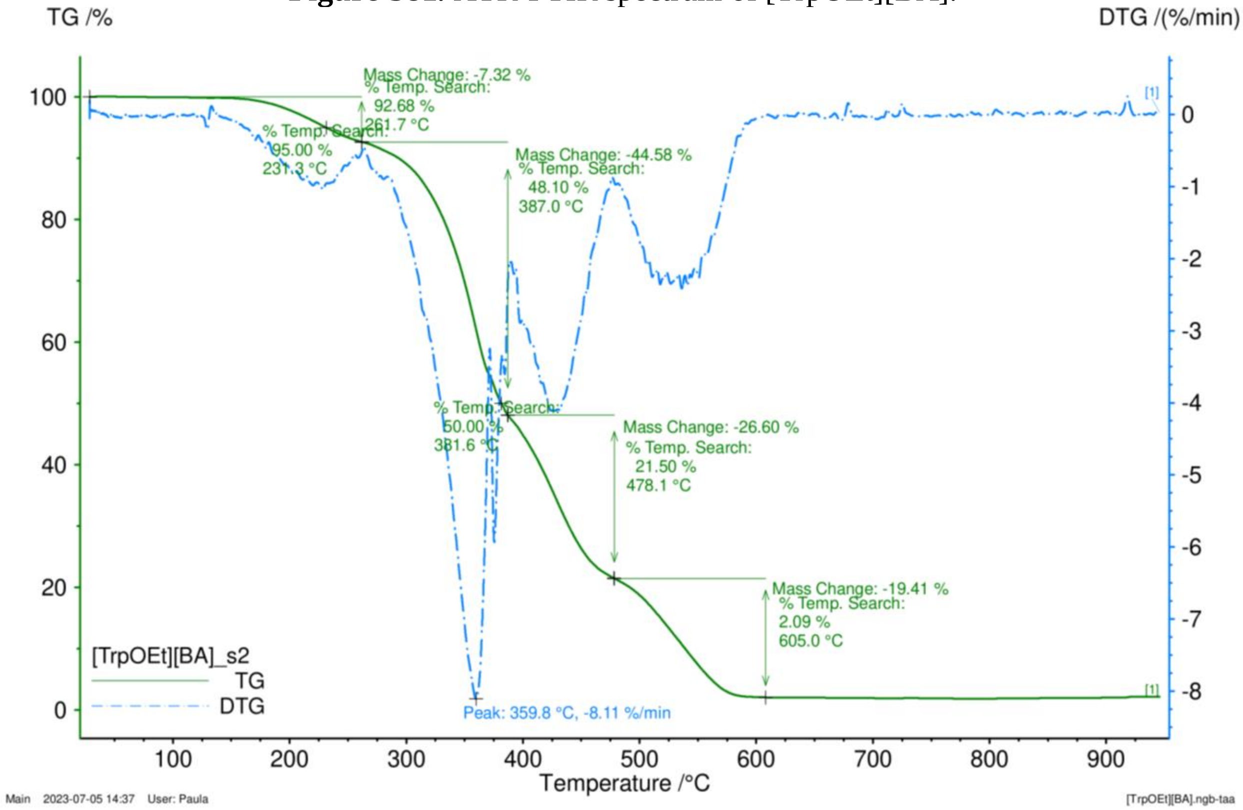

Figure S82. TG, DTG and c-DTA curves of [TrpOEt][BA].

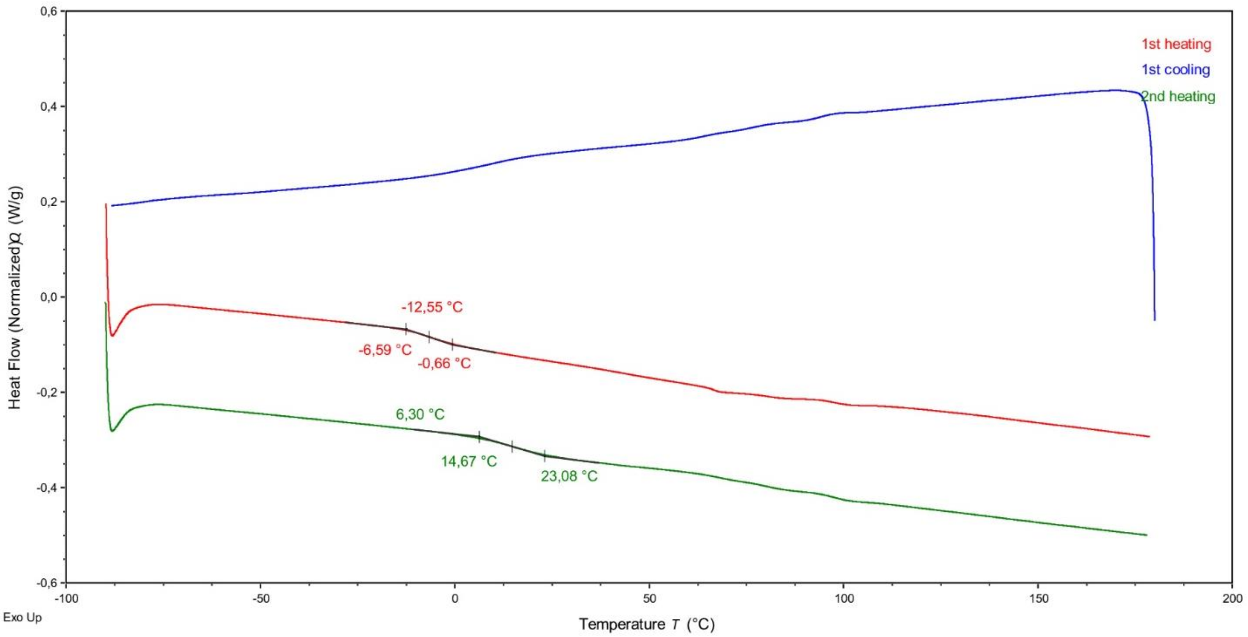

Figure S83. DSC curves of [TrpOEt][BA].

L-aspartic acid ethyl ester betulinate - [Asp(OEt)$_2$][BA]

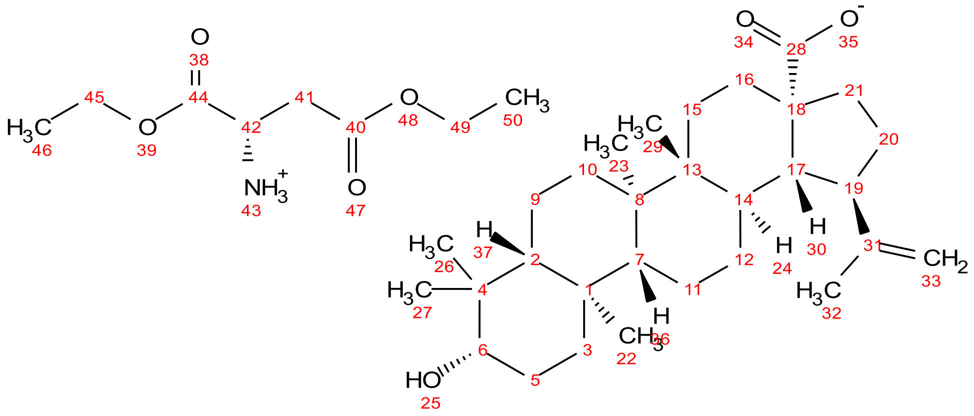

^{1}H NMR (400 MHz, DMSO-*d*$_6$) δ 4.69 (d, *J* = 2.5 Hz, 1H), 4.57 – 4.55 (m, 1H), 4.10 – 4.02 (m, 4H), 3.62 (t, *J* = 6.4 Hz, 1H), 2.96 (dt, *J* = 11.6, 6.0 Hz, 2H), 2.65 – 2.52 (m, 2H), 2.27 – 2.19 (m, 1H), 2.11 (d, *J* = 10.0 Hz, 1H), 1.79 (q, *J* = 6.9, 5.3 Hz, 2H), 1.64 (s, 4H), 1.59 (d, *J* = 12.0 Hz, 2H), 1.55 – 1.41 (m, 6H), 1.36 (d, *J* = 10.3 Hz, 4H), 1.32 (s, 4H), 1.29 – 1.21 (m, 2H), 1.17 (td, *J* = 7.1, 3.7 Hz, 7H), 1.15 – 0.99 (m, 3H), 0.93 (s, 4H), 0.89 – 0.84 (m, 7H), 0.76 (s, 3H), 0.65 (s, 4H). ^{13}C NMR (100 MHz, DMSO-*d*$_6$) δ 177.74, 174.66, 171.15, 150.80, 110.08, 77.23, 60.71, 60.44, 55.89, 55.35, 51.56, 50.39, 49.00, 47.08, 42.46, 40.71, 38.96, 38.72, 38.03, 37.19, 36.82, 34.38, 32.20, 30.57, 29.67, 28.56, 27.63, 25.55, 20.91, 19.41, 18.43, 16.42, 16.27, 16.20, 14.85, 14.51. FT–IR: *ν* (ATR): 3335, 3255, 3079, 2965, 2926, 2864, 1740, 1701, 1683, 1641, 1483, 1446, 1431, 1386, 1372, 1360, 1330, 1302, 1259, 1227, 1185, 1156, 1102, 1083, 1032, 1010, 984, 972, 944, 920, 883, 864, 792, 766, 749, 650, 625, 603, 574, 562, 546, 511, 482, 454, 433. Elemental analysis: calc. (%) for $C_{38}H_{63}NO_7$ (645.922 g/mol): C (70.66), H (9.83), N (2.17), O (17.34); found: C (70.65), H (9.82), N (2.17), O (17.35).

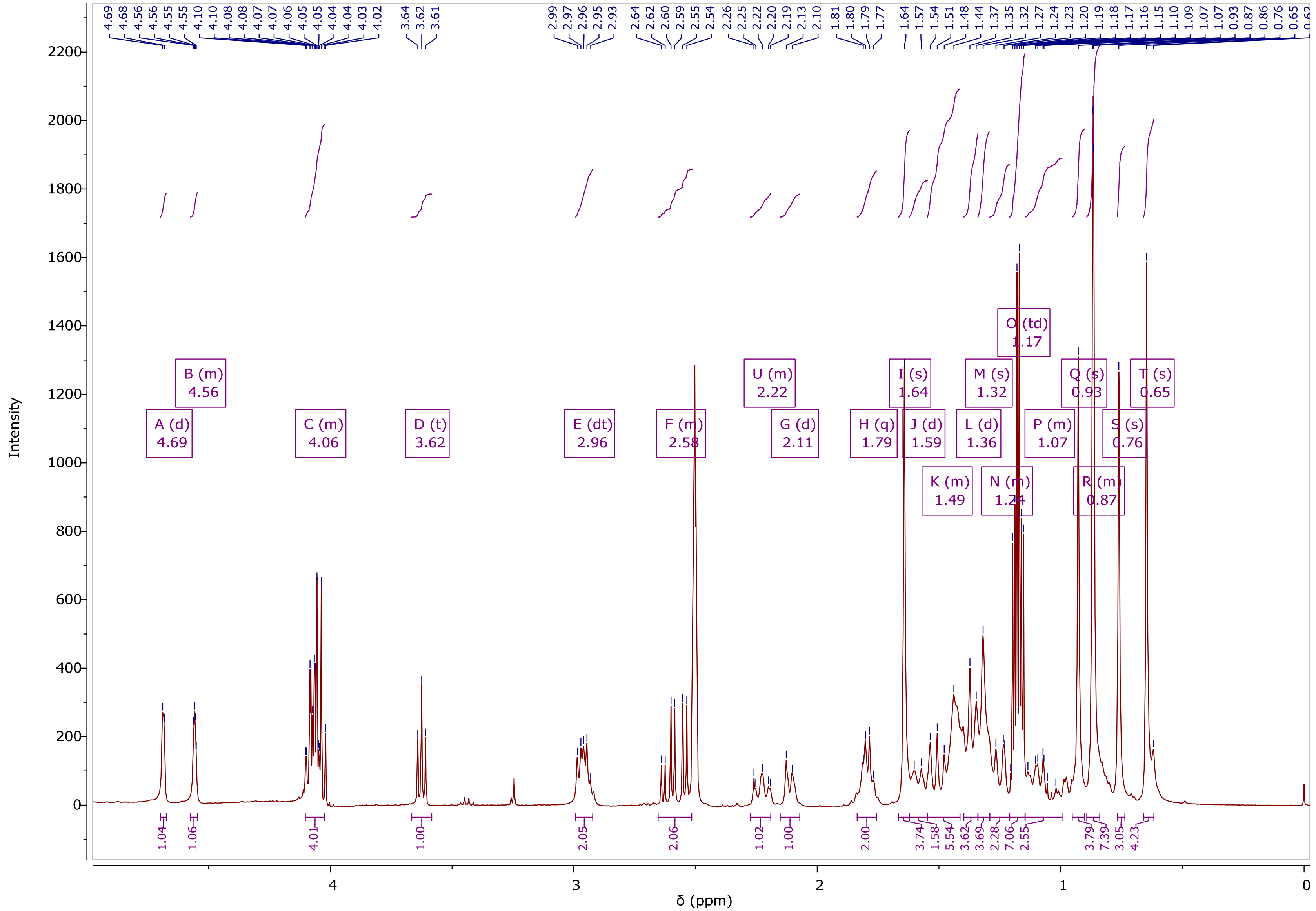

Figure S84. ^{1}H NMR spectrum of [Asp(OEt)$_2$][BA].

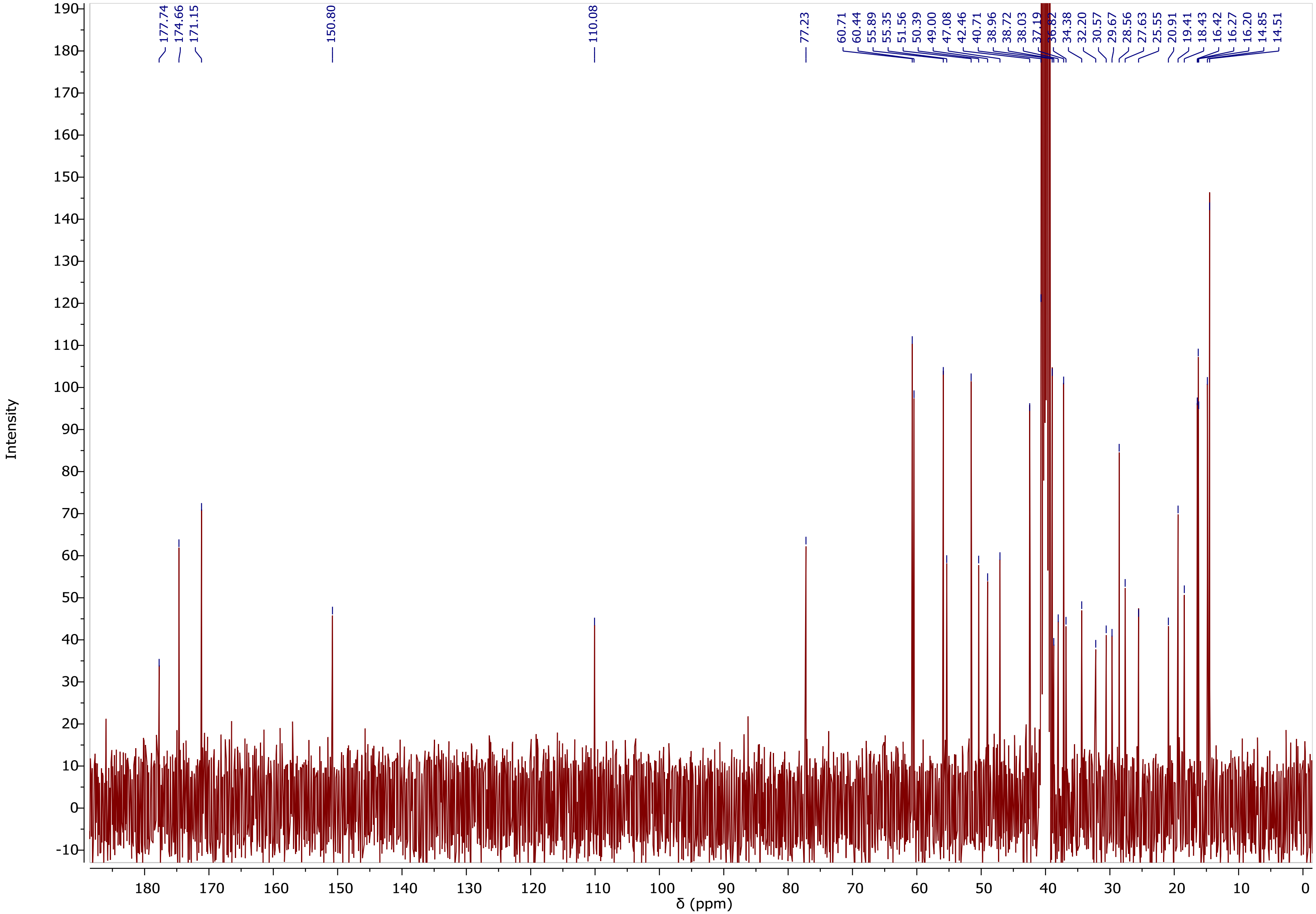

Figure S85. ^{13}C NMR spectrum of [Asp(OEt)$_2$][BA].

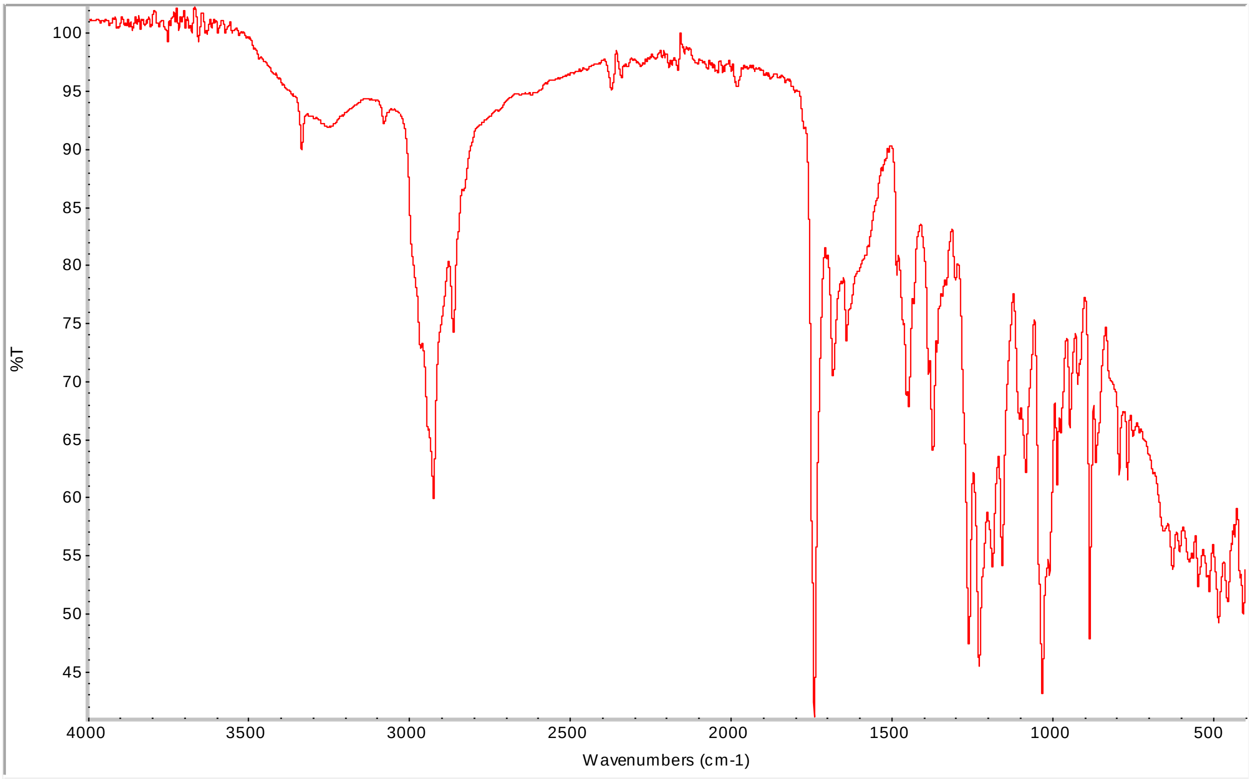

Figure S86. ATR-FTIR spectrum of [Asp(OEt)$_2$][BA].

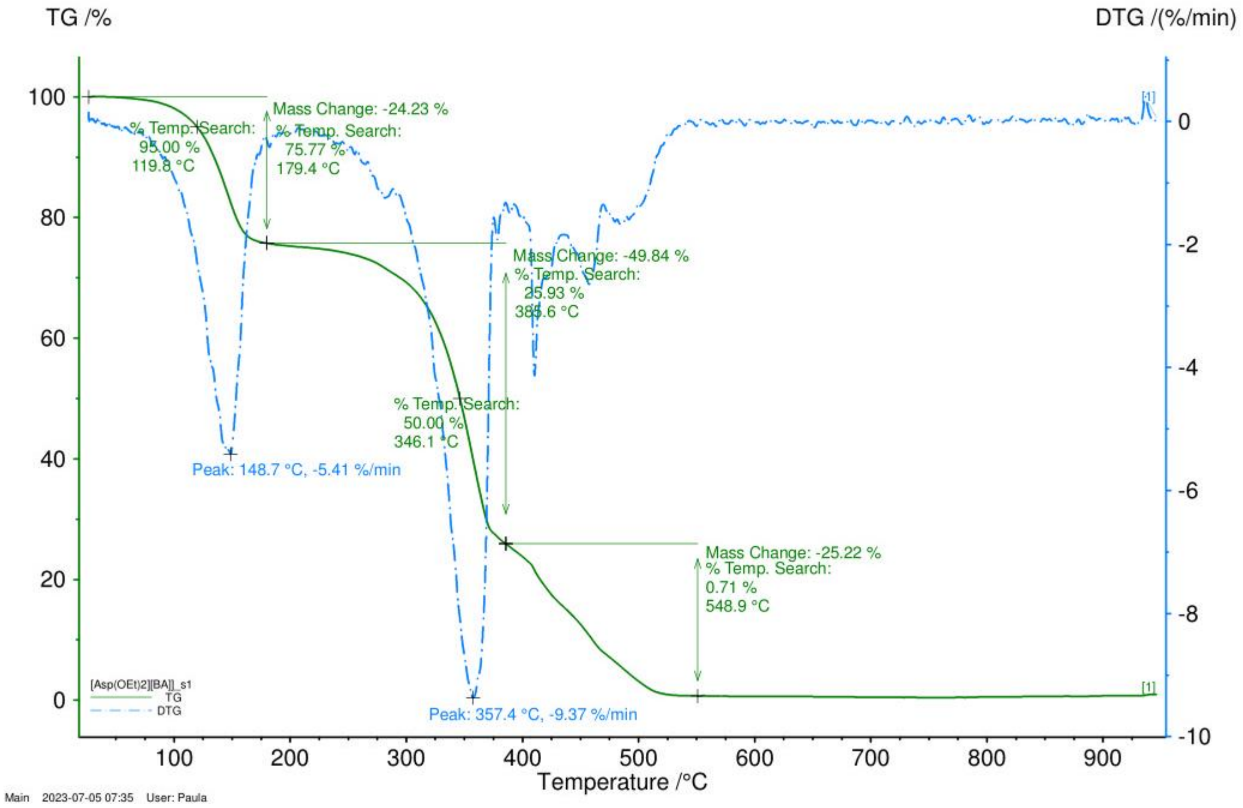

Figure S87. TG, DTG and c-DTA curves of [Asp(OEt)$_2$][BA].

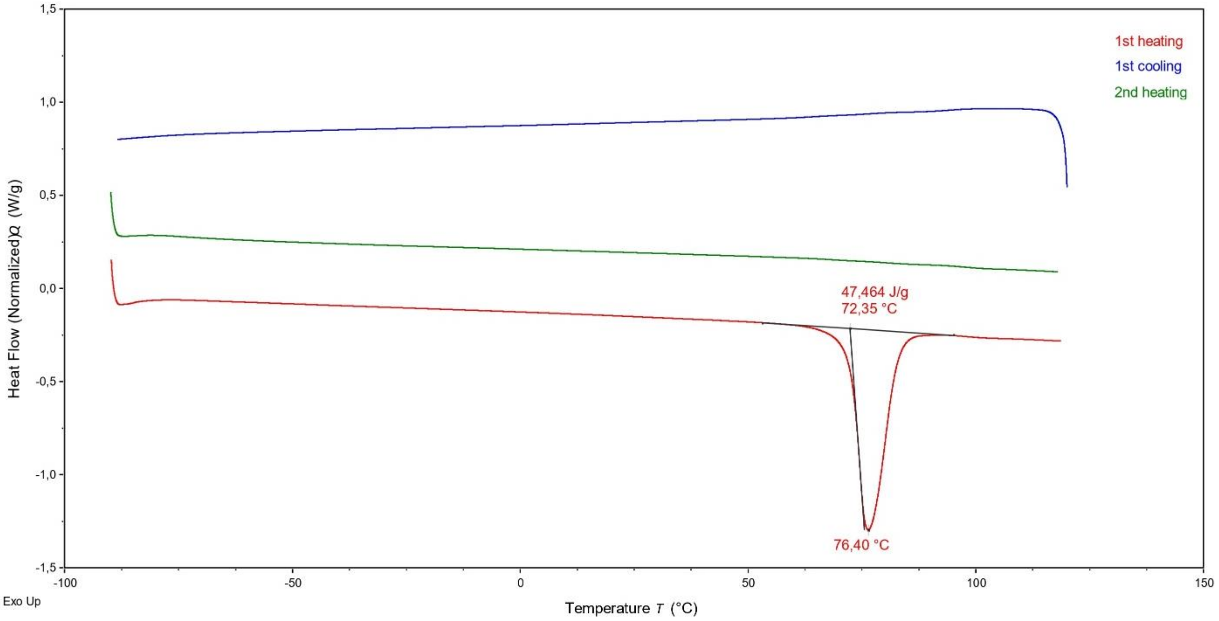

Figure S88. DSC curves of [Asp(OEt)$_2$][BA].
